# Characterising a homozygous two‐exon deletion in *UQCRH*: comparing human and mouse phenotypes

**DOI:** 10.15252/emmm.202114397

**Published:** 2021-11-08

**Authors:** Silvia Vidali, Raffaele Gerlini, Kyle Thompson, Jill E Urquhart, Jana Meisterknecht, Juan Antonio Aguilar‐Pimentel, Oana V Amarie, Lore Becker, Catherine Breen, Julia Calzada‐Wack, Nirav F Chhabra, Yi‐Li Cho, Patricia da Silva‐Buttkus, René G Feichtinger, Kristine Gampe, Lillian Garrett, Kai P Hoefig, Sabine M Hölter, Elisabeth Jameson, Tanja Klein‐Rodewald, Stefanie Leuchtenberger, Susan Marschall, Philipp Mayer‐Kuckuk, Gregor Miller, Manuela A Oestereicher, Kristina Pfannes, Birgit Rathkolb, Jan Rozman, Charlotte Sanders, Nadine Spielmann, Claudia Stoeger, Marten Szibor, Irina Treise, John H Walter, Wolfgang Wurst, Johannes A Mayr, Helmut Fuchs, Ulrich Gärtner, Ilka Wittig, Robert W Taylor, William G Newman, Holger Prokisch, Valerie Gailus‐Durner, Martin Hrabě de Angelis

**Affiliations:** ^1^ Institute of Human Genetics School of Medicine Technische Universität München Munich Germany; ^2^ Institute of Neurogenomics Helmholtz Zentrum München German Research Center for Environmental Health Neuherberg Germany; ^3^ Department of Pediatrics University Hospital Salzburg Paracelsus Medical University Salzburg Salzburg Austria; ^4^ Institute of Experimental Genetics and German Mouse Clinic Helmholtz Zentrum München German Research Center for Environmental Health Neuherberg Germany; ^5^ German Center for Diabetes Research (DZD) Neuherberg Germany; ^6^ Wellcome Centre for Mitochondrial Research Translational and Clinical Research Institute Faculty of Medical Sciences Newcastle University Newcastle upon Tyne UK; ^7^ Manchester Centre for Genomic Medicine Manchester University NHS Foundation Trust Manchester UK; ^8^ Functional Proteomics Institute for Cardiovascular Physiology Faculty of Medicine Goethe University Frankfurt Frankfurt Germany; ^9^ Institute of Developmental Genetics Helmholtz Zentrum München German Research Center for Environmental Health Neuherberg Germany; ^10^ Research Unit Molecular Immune Regulation Helmholtz Zentrum München German Research Center for Environmental Health Munich Germany; ^11^ Chair of Developmental Genetics TUM School of Life Sciences Technische Universität München Freising Germany; ^12^ Institute of Molecular Animal Breeding and Biotechnology Ludwig‐Maximilians University Munich Munich Germany; ^13^ Faculty of Medicine and Health Technology Tampere University Tampere Finland; ^14^ Department of Cardiothoracic Surgery Jena University Hospital Jena Germany; ^15^ Deutsches Institut für Neurodegenerative Erkrankungen (DZNE) Site Munich Munich Germany; ^16^ Institute for Anatomy and Cell Biology Justus‐Liebig‐University of Giessen Giessen Germany; ^17^ German Center for Cardiovascular Research (DZHK) Partner site RheinMain Frankfurt Germany; ^18^ Evolution and Genomic Sciences School of Biological Sciences University of Manchester Manchester UK; ^19^ Chair of Experimental Genetics TUM School of Life Sciences Technische Universität München Freising Germany; ^20^ Present address: Klinik und Poliklinik für Innere Medizin II Klinikum rechts der Isar Technische Universität München München Germany; ^21^ Present address: Institute of Molecular Genetics of the Czech Academy of Sciences Czech Centre for Phenogenomics Vestec Czech Republic

**Keywords:** complex III, mitochondrial disease, mouse model, OXPHOS, UQCRH, Genetics, Gene Therapy & Genetic Disease, Organelles

## Abstract

Mitochondrial disorders are clinically and genetically diverse, with isolated complex III (CIII) deficiency being relatively rare. Here, we describe two affected cousins, presenting with recurrent episodes of severe lactic acidosis, hyperammonaemia, hypoglycaemia and encephalopathy. Genetic investigations in both cases identified a homozygous deletion of exons 2 and 3 of *UQCRH*, which encodes a structural complex III (CIII) subunit. We generated a mouse model with the equivalent homozygous *Uqcrh* deletion (*Uqcrh* ^−/−^), which also presented with lactic acidosis and hyperammonaemia, but had a more severe, non‐episodic phenotype, resulting in failure to thrive and early death. The biochemical phenotypes observed in patient and *Uqcrh* ^−/−^ mouse tissues were remarkably similar, displaying impaired CIII activity, decreased molecular weight of fully assembled holoenzyme and an increase of an unexpected large supercomplex (S_XL_), comprising mostly of one complex I (CI) dimer and one CIII dimer. This phenotypic similarity along with lentiviral rescue experiments in patient fibroblasts verifies the pathogenicity of the shared genetic defect, demonstrating that the *Uqcrh* ^−/−^ mouse is a valuable model for future studies of human CIII deficiency.

## Graphical abstract

This work describes the first confirmed pathogenic variant in *UQCRH* in two children presenting with recurrent episodes of metabolic crisis. A mouse model harbouring the equivalent variant in Uqcrh shared similar biochemical phenotypes of impaired CIII activity and abnormal OXPHOS supercomplex structures, but with more severe manifestation.

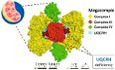

**The paper explained**

**Problem**

Mitochondrial diseases are a group of disorders and are varied in terms of both their clinical manifestations and their genetic causes. They are characterised by mitochondrial dysfunction, predominantly resulting in decreased energy production in the form of ATP made by the oxidative phosphorylation (OXPHOS) system. Mitochondrial diseases can be caused by changes within the mitochondrial genome itself (mtDNA), or in nuclear genes that encode mitochondrial proteins (≈1,200). More than 300 of these genes have been associated with mitochondrial disease, and this diversity often makes obtaining genetic diagnoses in affected individuals difficult. UQCRH is a mitochondrial protein encoded by the nuclear genome and is a subunit of complex III, one of 5 multi‐subunit complexes that make up the OXPHOS system. There have not been any previous reports of *UQCRH* variants in mitochondrial disease cases.

**Results**

Here, we describe two affected cousins who presented clinically with episodes of metabolic crisis, manifesting with severe lactic acidosis, high ammonia levels, low glucose levels and brain abnormalities. Between these episodes (that were each preceded by a mild viral infection), the affected individuals were otherwise healthy. Whole exome sequencing identified a 2.2 kb dropout of sequencing reads predicting a homozygous deletion of 2 out of the 4 coding regions of the gene, confirmed by Sanger sequencing (c.55‐528_243+473del). We could not identify any other individuals with variants in *UQCRH* so we opted to generate a mouse model with the equivalent *Uqcrh* deletion. The mice showed similar symptoms of lactic acidosis and high ammonia levels, though the presentation was more severe and was constant rather than episodic, leading to early death. Biochemically, skin fibroblasts from the affected human subjects and tissue from the mutated mice were strikingly similar, displaying impaired activity of complex III, no expression of the UQCRH/Uqcrh protein, a smaller size of assembled complex III, an increase in larger OXPHOS supercomplexes and decreased expression of other complex III subunits. This strongly suggested that the shared UQCRH deletion was the cause of these similar biochemical defects. Crucially, we also showed an amelioration of the complex III deficiency when introducing a wild‐type (normal) copy of *UQCRH* via a lentiviral delivery system, further demonstrating that the *UQCRH* deletion was causative of the disease.

**Impact**

This study documents the first cases of mitochondrial disease due to variants in *UQCRH*. Confirmation of this variant as causative enables pre‐symptomatic accurate diagnosis of at‐risk individuals in the family, demonstrating the clinical importance of providing a genetic diagnosis in mitochondrial disease cases. Furthermore, the viable mouse model described here represents a valuable organism for the study of complex III biology and mitochondrial impairment more generally. Indeed, this mouse holds great potential to be used for new therapeutic strategies targeting CIII defects or related mitochondrial disorders.

## Introduction

Mitochondrial diseases are a group of complex genetic disorders characterised by mitochondrial dysfunction that result in clinically heterogeneous presentations, mostly affecting tissues with high energy requirements (Gorman *et al*, 2016). Mitochondrial proteins are encoded by either the nuclear (involving > 1,000 genes) or the mitochondrial genome (mtDNA) (13 protein‐coding genes) giving vast genetic heterogeneity. In recent years, next‐generation sequencing (NGS) technologies, particularly whole exome sequencing (WES) have been successful in identifying an increasing number of mutations in genes, causing primary mitochondrial disorders (Calvo *et al*, 2012; Taylor *et al*, 2014; Wortmann *et al*, 2015; Kohda *et al*, 2016; Pronicka *et al*, 2016). To date, pathogenic variants in more than 330 genes have been associated with mitochondrial disorders (Mayr *et al*, 2015; Alston *et al*, 2017; Frazier *et al*, 2019; Thompson *et al*, 2020).

The ubiquinol‐cytochrome *c* oxidoreductase or *bc1* complex (Complex III; CIII) is one of the five complexes of the mitochondrial oxidative phosphorylation (OXPHOS) system. CIII transports electrons from ubiquinol to cytochrome *c* and translocates protons across the inner mitochondrial membrane, contributing to the electrochemical gradient that is then used by complex V to drive ATP synthesis. In vertebrates, CIII comprises 11 subunits assembled with the support of at least four additional factors (Ghezzi & Zeviani, 2012; Sanchez *et al*, 2013; Singhal *et al*, 2017). Three genes encode the catalytic centres of CIII (Tucker *et al*, 2013; Wanschers *et al*, 2014; Koch *et al*, 2015; Feichtinger, Brunner‐Krainz, *et al*, 2017), namely *MT‐CYB* (encoding cytochrome *b*), *CYC1* (encoding cytochrome *c1*) and *UQCRFS1* (encoding the Rieske protein containing an iron‐sulphur cluster centre), while the exact function of the other eight (UQCRC1, UQCRC2, UQCRH, UQCRB, UQCRQ, cytochrome *b‐c1* complex subunit 9, UQCR10, and UQCR11) remains to be fully elucidated (Fernández‐Vizarra & Zeviani, 2015). Functional CIII is organised as a dimeric complex (CIII_2_) and UQCRH is thought to be a structural component linking the cytochromes *c* and *c1* (Ohta *et al*, 1987).

Mitochondrial respiratory chain enzymes assemble with varying stoichiometry, with CIII, CI, and CIV supramolecular structures called supercomplexes (SCs; Schägger & Pfeiffer, 2000). CI_1_:CIII_2_:CIV_1_ is generally considered the smallest functional unit often referred to as the “respirasome”. SCs facilitate the transfer of electrons from NADH to O_2_. Every complex stabilises the others and, therefore, defects in a single component often result in a general instability of the complexes and, potentially, of the respirasome. Indeed, deficiency of CIII (Acin‐Perez *et al*, 2004; Protasoni *et al*, 2020) or CIV (Diaz *et al*, 2006) usually leads to impaired activity of CI.

CIII defects are among the least frequently diagnosed isolated respiratory chain complex deficiencies in mitochondrial disorders (Ferná>ndez‐Vizarra & Zeviani, 2015). Pathogenic variants in eleven genes have been reported in association with CIII deficiency. Among those, five encode subunits of CIII (Haut *et al*, 2003; Barel *et al*, 2008; Gasparre *et al*, 2008; Gaignard *et al*, 2013; Miyake *et al*, 2013; Feichtinger, Brunner‐Krainz, *et al*, 2017), three encode CIII assembly factors (Tucker *et al*, 2013; Wanschers *et al*, 2014; Koch *et al*, 2015; Feichtinger, Brunner‐Krainz, *et al*, 2017) and three encode factors involved in the loading of iron‐sulphur clusters (2Fe‐2S) to the Rieske protein (Moran *et al*, 2010; Invernizzi *et al*, 2013; Kremer *et al*, 2016; Gusic *et al*, 2020). Previously reported CIII deficiency disorders are all autosomal recessive, with the exception of *MT‐CYB* which is encoded by mtDNA and thus follows maternal inheritance or can manifest *de novo (*Andreu *et al*, 1999; Hagen *et al*, 2013; Chaussenot *et al*, 2018 *)*. The clinical phenotypes associated with CIII deficiency are heterogeneous, but the most common clinical presentations are early‐onset recurrent metabolic crises characterised by lactic acidosis and hypoglycaemia (Feichtinger, Brunner‐Krainz, *et al*, 2017). For instance, cytochrome *c1* variants were described in two patients with early‐onset episodic acute metabolic ketoacidosis, lactic acidosis, hyperammonaemia and insulin‐responsive hyperglycaemia triggered by infection and febrile illness during childhood (Morison *et al*, 2008; Gaignard *et al*, 2013; De Rocco *et al*, 2014). Similarly, a homozygous 4‐bp deletion in the ubiquinol‐cytochrome *c* reductase binding protein (*UQCRB*), observed in only one patient, was associated with episodic metabolic decompensation (a derangement of normal metabolism characterised by hypoglycaemia, lactic acidosis, and transient liver dysfunction (Haut *et al*, 2003)). Furthermore, pathogenic variants identified in cytochrome *b‐c1* complex subunit 2 (*UQCRC2*) have been associated with episodic severe metabolic acidosis and hyperammonaemia, hypoglycaemia and lactic acidosis in four patients (Gaignard *et al*, 2017). Interestingly, the generation of a full‐body mouse model of CIII structural subunit deficiency has not yet been successful, with previous attempts such as the knockout of the mouse homologue of *UQCRB* displaying embryonic lethality (Dickinson *et al*, 2016).

Here, we report the clinical and molecular findings of two first cousin British Pakistani children presenting with recurrent episodic manifestations of severe lactic acidosis, excess blood ammonia and hypoglycaemia leading to episodes of encephalopathy. Both individuals were found to have a homozygous two‐exon deletion in *UQCRH*, which we reproduced in a mouse model allowing the comparison of clinical and biochemical phenotypes for deeper characterisation.

## Results

### Case reports

**Figure 1:**
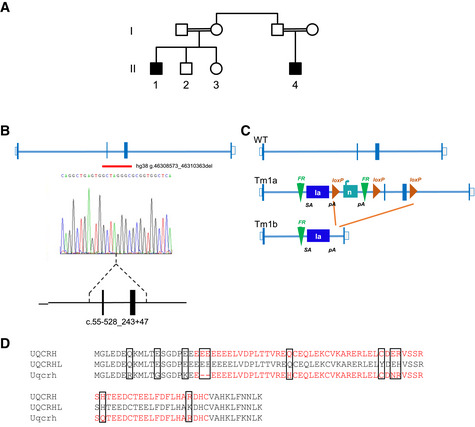
Identification of patients with a two‐exon deletion *UQCRH* and generation of a mouse model with the equivalent deletion of *Uqcrh* Pedigree of the family carrying a two‐exon deletion of *UQCRH*. Affected individuals (black‐filled squares) present with episodes of vomiting, metabolic acidosis, high lactate and hypoglycaemia.Position of the identified *UQCRH* deletion in the two affected patients. Empty bars, untranslated region (UTR); filled bars, translated sequence; thin blue lines, intron; red line underlines the two deleted exons.Scheme of the genomic targeting strategy by a Cre recombinase resulting in the deletion of two flanking loxP exons in the *Uqcrh* gene of the mouse model. SA indicates splice acceptor site; pA, poly‐A.Alignment of amino acids of human *UQCRH*, human pseudogene *UQCRHL* and mouse *Uqcrh*. In red, the deleted amino acids in human and mouse. The black boxes indicate variation in amino acid residues.

The proband (II‐1), a male infant, is one of three children of healthy, consanguineous, British/Pakistani parents (Fig 1A). At the age of two years, following a mild undetermined viral illness, he presented with vomiting, metabolic acidosis and raised plasma lactate level. He recovered well upon intravenous fluid replacement. He then presented similar episodes three or four times per year, usually following a mild upper respiratory tract infection, likely viral, with vomiting and was resuscitated with fluids on each occasion. On eight occasions, he presented at the hospital with metabolic acidosis (lowest pH 7.2), showing increased lactate levels (up to 18.2 mM, normal range 0.7–2.2 mM), and hypoglycaemia (lowest glucose 1.0 mM, normal 4.0–5.9 mM). Ammonia levels were also increased (up to 301 μM, normal 11–32 μM), but decreased rapidly upon fluid replacement. Liver function tests were within the normal range. Over the last four years of follow‐up (age 8–12 years), the patient presented twice with vomiting without metabolic decompensation, he was well in between episodes and developmental progress appeared to be normal. His clinically unaffected siblings did not experience similar episodes.

The second affected individual (II‐4) is the only child of consanguineous parents and is the first cousin of the proband (II‐1). He was well until the age of 3 years and 4 months when he presented to the hospital with vomiting and diarrhoea, high lactate and compensated metabolic acidosis. Over the next three years, he presented an average number of eight times per year to the hospital, with vomiting, fever or abdominal pain, and was treated with intravenous fluids. He experienced high lactate levels on four occasions (maximum 10 mM). On one occasion, at the age of 4 years and 8 months, he presented with a pH of 7.046, lactate of 10 mM, ammonia of 306 μM and blood glucose of 0.5 mM. Apart from a single episode of pancreatitis, the patient developed normally. At his last follow‐up visit, he was 8 years old, since then he showed no episodes of metabolic crisis for 2 years. A comprehensive clinical history of both patients is provided in Appendix Table S1.

### Molecular genetic investigations

**Figure EV1:**
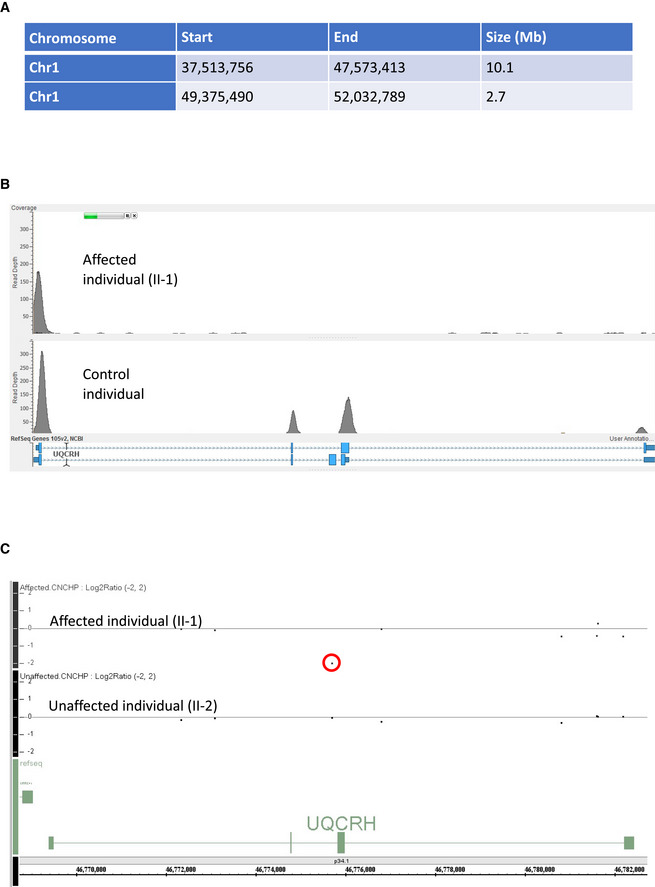
Exome sequence analysis Table detailing the homozygous regions identified in the two affected first cousin individuals.Read depth image from exome sequence data from the affected individual (II‐1) demonstrating absences of sequence reads encompassing exons 2 and 3 of UQCRH (upper panel) and evidence of sequence reads for both exons in an unrelated control (lower panel).Log_2_ ratio SNP microarray results show the absence of signal for a single probe between exons two and three of UQCRH (indicated by a red circle) in an affected individual (II‐1) upper track and evidence of two copies of this probe in their unaffected sibling (II‐2) in the lower track.

Autozygosity mapping in samples from four individuals (II‐1‐4 in Fig 1A) revealed only two homozygous regions (> 2 Mb) common to the two affected individuals, which were absent in the unaffected individuals. Both of these homozygous regions of 10.1 and 2.7 Mb were found on chromosome 1 (Fig EV1A). WES was undertaken on a DNA sample from the proband (II‐1) and achieved 20‐fold coverage of 86.7%. Analysis filtering for homozygous single‐nucleotide variants in the autologous regions revealed two potential variants of interest, *HIVEP3* (NM_024503) c.7043C>T; p. Pro2348Leu and *TCTEX1D4* (NM_001013632) c.140C>T; p. Pro47Leu. Neither of these variants were likely pathogenic due to presence in control populations (in gnomAD, the HIVEP3 variant was present in 130 out of 181,612 alleles including 1 homozygote and the *TCTEX1D4* variant present in 1,679 alleles out of 128,458 including 19 homozygotes) and were predicted benign using *in silico* tools. However, WES coverage data (Fig EV1B) indicated that both affected individuals carried a 2.2 kb homozygous deletion of exons 2 and 3 of *UQCRH* GRCh37/hg19, chr1:g.46,774,245‐46,776,461 (Fig 1B). This was supported by a review of the SNP array data, which revealed an absence of signal from the single probe within this region (Fig EV1C). Sanger sequencing defined the breakpoints of the deletion c.55‐528_243 + 473del predicted to culminate in an in‐frame deletion exons 2 and 3 of the four‐exon *UQCRH* gene, resulting in a shortened product. Segregation analysis genotyping revealed that the parents were heterozygous carriers of the deletion, as expected. The unaffected siblings, II‐2 and II‐3, were wild‐type and heterozygous for the deletion, respectively.

### Generation and clinical phenotyping of *Uqcrh* ^−/−^ mouse model

Screening of > 4,000 exomes from patients with suspected mitochondrial disorders for rare biallelic variants in *UQCRH* did not reveal additional cases, nor were any further families identified using GeneMatcher (Sobreira *et al*, 2015). Therefore, to explore the underlying molecular alterations, we made use of a mouse with a deletion of the equivalent two exons of *Uqcrh* that was generated by the International Mouse Phenotyping Consortium (IMPC; Fig 1C). A comparison of the deletion between the human and mouse protein is shown in Fig 1D. Notably, there is also a pseudogene (*UQCRHL*) in humans, which is absent in mice (Fig 1D).

**Figure 2:**
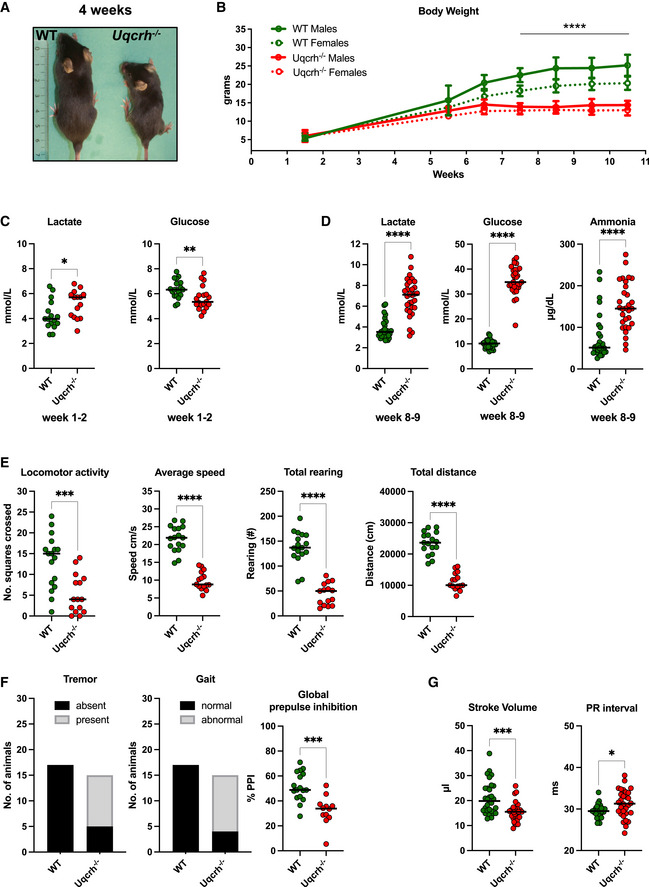
*Uqcrh* ^−/−^ mice displayed several essential characteristics of human CIII disorders ARepresentative picture of a WT and age‐ and sex‐matched *Uqcrh* ^−/−^ mouse at 4 weeks of age. Scale expressed in centimetres.BBody weight of *Uqcrh* ^−/−^ mice and wild‐type littermates at 1–2 weeks (*n* = 10 f, 5 m *Uqcrh* ^−/−^ and 8 f, 8 m WT); during the post‐weaning period from 5 to 6 weeks of age (*n* = 3 m, 7 f *Uqcrh* ^−/−^ and 3 f, 3 m WT); 6–7 weeks of age (*n* = 28 f, 23 m *Uqcrh* ^−/−^ and 22 f, 23 m WT); 7–8 weeks of age (*n* = 33 f, 30 m *Uqcrh* ^−/−^ and 45 f, 40 m WT); 8–9 weeks of age (*n* = 21 f, 25 m *Uqcrh* ^−/−^ and 35 f, 37 m WT); 9–10 weeks of age (*n* = 45 f, 45 m *Uqcrh* ^−/−^ and 61 f, 66 m WT); 10–11 weeks of age (*n* = 14 f, 13 m *Uqcrh* ^−/−^ and 21 f, 25 m WT). Data are shown as mean ± SD. ****P < 0.0001, 2‐way ANOVA, from 7.5 weeks of age for *Uqcrh* ^−/−^ males and females versus sex‐matched controls.C, DClinical chemistry plasma concentrations of parameters related to acid‐base balance (lactate and ammonia) and metabolic state (glucose) *n* = 15 *Uqcrh* ^−/−^ and 16 WT pups 1–2‐week‐old (C); *n* = 15 f, 15 m *Uqcrh* ^−/−^ and 15 f,15 m WT mice aged 8–9 weeks (D); sex not differentiated. Data are shown as scatter dot plots with a line at the median. *P < 0.05, **P < 0.01, ****P < 0.0001, Wilcoxon rank‐sum test.E, FMeasures related to behaviour and neurological function, data generated by the open‐field test (8 weeks old, E) and SHIRPA (9 weeks old, F), *n* = 7f, 8m *Uqcrh* ^−/−^ and 8f, 9m WT animals. Prepulse inhibition of the acoustic startle as a parameter associated with sensorimotor gating (10 weeks, E right panel), *n* = 7 f/5 m *Uqcrh* ^−/−^ and 8 f/ 9 m WT mice. Sex is not differentiated. Data are shown as scatter dot plots with a line representing the median. ***P < 0.001, ****P < 0.0001, Wilcoxon rank‐sum test.GParameters related to heart function generated by echocardiography (ECHO) and electrocardiography (ECG), *n* = 15 f, 15 m *Uqcrh* ^−/−^ and 15 f, 15 m WT mice, 6 weeks old. Data information: Data are shown as single data points and median, or categorical data, males and females pooled, since genotype‐related effects were comparable for both sexes from different cohorts. *P < 0.05, ***P < 0.001, Wilcoxon rank‐sum test.

While the human patients with the homozygous *UQCRH* two‐exon deletion showed recurrent episodes of metabolic crises, but developed normally and were otherwise asymptomatic in between these episodes, the homozygous mice harbouring the *Uqcrh* two‐exon deletion (hereafter *Uqcrh* ^−/−^ mice) showed a more severe phenotype. *Uqcrh* ^−/−^ mice were viable, but born at a lower Mendelian ratio and presented with failure to thrive after birth. Indeed, crossings of heterozygous mice resulted in 161 pups, of which 13.66% were homozygous mutants (41 *Uqcrh* ^+/+^, 98 *Uqcrh* ^+/−^, 22 *Uqcrh* ^−/−^, 78 total males, and 83 total females), and below the expected 25%, indicating an important function of *Uqcrh* during embryonic development. After weaning, *Uqcrh* ^−/−^ mice were smaller (Fig 2A) and gained less weight than the wild‐type mice up to 5–6 weeks of age, when their growth stopped and weight remained constant (Fig 2B). At 10 weeks *Uqcrh* ^−/−^ males and females presented with an average weight of 14.4 g (57% of wild‐type) and 13.0 g (64% of wild‐type), respectively (Fig 2B), while the wild‐type male and female mice were 25.2 g and 20.3 g, respectively. Despite the body weight difference of male and female *Uqcrh* ^−/−^ mice, phenotypic data were compiled for both sexes and were independent of sex. Metabolic investigations of 1–2‐week‐old *Uqcrh* ^−/−^ mice revealed increased blood lactate levels (median [q1; q3]: 5.700 [4.185; 5.910] mM in *Uqcrh* ^−/−^ vs. 3.970 [3.568; 4.775] mM in wild‐type mice) and slightly decreased blood glucose levels (median [q1; q3]: 5.353 [4.996; 5.911] mM in *Uqcrh* ^−/−^ vs. 6.328 [6.078; 6.966] mM in wild‐type mice; Fig 2C). This replicated the phenotype seen in the patients during episodes of metabolic crisis with lactic acidosis and hypoglycaemia. Blood lactate levels in *Uqcrh* ^−/−^ mice increased over time and at 8–9 weeks was approximately twice as high as in wild‐type littermates (median [q1; q3]: 7.110 [5.865; 8.018] mM in *Uqcrh* ^−/−^ vs. to 3.525 [3.138; 4.045] mM in wild‐type mice) (Fig 2D). In contrast to the episodes of hypoglycaemia seen in patients, blood glucose levels in *Uqcrh* ^−/−^ mice increased with age up to 3.5‐fold higher than wild‐type littermates when 8–9 weeks old (mean 35.41 mM vs. 10.00 mM) (Fig 2D). *Uqcrh* ^−/−^ mice also had increased ammonia levels (mean 151.09 mM vs. 74.48 μM in wild‐type mice), similar to the patients during episodes of metabolic crisis (Fig 2D).

**Figure EV2:**
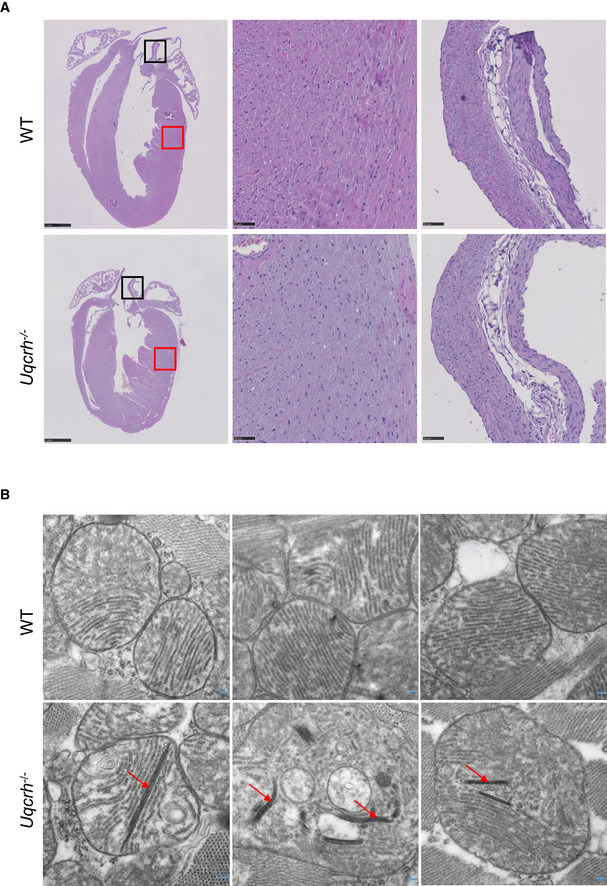
Histology of the heart (haematoxylin and eosin staining and electron microscopy) Representative photomicrographs of heart tissue, stained with haematoxylin and eosin, from wild‐type (WT, upper panel) and *Uqcrh* ^−/−^ (lower panel) mice at 9 weeks of age. Normal histopathological structure of the myocardium (red box, central panels) and aorta (black box, right panels) were observed in both groups of mice. Scale bars represent 1 mm in the left panels and 50 μm in the central and right panels.Electron microscopy images of 3 representative wild‐type (WT, upper panel) and 3 *Uqcrh* ^−/−^ mice (lower panel) heart mitochondria. Mitochondria from Uqcrh^−/−^ mouse heart tissue showed paracrystalline inclusions (arrows). Magnification 1:50,000.

Spontaneous locomotor activity, average speed, total rearing and the distance travelled in the open‐field arena were all diminished compared to wild‐type mice at 8 weeks of age (Fig 2E). In addition, 10 out of 15 *Uqcrh* ^−/−^ mice presented with tremor and 11 out of 15 *Uqcrh* ^−/−^ mice showed abnormal gait indicating motor dysfunctions (at 9 weeks of age) (Fig 2F). The global prepulse inhibition was significantly decreased at 10 weeks of age, suggesting a deficit in sensorimotor gating (Fig 2F right panel). *Uqcrh* ^−/−^ mice presented with the prolongation of the PR intervals in the electrocardiogram and a decrease of left ventricular stroke volume, observed as early as 6 weeks of age (Fig 2G) indicating impaired conduction through the atrioventricular node. A microscopic investigation of the heart tissue from *Uqcrh* ^−/−^ mice by H&E staining, however, did not reveal any alterations compared to wild‐type mice (Fig EV2A), but electron microscopy revealed mitochondrial paracrystalline inclusions in 5 of the 6 *Uqcrh* ^−/−^ hearts studied at 8 weeks of age (Fig EV2B). Finally, at the age of 8–12 weeks, *Uqcrh* ^−/−^ mice showed hypoproteinaemia, hypoalbuminemia, hyperkalaemia, increased activities of liver marker enzymes (ALAT, ASAT), hyperphosphatasaemia (increased ALP), decreased calculated total iron‐binding capacity (TIBC) and strong hyperglycaemia (Appendix Table S2). Taken together, this highlights a multiorgan decompensation with the failure of several organs to compensate for the functional overload resulting from the mitochondrial impairment. At the age of 12 weeks, *Uqcrh* ^−/−^ mice reached a humane endpoint and were sacrificed. No difference in response to isoflurane in *Uqcrh* ^−/−^ mice compared to controls was observed. An overview of the results is given in Table 1.

**Table 1:** Summary of relevant phenotypes observed in 7‐ to 12‐week‐old *Uqcrh* ^−/−^ compared to wild‐type WT mice.

Organ systems (Tests performed)	Phenotype summary *Uqcrh* ^−/−^ (mice > 7–12 weeks of age)
Nervous system	
Behaviour and Neurology (Open ield, SHIRPA, Pre‐pulse inhibition/Acoustic startle reflex, Grip strength)	Decreased spontaneous locomotor and exploratory activity; 67% of the mice which presented with tremor, 61% with abnormal gait; Reduced grip strength; Decreased acoustic startle response (possible effect on hearing but reduced body weight could contribute to this) and prepulse inhibition (sensorimotor gating)
Eye	
Optical Coherence Tomography (OCT)	Developmental phenotype
Metabolism	
Indirect calorimetry (TSE)	Reduced energy expenditure and metabolic flexibility; Impaired circadian rhythms
Body composition (NMR)	Increased fat mass and reduced lean mass in relation to reduced body weight
Blood	
Clinical Chemistry (AU, MSD)	Lactate and ammonia increased; bicarbonate normal; Glucose: Hypo to hyperglycaemia; Electrolytes: decreased Na and Cl, K increased; Liver enzymes: increased ALAT, ASAT, ALP; Urea increased, decreased total protein and albumin, higher iron levels; Lipids: normal cholesterol, lower lipase activity, lower triglycerides, especially in females; Higher iron levels, lower TIBC and UIBC
Haematology (AU)	Decreased white blood cell count; increased red blood cell count and haemoglobin; decreased platelet; leukocytes; thrombocytes; MCH and MCHC
Immunology (FACS, MSD)	T cells decreased, higher CD4 and lower CD8; B cells increased; NK cell decreased; Granulocytes and monocytes were normal; Macrophages increased; eosinophils decreased; high IgA; low IgG1; high IFNg, IL‐2 and lower IL1b in male mutant mice; lower IL‐4, IL‐5, IL12p70, TNFa KC‐GRO and higher IL‐10 in Uqcrh females
Pathological examination (macro‐ and microscopy, EM)	
Kidney	Tubular alterations in the kidney. Lesions begin at the age of 6 weeks and progress with age
Heart (Electron microscopy)	Paracrystalline inclusions in the mitochondria of the heart

### Patient fibroblasts and *Uqcrh* ^−/−^ mouse tissues show a CIII defect

**Figure 3:**
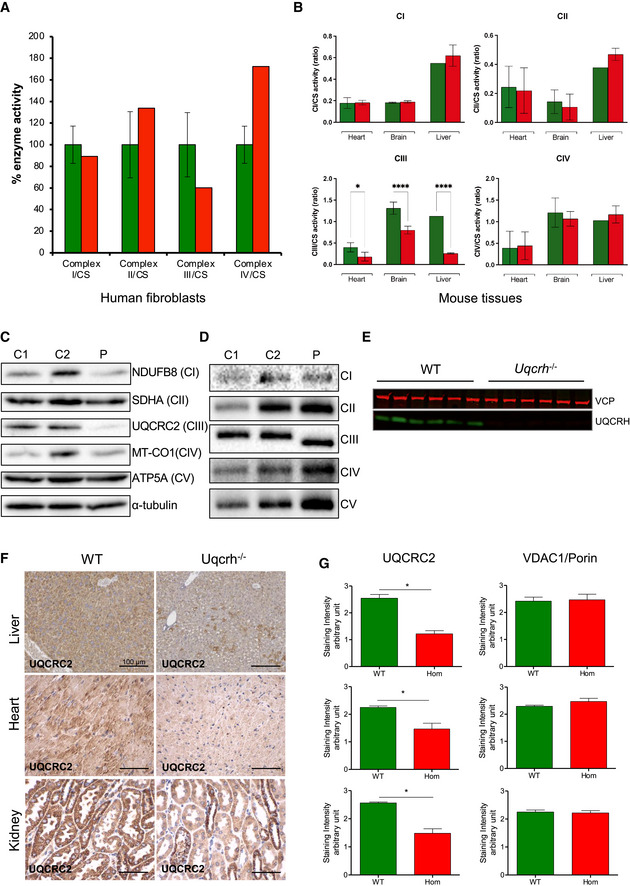
Biochemical assessment of patient fibroblasts and *Uqcrh* ^−/−^ mice shows impaired complex III activity and stability Respiratory chain enzyme activities from human control (green) and patient (red) fibroblasts. Mean activities of controls (*n* = 8) are set to 100% and error bars represent 1 standard deviation. Data are normalised to citrate synthase (CS) activity.Respiratory chain enzyme activities in heart, brain and liver, of wild‐type and *Uqcrh* ^−/−^ mice. Data are normalised to citrate synthase (CS) activity. Values are given as mean ± SD, *n* = 4 WT, 5 *Uqcrh* ^−/−^ (heart), 3 WT, 3 *Uqcrh* ^−/−^ (brain), 1 WT and 2 *Uqcrh* ^−/−^ (Liver). *P < 0.05, ****P < 0.0001, 2‐way ANOVA (multiple comparisons). Data showing CS activity/total protein concentration can be found in Appendix Fig S1A.Western blot analysis of OXPHOS components in the patient (P) and control (*n* = 2, C1‐2) fibroblasts.BN‐PAGE analysis of OXPHOS complex assembly in enriched mitochondria from patient and control fibroblasts (*n* = 2, C1‐2) solubilised with DDM. Immunoblotting was performed using antibodies to a subunit of each complex (CI [NDUFB8], CII [SDHA], CIII [UQCRC2], CIV [MT‐CO1] and CV [ATP5A]).Western blot analysis of liver lysates derived from 6 wild‐type (WT) and 6 *Uqcrh* ^−/−^ animals at 4 weeks of age. The upper band (97 kDa) refers to loading control VCP protein; the lower band (11 kDa) indicates UQCRH.Immunohistochemical staining of UQCRC2 was performed on liver, heart and kidney derived from wild‐type and *Uqcrh* ^−/−^ mice at 9 weeks of age. Staining of VDAC1/porin was also carried out (Appendix Fig S1B).Graphs indicate score values of immunohistochemical staining intensity of both VDAC1/porin and UQCRC2. Data are given as mean ± SEM. *P < 0.05; *n* = 3, Student’s *t*‐test (unpaired samples). Source data are available online for this figure.

To characterise the consequences of the identified two‐exon *UQCRH* deletion, we investigated the function of a primary dermal fibroblast cell line from the proband (II‐1) and multiple tissues from *Uqcrh* ^−/−^ mice. A sample of fibroblasts from the second individual II‐4 was not available. Biochemical analysis confirmed a specific decrease of CIII activity to approximately 60% compared to control cell lines (Fig 3A). Similarly, measurement of respiratory chain activities in the heart, brain and liver of *Uqcrh* ^−/−^ mice showed an approximate 50% decrease in the activity of CIII (Fig 3B, Appendix Fig S1A). This impairment of CIII activity, with similar residual activity, in both human and mouse tissue harbouring the two‐exon *UQCRH* deletion, demonstrates a functional consequence of the mutation and strongly suggests pathogenicity.

Western blot analysis of patient fibroblasts revealed a clear decrease in the steady‐state protein level of CIII subunit UQCRC2 (Fig 3C). This was used as a marker for CIII stability, as we were unable to detect UQCRH by western blotting in human fibroblasts, even in control samples. To assess the assembly of CIII, blue‐native gel electrophoresis (BN‐PAGE) analysis was carried out. In patient fibroblasts, it was shown that CIII does in fact assemble, consistent with the 60% residual activity of CIII, but with a slightly lower molecular weight (Fig 3D).

In protein extracts from mouse liver tissue, UQCRH could be detected directly in wild‐type samples but was absent in liver samples from *Uqcrh* ^−/−^ mice (Fig 3E). This strongly suggests that *Uqcrh* ^−/−^ mice do not express UQCRH, although the presence of a shortened version of the protein lacking exons 2 and 3 cannot be excluded. *Uqcrh* ^−/−^ mouse tissues also displayed a decreased staining for UQCRC2 (Fig 3F) when normalised to VDAC1/porin levels (Fig 3G, Appendix Fig S1B), which is consistent with the decreased expression of UQCRC2 seen in the patient (Fig 3C).

### Expression of wild‐type *UQCRH* in patient fibroblasts ameliorates the CIII defect

**Figure 4:**
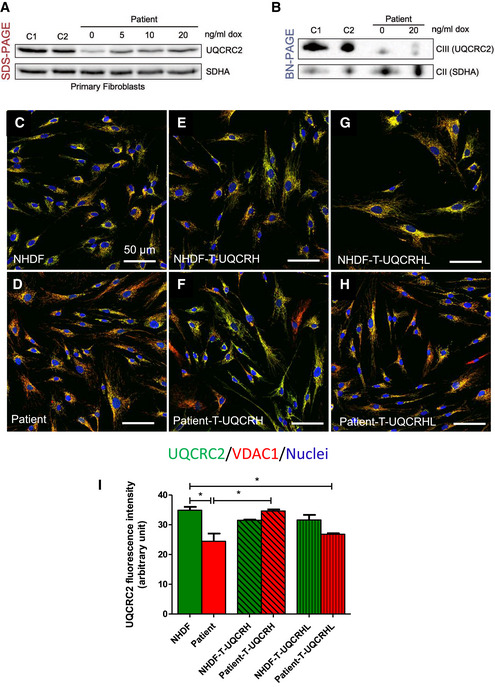
Lentiviral transduction of patient fibroblasts with wild‐type *UQCRH*, but not *UQCRHL* ameliorates the CIII defect Western blot analysis of control (*n* = 2, C1‐2) and patient fibroblasts transduced with a lentiviral vector (pLVX) containing wild‐type *UQCRH*. Expression of wild‐type *UQCRH* was induced using various concentrations of doxycycline (dox) up to 20 ng/ml for 72 h.BN‐PAGE analysis of control (*n* = 2, C1‐2) and patient fibroblasts transduced with a lentiviral vector (pLVX) containing wild‐type *UQCRH*. Transduced fibroblasts were either uninduced or induced with 20 ng/ml doxycycline (dox) for 72 h.Immunofluorescence for UQCRC2 (green), VDAC1/porin (red) and nuclei (DAPI, blue), in control (NHDF) fibroblasts.Immunofluorescence for UQCRC2 (green), VDAC1/porin (red) and nuclei (DAPI, blue), in patient fibroblasts.Immunofluorescence for UQCRC2 (green), VDAC1/porin (red) and nuclei (DAPI, blue), in control (NHDF) fibroblasts transduced with wild‐type *UQCRH*.Immunofluorescence for UQCRC2 (green), VDAC1/porin (red) and nuclei (DAPI, blue), in patient fibroblasts transduced with wild‐type (WT) *UQCRH*.Immunofluorescence for UQCRC2 (green), VDAC1/porin (red) and nuclei (DAPI, blue), in control (NHDF) fibroblasts transduced with the pseudogene *UQCRHL*.Immunofluorescence for UQCRC2 (green), VDAC1/porin (red) and nuclei (DAPI, blue), in patient fibroblasts transduced with the pseudogene *UQCRHL*.Graph indicating the staining intensity of UQCRC2 in control (NHDF) fibroblasts (green) and patient fibroblasts (red), those respective cell lines transduced (T) with wild‐type *UQCRH* (diagonal lines) and those cell lines transduced with the pseudogene *UQCRHL* (vertical lines) measured using *Image J*, Data are given as mean ± SEM. One‐way ANOVA (Kruskal‐Wallis test), *P < 0.05; *n* = 3 macroscopic field (10–14 cells for macroscopic field were measured).

To provide further evidence for the pathogenicity of the loss of *UQCRH*, we induced the expression of wild‐type *UQCRH* in patient fibroblasts in order to rescue the CIII deficiency. Patient fibroblasts were transduced with a wild‐type cDNA copy of *UQCRH* using a doxycycline‐inducible lentiviral vector at a low MOI (multiplicity of infection). Western blot analysis showed that doxycycline induction of wild‐type *UQCRH* increased the steady‐state protein levels of UQCRC2 in patient fibroblasts in a dose‐dependent manner (Fig 4A). However, UQCRC2 levels were not increased back to control levels even at higher concentrations of doxycycline, beyond 20 ng/ml. Similarly, when induced with doxycycline, BN‐PAGE analysis demonstrated an increase in fully assembled CIII of the correct molecular weight in the wild‐type transduced patient fibroblasts compared to the uninduced cells (Fig 4B), but again this did not reach control levels and the CIII assembly with the lower molecular weight was still present.

We then used a constitutively expressed construct at a higher MOI and performed immunohistochemistry analysis. As with the western blot analysis, UQCRC2 was used as a marker for CIII stability (Fig 4C) and was found decreased in patient fibroblasts (Fig 4D). Expression of wild‐type *UQCRH* did not affect UQCRC2 levels in the control cells (Fig 4E) but showed a significant UQCRC2 increase in the majority of transduced patient cells (Fig 4F). Unlike wild‐type *UQCRH*, expression of the pseudogene, *UQCRHL,* did not rescue the decreased UQCRC2 phenotype in patient fibroblasts (Fig 4G–I). Together these data demonstrate that expression of wild‐type *UQCRH* in patient fibroblasts ameliorates the CIII defect and thus confirms pathogenicity of the identified two‐exon deletion in *UQCRH*.

### BN‐PAGE analysis and complexome profiling of patient fibroblasts and *Uqcrh* ^−/−^ mouse tissues

To further understand the composition of the smaller assembly of CIII seen in patient fibroblasts and to assess any effects on supercomplex formation, we solubilised enriched mitochondria from human fibroblasts and mouse heart samples with the mild detergent digitonin and performed BN‐PAGE and complexome profiling as previously described (Giese *et al*, 2021).

**Figure 5:**
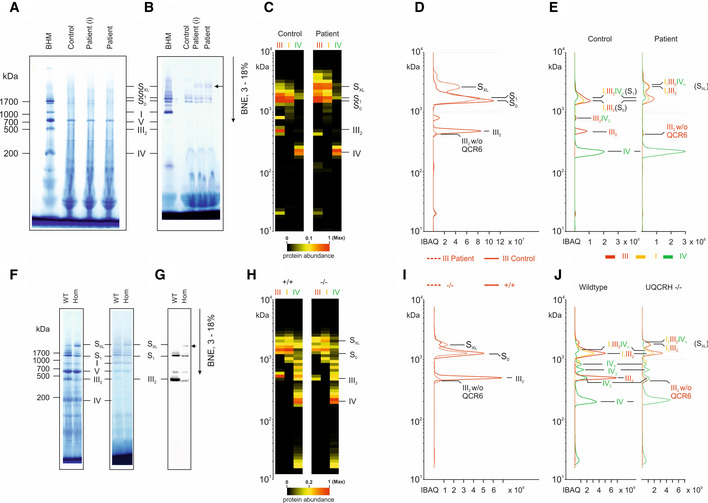
Two‐exon deletion in *UQCRH* alters supercomplex stoichiometries and stability Mitochondrial membranes from fibroblasts of control (WT) and patient fibroblasts (i, immortalised) (upper panels) and heart tissue from wild‐type (WT) and *Uqcrh* ^−/−^ mice (lower panels) were solubilised with digitonin and separated on native gradient gels.
BN‐PAGE gel stained with Coomassie showing molecular weight marker (BHM) and samples from control and patient (primary fibroblasts, (I) = immortalised fibroblasts).BN‐PAGE gel stained with NADH:NTB reductase activity stain showing molecular weight marker (BHM) and samples from control and patient (primary fibroblasts, (I) = immortalised fibroblasts).Further complexome analysis of the BN‐PAGE gels shown in A and B. Each native lane was cut into even fractions from high to low molecular mass. Proteins in each slice were digested with trypsin and analysed by quantitative mass spectrometry. Shown here are average IBAQ values of all identified subunits from complexes III, I and IV (full data including all individual subunits shown in Fig EV4).Profile of complex III (averaged IBAQ values) in the patient (dashed line) and control (solid line) fibroblasts.Profile of complexes III (red), I (yellow) and IV (green) to visualise and assess supercomplex composition and stability in control (solid lines) and patient (dashed lines) fibroblasts.BN‐PAGE gel stained with Coomassie (left) and NADH:NTB reductase activity stain (right) showing molecular weight marker (BHM) and heart tissue samples from wild‐type (WT) and *Uqcrh* ^−/−^ mice.BN‐PAGE and immunoblot using the antibody to CIII subunit UQCRC2 in heart tissue samples from wild‐type (WT) and *Uqcrh* ^−/−^ mice.Further complexome analysis of the BN‐PAGE gels shown in F, shown here are average IBAQ values of subunits from complexes III, I and IV (full data including all individual subunits shown in Fig EV4).Profile of complex III (averaged IBAQ values) in wild‐type (^+/+^, solid line) and *Uqcrh* ^−/−^ (^−/−^, dashed line) heart tissue.Profile of complexes III (red), I (yellow) and IV (green) to visualise and assess supercomplex composition and stability in wild‐type (^+/+^, solid line) and *Uqcrh* ^−/−^ (^−/−^, dashed line) heart tissue.

**Figure EV3:**
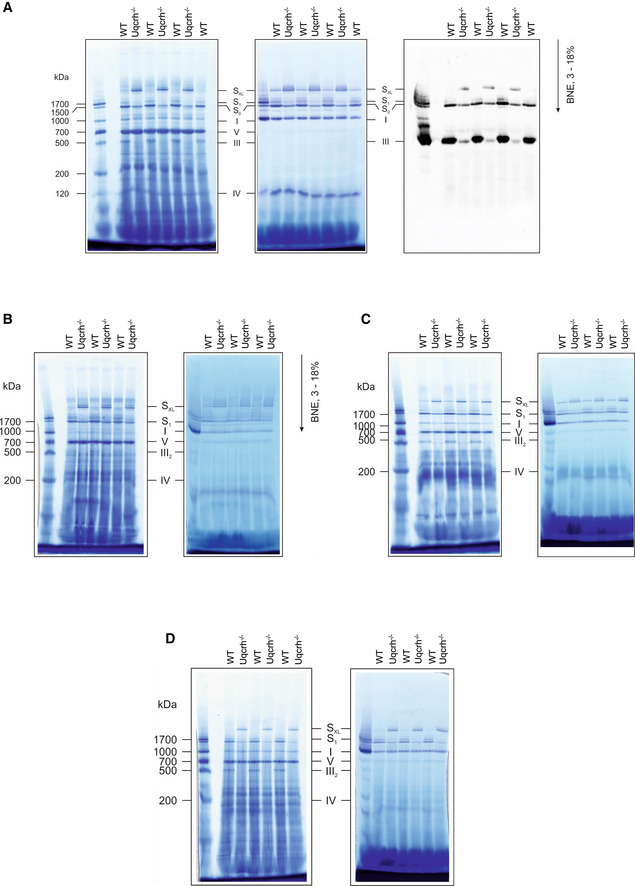
BN‐PAGE of multiple mouse tissues show increased staining for large supercomplex (S_XL_) in *Uqcrh* ^−/−^ mice Mitochondrial membranes from kidney samples of wild‐type (WT) and *Uqcrh* ^−/−^ mice were solubilised with digitonin and separated on native gradient gels. Protein complexes were stained with Coomassie (left panel) with NADH:NTB reductase activity stain (centre panel) and with an antibody against Core II subunit of complex III (Uqcrc2, right panel).Mitochondrial membranes from brain samples of wild‐type (WT) and *Uqcrh* ^−/−^ mice were solubilised with digitonin and separated on native gradient gels. Protein complexes were stained with Coomassie (left panel) with NADH:NTB reductase activity stain (right panel).Mitochondrial membranes from skeletal muscle samples of wild‐type (WT) and *Uqcrh* ^−/−^ mice were solubilised with digitonin and separated on native gradient gels. Protein complexes were stained with Coomassie (left panel) with NADH:NTB reductase activity stain (right panel).Mitochondrial membranes from liver samples of wild‐type (WT) and *Uqcrh* ^−/−^ mice were solubilised with digitonin and separated on native gradient gels. Protein complexes were stained with Coomassie (left panel) with NADH:NTB reductase activity stain (right panel). Data information: Assignment of complexes: I, complex I; III_2_, complex III dimer; IV, complex IV; S_0_, supercomplex containing complex I and a dimer of complex III; S_1_, supercomplex containing complex I, a dimer of complex III and 1 copy of complex IV, S_XL_, Supercomplex or Megacomplex containing I, III and higher signals also additional copies of IV.

Coomassie staining (Fig 5A) revealed the typical pattern of OXPHOS complexes in patient fibroblasts indicating that CIII can be assembled. It also revealed that CIII assembles with other respiratory complexes to form larger supercomplexes (Fig 5B). This is similar in tissues of *Uqcrh* ^−/−^ mice (Figs 5F and G, and EV3). As shown previously in patient fibroblasts (Fig 3D), CIII also appeared to assemble but migrated at a slightly lower MW in heart tissue from *Uqcrh* ^−/−^ mice (Fig 5G) as well as kidney (Fig EV3A). This change in migration pattern may reflect the lack of the UQCRH subunit. Another notable finding was an unexpected accumulation of a large supercomplex (S_XL_) in patients (Fig 5B, arrow) and in *Uqcrh* ^−/−^ mouse tissues (Figs 5F and G, and EV3) compared with the respective controls.

**Figure EV4:**
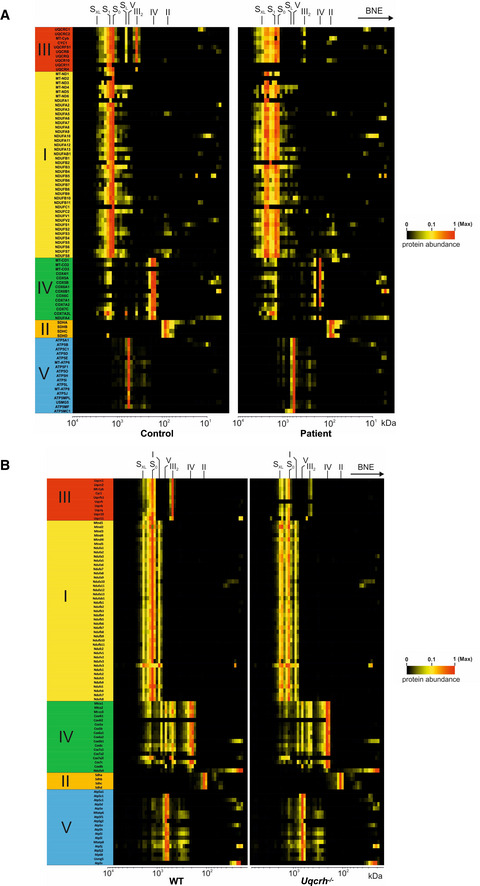
Complexome analysis of patient fibroblasts and *Uqcrh* ^−/−^ mouse heart tissue Mitochondrial membranes of control and patient fibroblasts were solubilised with digitonin and separated on native gradient gels. Each native lane was cut into even fractions from high to low molecular mass. Proteins in each slice were digested with trypsin and analysed by quantitative mass spectrometry.Mitochondrial membranes of heart from wild‐type and *Uqcrh* ^−/−^ deficient mice were solubilised with digitonin and separated on native gradient gels. Each native lane was cut into even fractions from high to low molecular mass. Proteins in each slice were digested with trypsin and analysed by quantitative mass spectrometry. Data information: All data were analysed together and complexome profiles were generated (see PRIDE repository PXD022856 and PXD022855). Assignment of complexes: I, complex I; III_2_, complex III dimer; IV, complex IV; S_0_, supercomplex containing complex I and a dimer of complex III; S_1_, supercomplex containing complex I, a dimer of complex III and 1 copy of complex IV, S_XL_, supercomplex or megacomplex containing I, III and higher signals also additional copies of IV.

**Figure EV5:**
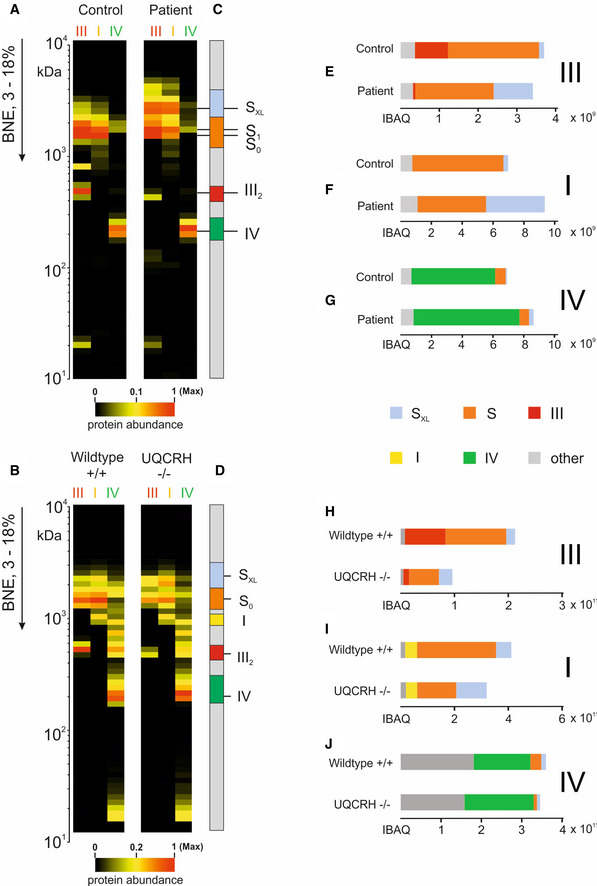
Alteration of the relative distribution of individual respiratory chain complexes and supercomplexes Mitochondrial membranes from fibroblasts of control (WT) and patient fibroblasts (upper panels, A–G) and heart tissue from wild‐type (WT) and Uqcrh^−/−^ mice (lower panels, B–J) were analysed by complexome profiling and the appearance of individual complexes and superassemblies were quantified.
A, BThe average of IBAQ values of all identified subunits from complexes III, I and IV (full data including all individual subunits shown in Fig EV4).C, DMolecular mass region used to quantify the appearance of individual and supercomplexes.E, HSum of all IBAQ values and relative distribution of complex III as individual dimer or assembled into supercomplexes and large supercomplexes.F, ISum of all IBAQ values and relative distribution of complex I as individual or assembled into supercomplexes and large supercomplexes.G, JSum of all IBAQ values and relative distribution of complex IV as individual or assembled into supercomplexes. Data information: Assignment of complexes: I, complex I; III2, complex III dimer; IV, complex IV; S0, supercomplex containing complex I and a dimer of complex III; S1, supercomplex containing complex I, a dimer of complex III and 1 copy of complex IV, SXL, Supercomplex or Megacomplex containing I, III and higher signals also additional copies of IV.

To gain deeper insights into the composition of OXPHOS complexes, we performed complexome profiling on human fibroblasts (patient and control) and mouse heart tissue (*Uqcrh* ^−/−^ and wild‐type). We confirmed the appearance of assembled CIII with a loss of UQCRH in both the human patients and *Uqcrh* ^−/−^ mice although they were of lower abundance compared with the respective controls (Figs 5C and H, EV4, and EV5). The amount of free complex IV was elevated in both the human patients (Fig EV5G) and *Uqcrh* ^−/−^ mice (Fig EV5J) possibly due to a compensatory effect of increased expression or due to there being less complex IV assembled into supercomplexes. Additionally, the relative abundance of supercomplexes in the mass range between 1.5 MDa and 2 MDa was lower compared with control mitochondria, suggesting that complex III lacking UQCRH assembles with the other respiratory complexes but to a lesser degree (Figs 5D, E, I, J and EV5). The distribution of free complex I was unchanged between controls and *Uqcrh* ^−/−^ mice in mitochondrial samples (Fig EV5I).

More importantly, we gained insights into the composition of supercomplexes, with the giant supercomplexes, S_XL_, consisting mainly of complexes I and III. From the apparent mass and composition of the subunits, we reveal that the most abundant giant supercomplex contains two CI and one dimer of CIII (Fig 5C–E, H–J). Only minor amounts contain CIV in this large assembly (Fig 5E and J). Moreover, we detected the very rare isoform Cox4i2 in *Uqcrh* ^−/−^ heart tissue at the level of individual CIV (Fig EV4B), suggesting a compensatory effect possibly similar to a response previously shown in hypoxic conditions (Fukuda *et al*, 2007).

## Discussion

Here, we report the first two cases of mitochondrial disease due to a homozygous variant in *UQCRH* that causes a two‐exon deletion (exons 2 and 3). The affected individuals are cousins and presented with recurrent episodes of metabolic decompensation, including severe lactic acidosis, hyperammonaemia, hypoglycaemia and encephalopathy. Notably, the patients were asymptomatic between these episodes. The identification of the *UQCRH* variant using WES and autozygosity mapping prompted functional studies on patient‐derived fibroblasts, which demonstrated a functional consequence of the identified *UQCRH* variant (Figs 3A, C, D and 4).

CIII deficiency is among the rarest of the isolated respiratory chain defects and is characterised by a broad range of symptoms typically involving tissues with high energy demands. Of the ten nuclear‐encoded structural subunits of CIII, only variants in *UQCRC2*, *UQCRB*, *UQCRQ* and *CYC1* have been reported in mitochondrial disease cases associated with hypoglycaemia, lactic acidosis, ketosis and hyperammonaemia (Haut *et al*, 2003; Barel *et al*, 2008; Gaignard *et al*, 2013; Miyake *et al*, 2013). These phenotypes and the episodic nature of the metabolic decompensation are extremely similar to the patients presented here.

We were unable to identify any additional patients with *UQCRH* variants, nor were there any previously described homozygous loss‐of‐function *UQCRH* variants in gnomAD (Sulem *et al*, 2015; Narasimhan *et al*, 2016). For this reason, we generated and characterised a mouse model harbouring the equivalent two‐exon deletion in murine *Uqcrh* (Figs 1C and D, and 2). Homozygous loss‐of‐function variants in CIII nuclear‐encoded structural proteins are not usually compatible with life and several previous knockouts have resulted in embryonic lethality (e.g. *Uqcrc1* ^−/−^, UQCRB mouse models) (Vempati *et al*, 2008; Wallace & Fan, 2009; Shan *et al*, 2019). In our model, homozygous *Uqcrh* knockout mice are viable and, importantly, recapitulate hallmarks of the human disorder (Appendix Table S3). It is worth noting that, although the *Uqcrh* ^−/−^ mice are viable, there was a reduced birth rate of the homozygotes (13%) compared with expected Mendelian ratios, thus suggesting that it is important but not fundamental to proper embryonic development.

Similar to the human patients during episodes of metabolic crisis, 1–2‐week‐old *Uqcrh* ^−/−^ mice displayed a metabolic phenotype with lactic acidosis and hypoglycaemia (Fig 2C). The key difference between the human patients and the *Uqcrh* ^−/−^ mice is that the mice did not follow an episodic pattern and the observed metabolic decompensation was progressive; thus, the *Uqcrh* ^−/−^ mice were more severely affected. Furthermore, the mouse phenotype switched towards severe hyperglycaemia, and at the age of 10–12 weeks, the general health of the *Uqcrh* ^−/−^ mice declined significantly. The reason for the more severe clinical phenotype of the *Uqcrh* ^−/−^ mice is unclear. One possibility is that the pseudogene *UQCRHL*, which is present in humans but not in mice, may be expressed and compensate somewhat for the deletion in *UQCRH*. This theory is not supported by our data, at least in patient fibroblasts, where lentiviral transduction and expression of *UQCRHL* did not significantly increase the low levels of UQCRC2 (Fig 4H and I), but transduction with wild‐type *UQCRH* did (Fig 4F and I).

Autosomal recessive CIII deficiency usually appears in infancy and impacts on major metabolic organs such as liver and kidney as well as heart, skeletal muscle and brain (McFarland & Turnbull, 2009; Ghezzi & Zeviani, 2018). Symptoms vary according to the severity of the OXPHOS impairment, ranging from mild muscle weakness, fatigue or exercise intolerance to systemic imbalance and multiorgan failure. In particular, they are usually characterised by hyperlactataemia and ketoacidosis with altered glycaemic values (Anastasio *et al*, 2017; Gaignard *et al*, 2017). The *Uqcrh* ^−/−^ model described here indeed presented with hyperkalaemia, hyponatraemia and hypochloraemia, which correlated with the impairment of heart function, weakness, fatigue and seizures.

The frequently observed encephalopathy in children with mitochondrial disease (Uziel *et al*, 2011; Chi, 2015) can have consequences on mental development and motor skills, which is a phenotype observed in the *Uqcrh* ^−/−^ mice (Fig 2E and F), despite unchanged brain structure. Conversely, our two patients have not shown signs of developmental impairment and attend public schools without required additional learning support. It will be interesting to observe whether the genetic defect affects their further development.

Notably, there is a broader spectrum of phenotypes associated with complex III defects in humans. Some present very similarly to the cases we describe, for example, patients with *UQCRC2* variants showed episodes of metabolic decompensation with lactic acidosis, hypoglycaemia, ketosis and hyperammonaemia without neurological impairment (Miyake *et al*, 2013).

Mutations in the *BCS1L* gene, encoding a CIII chaperone, are the most common cause of CIII deficiency and result in a variety of phenotypes, the most severe of which is GRACILE syndrome (growth restriction, aminoaciduria, cholestasis, iron overload, lactic acidosis and early death) highlighting the phenotypic heterogeneity in humans (Visapaa *et al*, 2002; Kotarsky *et al*, 2012; Kasapkara *et al*, 2014; Rajendran *et al*, 2019). A *Bcs1l* mutant mouse carrying the 232A>G variant has been previously generated and, very similarly to the *Uqcrh* ^−/−^ mouse, it developed normally but exhibited a failure to thrive from the weaning stage (Levéen *et al*, 2011; Tegelberg *et al*, 2017). Interestingly, there is some evidence that the genetic background of the *Bcs1l* mice affects the severity of the phenotype, with those from a C57BL/6JCrlBomTac having a short survival of 35 days (Levéen *et al*, 2011; Kotarsky *et al*, 2012; Rajendran *et al*, 2016; Purhonen *et al*, 2017), whereas in the slightly different C57BL/6JCrl background the homozygotes develop the same early manifestations but do not succumb to the early metabolic crisis, extending their survival to over 150 days (Purhonen *et al*, 2017; Tegelberg *et al*, 2017; Rajendran *et al*, 2019). It is possible that the severity of the disease may be altered in different genetic backgrounds, which may be an area for future study in *Uqcrh* ^−/−^ mice. Mice carrying the *Bcs1l* variant were born at expected Mendelian ratios whereas the *Uqcrh* ^−/−^ mice were born at a lower ratio than expected. Of note, *Bcs1l* mice displayed hypoglycaemia as a common symptom, usually observed in humans with CIII defects, including the *UQCRH* variants characterised here. By contrast, *Uqcrh* ^−/−^ mice first presented with slight hypoglycaemia in early life that switched to constant and massive hyperglycaemia later in life (Fig 2C and D), suggesting continuous anaerobic glycolysis with the transformation from glucose to lactate. Interestingly, there are reports on hyperglycaemia, resembling neonatal diabetes and pancreatic hypoplasia or agenesis, found in individuals with CIII disorders, showing that this can also be an observed phenotype in human CIII deficiency, reportedly due to an impairment of the endocrine function of the pancreas (Anastasio *et al*, 2017). Hypoglycaemia caused by glycogen storage/synthesis diseases, impaired gluconeogenesis or fatty acid oxidation is usually attributed to liver function, whereas hypoglycaemia due to insulin, GH or cortisol impairment is caused by endocrine dysfunction. In some respiratory chain defects, the impairment in glucose metabolism coincides with normal hepatic and endocrine functions. For example, a patient with CIII deficiency (pathogenic *UQCRB* variant) had occasional episodes of hypoglycaemia and lactic acidosis, rapidly corrected by glucose infusions (Mochel *et al*, 2005). Episodes were occurring due to prolonged fasts due to gastroenteritis and/or intercurrent infections. A deeper investigation revealed normal lipid oxidation but impaired gluconeogenesis due to the non‐use of alanine and lactate during prolonged fasting. Whether *UQCRH* deficiency involves gluconeogenesis or insulin signalling is an area for further investigation.

The contrast between episodes of hypoglycaemia observed in patients versus the hypoglycaemia developing gradually to constant hyperglycaemia in the *Uqcrh* ^−/−^ mice was among the most striking difference observed. While the patients were treated and recovered from their recurrent episodes of hypoglycaemia, *Uqcrh* ^−/−^ mice were not treated; therefore, normal glucose levels were not restored. Thus, hypoglycaemia appears to be the primary response to the OXPHOS deficiency. This is consistent with impaired ATP or cofactor (NAD and FAD) levels, which are usually observed in respiratory chain defects, impacting upon glucose homeostasis in several ways. We observed that, when not treated, the hypoglycaemic burden on the mice developed into persistent hyperglycaemia. Whether this is an adaptation mechanism of the mouse is yet unknown.

Despite the disparity in the severity of the clinical manifestations between the human patients and *Uqcrh* ^−/−^ mice, there was a striking similarity in the biochemical analysis of each. Firstly, both exhibit a decrease in CIII activity but retain significant residual activity. The decrease in the activity of CIII observed in *Uqcrh* ^−/−^ mice was approximately 50% (Fig 3B), which was comparable to the human patients (Fig 3A) and together suggests that UQCRH is not essential but important for optimal CIII activity. Similarly, a decrease in steady‐state levels of CIII subunit UQCRC2 was seen in patient fibroblasts by western blotting (Fig 3C) and immunohistochemistry (Fig 4D) and in *Uqcrh* ^−/−^ mice by immunohistochemistry (Fig 3F), commonly indicating instability of fully assembled CIII. There are examples of UQCRH levels correlating with other CIII subunits, such as UQCRB, UQCRC2 and CYC1 expression in hepatocellular carcinoma (Park *et al*, 2017). The impairment of CIII stability was further demonstrated with BN‐PAGE analysis showing not just a decreased abundance of assembled complex III, but also a shift in its size (Figs 3D, 4B and 5C [patient] and Figs 5G and EV3A [*Uqcrh* ^−/−^ mouse]). Complexome analysis confirmed that neither the patient nor the *Uqcrh* ^−/−^ mouse expressed the UQCRH (Uqcrh) protein (Fig EV4), confirming the absence of Uqcrh seen on western blot in *Uqcrh* ^−/−^ mouse liver tissue (Fig 3E). Furthermore, UQCRH/Uqcrh was the only CIII subunit that was not detected in assembled CIII in patient or *Uqcrh* ^−/−^ mouse samples but was detected in the respective controls (Fig EV4, EV5A and B). This suggests the ˜1.1% shift in migration of assembled CIII was solely due to the absence of UQCRH/Uqcrh, despite the MW of UQCRH (18.4 kDa as mature form in dimer) only equating to ˜0.6% of CIII (˜483 kDa as a dimer), perhaps due to a structural change in the complex.

Interestingly, the relative distribution of supercomplexes and individual CIII and CIV was altered (Fig EV5). Perhaps most striking is the increase in large supercomplex S_XL_ (Figs 5B–J and EV3) in both patient fibroblasts and *Uqcrh* ^−/−^ mouse tissues. Increased levels of S_XL_, in the patient and *Uqcrh* ^−/−^ mouse, strongly suggests a common aetiology thereby adding to the evidence for pathogenicity of the two‐exon deletion in *UQCRH*. From the apparent mass of ˜2.5 MDa, the composition of the subunits in our complexome profiling data (Fig EV4A) suggests that the most abundant giant supercomplex contains two CI connected via a dimer of CIII (Fig 5C–E, H–J), which is in accordance with a recently reported structure of human respiratory megacomplexes (Guo *et al*, 2017). We cannot rule out possible alternate stoichiometries in place of the second CI, although we could not identify any other mitochondrial complex of ˜1 MDa, nor any homo‐ or hetero‐multimer that would fit. Additionally, a larger complex of ˜2.9 MDa also included CI and CIII as well as identification of CIV, which likely represents the megacomplex of two CI, a CIII dimer and either one or two CIV assemblies (as also reported in Guo *et al*, 2017) (Fig 5E and J). Using this structure (Appendix Fig S2), we do not see that there is direct contact between UQCRH and complex I or complex IV (magnified in Appendix Fig S2C). Importantly, we observed the rare complex IV isoform, Cox4i2, in *Uqcrh* ^−/−^ heart tissue (Fig EV4B). In fact, only minor amounts of complex IV (< 1%) contain Cox4i2 in adult tissues under normal physiological conditions; however, its levels have been shown to increase during periods of hypoxia (Fukuda *et al*, 2007). Thus, it is possible that the induction of Cox4i2 may relate to a common compensatory mechanism.

The striking similarities in the biochemical analysis of the patient fibroblasts and *Uqcrh* ^−/−^ mouse tissue suggest that this *Uqcrh* ^−/−^ mouse model may represent a precious system to study human disease caused by CIII defects. There are, however, phenotypes in the *Uqcrh* ^−/−^ mice that are not seen in the patients harbouring the *UQCRH* mutation, such as altered contraction and pumping capacity of the heart (Fig 2G). Although cardiac abnormalities were not noted in the currently presented patients, respiratory chain complex disorders do associate with subclinical heart abnormalities in numerous infant patients, typically with poor prognosis (Yaplito‐Lee *et al*, 2007). Moreover, while not examined in the patients, the *Uqcrh* ^−/−^ mice showed paracrystalline structures (Fig EV2), which are often observed in mitochondrial myopathies (Pezeshkpour *et al*, 1991; Molnar & Schröder, 1998; Tarnopolsky *et al*, 2004; Bisceglia *et al*, 2014).

In summary, our data confirm the pathogenicity of the homozygous 2.2 kb *UQCRH* deletion by demonstrating an amelioration of the CIII defect in patient fibroblasts expressing wild‐type *UQCRH* and by demonstrating a strikingly similar biochemical phenotype between the patients and the mouse model harbouring the equivalent homozygous deletion of *Uqcrh*. Finally, the viable mouse model described here represents a valuable organism for the study of CIII biology and mitochondrial impairment more generally. Indeed, the *Uqcrh* ^−/−^ mouse holds great potential to be used for new therapeutic strategies targeting CIII defects or related mitochondrial disorders.

## Material and Methods

### Ethics statement

Informed consent was obtained for all patients and all experiments conformed to the principles set out in the WMA Declaration of Helsinki and the Department of Health and Human Services Belmont Report.

### Patient and control fibroblasts

The parents of the recruited children provided informed written consent for their participation in a study approved by the South Manchester Ethics committee (11/H1003/3, IRAS 64321) and the University of Manchester to determine the genetic cause of the condition affecting their children. Patient and normal human dermal fibroblasts (NHDF) (Lonza #CC‐2509) were cultured at 37°C and 5% CO_2_ in high glucose DMEM (Dulbecco’s modified Eagle’s medium, Gibco) supplemented with heat‐inactivated foetal bovine serum (Gibco), 200 µM uridine (Sigma‐Aldrich) and penicillin/streptomycin amphotericin B solution (Lonza). Cells were regularly tested for mycoplasma contamination; all cells used in the experiments were tested mycoplasma negative.

### Autozygosity mapping

Autozygosity mapping was undertaken on DNA samples from the proband (II:1), his two unaffected siblings (II:2 and II:3) and affected cousin (II‐4) by using the Genome‐Wide Human SNP Array 6.0 (Affymetrix, Santa Clara, CA, USA) as described previously (Daly & Thompson, 2010). Genotype calls were generated by the Birdseed V2 algorithm, and the results were analysed by AutoSNPa (Carr *et al*, 2006).

### Whole‐exome sequencing

Whole‐exome sequencing was undertaken on DNA extracted from lymphocytes from II‐1, using Agilent SureSelect v4 enrichment, followed by massively parallel sequencing on a HiSeq2000 (Illumina). Reads were aligned with the human reference genome version GRCh37/Hg19. Single‐nucleotide substitutions and small insertion and/or deletion variants were identified with a modified GATK variant‐calling pipeline. Filtering was undertaken to remove variants with a minor allele frequency of > 1% in the Exome Variant Server, Exome Aggregation Consortium (> 60,000 individuals), 1000 Genome databases or those seen previously within an in‐house dataset of over 600 individuals. Analysis was confined to the > 2 Mb regions of homozygosity defined by the autozygosity mapping.

### Uqcrh mouse model generation

*Uqcrh* knockout^(−/−)^ mice (C57BL/6NCrl‐Uqcrh^tm1b^(EUCOMM)Wtsi/Ieg) were generated by allele conversion of C57BL/6NCrl‐Uqcrh^tm1a^(EUCOMM)Wtsi/Ieg mouse line originating from EUCOMM ES clone EPD0378_3_C07. For further details on the construction of this clone, see the page at the IMPC portal (https://www.mousephenotype.org/data/alleles/MGI:1913826/tm1b(EUCOMM)Wtsi).

The tm1b allele was produced by deletion of exons two and three of *Uqcrh* and the neomycin cassette using a cell‐permeable Cre recombinase (Ryder *et al*, 2014). This allele is considered a true knockout as skipping over the LacZ cassette will not produce a functional protein. The cassette expresses LacZ under the control of the *Uqcrh* promoter as a fusion protein with exon one (Friedel *et al*, 2011).

The resulting mice were genotyped to verify mutation and the two‐exon deletion according to genotyping protocol, available on the Infrafrontier web page (https://www.infrafrontier.eu/sites/infrafrontier.eu/files/upload/public/pdf/genotype_protocols/EM10141_geno.pdf). Heterozygous mice were intercrossed to generate homozygous mutants.

### Housing conditions

Mice were maintained in IVC cages with water and standard mouse chow according to the directive 2010/63/EU, German laws and GMC housing conditions (www.mouseclinic.de). *Uqcrh* ^−/−^ homozygous mice and their wild‐type controls were fed *ad libitum* with moist food provided fresh twice a day in addition to the normal chow food. Food intake was monitored during daily health checks, and no issues were observed in food intake in the *Uqcrh* ^−/−^ mice.

### Mouse phenotyping

If not stated otherwise, all tests were performed following standard procedures as described before (Fuchs *et al*, 2011; www.mouseclinic.de) and approved by the responsible authority of the district government of Upper Bavaria.

Experimental groups were assigned according to the genotype of the animals. The selection of the mice for testing was balanced; control and mutants were measured alternately. Most of the tests were not conducted in blinded conditions because the results were recorded directly by the machines and, therefore, not influenceable by the examiner. The experiment was conducted in blinded conditions whenever there could have been an influence from the investigator. All the procedures are described in SOPs. Metadata for each data point was recorded throughout the measurements, and the influence of this metadata was monitored over time.

Bodyweight was measured for the different cohorts at different time points: at 1–2 weeks of age (*n* = 10 f, 5 m *Uqcrh* ^−/−^ and 8 f, 8 m WT); during the post‐weaning period from 5 to 6 weeks of age (*n* = 3 f, 7 m *Uqcrh* ^−/−^ and 3 f, 3 m WT); at 6–7 weeks of age (*n* = 28 f, 23 m *Uqcrh* ^−/−^ and 22 f, 23 m WT); 7–8 weeks of age (*n* = 33 f, 30 m *Uqcrh* ^−/−^ and 45 f, 40 m WT); 8–9 weeks of age (*n* = 21 f, 25 m *Uqcrh* ^−/−^ and 35 f, 37 m WT); 9–10 weeks of age (*n* = 45 f, 45 m *Uqcrh* ^−/−^ and 61 f, 66 m WT), and 10–11 weeks of age (*n* = 14 f, 13 m *Uqcrh* ^−/−^ and 21 f, 25 m WT). Animal number may contain more than one cohort and is always age and sex‐matched.

Data generated by the Open Field test and SHIRPA were obtained at 8 and 9 weeks of age (*n* = 7 female, 8 male *Uqcrh* ^−/−^ and 8 female, 9 male WT animals). The open‐field test evaluates the motor function and spontaneous exploration and was performed as previously described (Holter *et al*, 2015). A square arena (45 cm × 45 cm × 40 cm) surrounded by transparent plastic walls and a metal frame that is equipped with infrared beam detectors to automatically monitor the motor activity and location (centre or periphery) of the animal is explored by a mouse for 20 min at 200 Lux. Among the recorded parameters, total distance travelled and average speed, rearing (as exploratory behaviour) and time spent in the centre (as a measure of anxiety) are evaluated here. For neurological analysis, a modified SHIRPA protocol was applied (Fuchs *et al*, 2011) covering general neurobehavioral aspects which were rated with defined rating scales. For locomotor activity, the numbers of ground squares (100 cm^2^) the animals crossed in the first 30 s after transfer into the viewing arena were counted. During observation in the arena, a trained observer categorised gait (normal, abnormal) and recorded the occurrence of tremor during observation.

To evaluate sensorimotor gating via assessment of the acoustic startle reflex (ASR) and its prepulse inhibition (PPI), an ASR/PPI test was applied. It consists of a large sound‐attenuated chamber that isolates the animal in the presence of background noise (65 dB). A loudspeaker is located in the upper part of this chamber and the animal is enclosed in a transparent cylinder placed upon a piezoelectric motion sensor platform that transduces movements of the animal into electrical signals that are recorded and analysed. A session begins with an initial stimulus‐free acclimation period of 5 min (except for background noise), followed by 5 startle stimuli alone (110 dB) trials to measure ASR response. PPI was assessed for a startle stimulus level of 110 dB with prepulse levels of 67, 69, 73 and 81 dB preceding the startle pulse at an inter‐stimulus interval of 50 ms (Garrett *et al*, 2018). Prepulse inhibition was generated at 10 weeks of age (*n* = 7 female, 5 male *Uqcrh* ^−/−^ and 8 female, 9 male WT mice).

Parameters related to heart function generated by echocardiography (ECHO) and electrocardiography (ECG) were performed at 6‐week‐old mice (*n* = 15 female, 15 male *Uqcrh* ^−/−^ and 15 f, 15 male WT mice). High‐throughput electrocardiogram (ECG) and echocardiogram (ECHO) recordings in conscious mice were performed to assess the morphology and functionality of the heart (Moreth *et al*, 2014). Briefly, left ventricular function was evaluated with transthoracic echocardiography using a Vevo 2100 Imaging System (Visual Sonics). ECGs were recorded by ECGenie (Mouse Specifics Inc., Boston, MA) and analysed using e‐Mouse software (Mouse Specifics Inc.). The cardiac electrical activity was detected through three of the animals’ paws, and for each animal, intervals and amplitudes were evaluated from continuous recordings of at least 15 ECG signals.

Final blood samples were either collected from the retrobulbar vein plexus under isoflurane anaesthesia, by heart puncture after cervical dislocation, or collected from the trunk after decapitation (1–2‐week‐old pups) in Li‐heparin‐coated tubes (Li1000A, Kabe Labortechnik or Monovette 1.2 ml Sarstedt). Collected blood samples were centrifuged at 5000xg for 10 min at 8°C and plasma separated within one hour of blood collection. Clinical chemistry parameters were measured immediately using an AU480 analyser (Beckman‐Coulter) and adapted reagent kits from Beckman‐Coulter according to the manufacturer’s instructions, as described previously (Rathkolb, Fuchs, *et al*, 2013). Parameters determined included glucose (OSR 6121), lactate (OSR6193) and ammonia (OSR61154) for all samples and other standard clinical chemistry parameters, basic haematology and immunoglobulin levels for final samples collected from the first cohort. Immunoglobulins were determined using a mouse‐specific multiplex electroluminescence‐linked immune assay manufactured by mesoscale discovery (MSD), and haematological values were analysed with a Sysmex XT‐2000iV device using 1:5 diluted samples in the capillary mode as described (Rathkolb, Hans, *et al*, 2013). Clinical chemistry plasma concentrations of parameters related to metabolic state and acid‐base balance were measured 1–2 weeks old pups, sex not differentiated (*n* = 22 *Uqcrh* ^−/−^ and 22 WT mice) and mice aged 8‐9 weeks (*n* = 15 female, 15 male *Uqcrh* ^−/−^ and 15 female, 15 male WT mice). Animal number may contain more than one cohort and always age and sex‐matched.

### Mitochondrial respiratory chain activities

The protein extraction for citrate synthase (CS) and OXPHOS enzyme activity measurements were performed as previously described (Feichtinger *et al*, 2010, 2011). Briefly, heart tissues (60–100 mg) and whole brain were homogenised with a tissue disintegrator (Ultraturrax, IKA, Staufen, Germany) in extraction buffer (20 mM Tris–HCl, pH 7.6, 250 mM sucrose, 40 mM KCl, 2 mM EGTA). Successively, the samples were homogenised with a motor‐driven Teflon‐glass homogeniser (Potter S, Braun, Melsungen, Germany). The homogenate was finally centrifuged at 600 *g* for 10 min at 4°C. The supernatant containing the mitochondrial fraction was then used for the measurement of the enzyme activities. Spectrophotometric enzymatic measurements of CI‐IV and citrate synthase were performed at 37°C, as previously described (Berger *et al*, 2003; Meierhofer *et al*, 2004; Feichtinger *et al*, 2010).

### SDS–PAGE, DDM‐solubilised BN‐PAGE and Immunoblotting

For patient fibroblasts, steady‐state protein levels of OXPHOS components were analysed by SDS–PAGE (SDS–polyacrylamide gel electrophoresis) and OXPHOS complexes were assessed using DDM‐solubilised enriched mitochondria samples as previously described (Oláhová *et al*, 2015). Immunoblotting was carried out using primary antibodies against various OXPHOS subunits all used at a dilution of 1 in 1,000: mouse monoclonal NDUFB8 [Abcam ab110242], mouse monoclonal SDHA [Abcam ab14715], mouse monoclonal UQCRC2 [Abcam ab14745], mouse monoclonal MT‐CO1 [Abcam ab14705] and mouse monoclonal ATP5A [Abcam ab14748]) and loading controls at 1 in 10,000 dilutions, mouse monoclonal Porin/VDAC1 [Abcam ab14734] and mouse monoclonal Alpha‐Tubulin [Abcam ab7291]) followed anti‐mouse HRP‐conjugated secondary antibodies (Dako: P0260 1 in 2,000 dilution).

Proteins from mouse livers were extracted by tissue disruption in ice‐cold RIPA lysis and extraction buffers (Thermo Fisher Scientific) supplemented with 1x cOmplete^®^ Mini Protease Inhibitor Cocktail (Roche) and PhosSTOP™ (Roche). Protein concentrations were obtained using Pierce BCA Protein Assay Kit (Thermo Fisher Scientific) according to the manufacturer’s instructions. 20 µg of protein sample was loaded onto a 10% SDS–polyacrylamide gel (Bio‐Rad), and electrophoresis was performed. The lysate was subsequently transferred to a nitrocellulose membrane (Thermo Fisher Scientific). The membrane was blocked with Odyssey^®^ Blocking Buffer (TBS) (LI‐COR), thereafter incubated overnight at 4°C in primary antibody and subsequently in secondary antibody for 45 min at room temperature in the same buffer. As primary antibodies, rabbit polyclonal UQCRH (1:1,000 Abcam ab134949) and mouse monoclonal VCP (1:5,000, Abcam ab11433) for loading control were used. As secondary antibodies, IRDye 800CW anti‐rabbit (Gt) (1:10,000, LI‐COR, 926‐32211) and IRDye 680CW anti‐mouse (Gt) (1:10,000, LI‐COR, 926‐68070) were utilised. For fluorescent detection of proteins, Odyssey Infrared Imaging System and Odyssey^®^ software (LI‐COR) was utilised.

### Digitonin‐solubilised BN‐PAGE and complexome profiling

Sample preparation and blue‐native electrophoresis (BNE) of mouse tissue and human cells were carried out as described previously (Wittig *et al*, 2006). In‐gel activity stains and corresponding Western blots were performed as described previously (Wittig *et al*, 2007). A detailed description of complexome profiling was deposited together with mass spectrometry raw data, search results and data analysis files to the ProteomeXchange Consortium via the PRIDE partner repository with the dataset identifier PXD022855 (for mouse heart tissue) and PXD022856 (for patient fibroblasts) (Perez‐Riverol *et al*, 2019).

### Lentiviral gene rescue and cell culture

Wild‐type *UQCRH* was expressed in patient fibroblasts using the Lenti‐X TetOne Inducible Expression System. Wild‐type *UQCRH* insert was generated by PCR using cDNA from control fibroblasts using the following primers containing restriction sites for *EcoRI* and *AgeI* respectively: forward 5’‐CCCTCGTAAAGAATTCATGGGACTGGAGGACGAGCAAAAG‐3’, reverse: 5’‐ATCCGCCGGCACCGGTTTATTTCAAGTTGTTAAAGAGTTTGTGGGC‐3’. The insert was cloned into the linearised pLVX‐TetOne‐Puro plasmid vector (Takara Bio/Clontech) using the In‐Fusion HD Cloning Kit (Takara Bio/Clontech). The *UQCRH* containing vector was packaged into infectious lentiviral particles using Lenti‐X Packaging Single Shots (Takara Bio/Clontech) to transfect HEK293T according to the manufacturer’s guidelines. Media containing infectious lentivirus was harvested after 48 h and centrifuged at 500 *g* for 10 min to remove cellular debris and the supernatant was used to transduce patient fibroblasts at a ratio of 1:3 with fresh media and 4 mg/ml polybrene overnight. The media was replaced and puromycin (2 µg/ml) added to select for successfully transduced cells. Transduced cells were cultured in puromycin for 7 days before being maintained in standard high glucose. The expression of wild‐type *UQCRH* was induced by adding doxycycline (Takara Bio/Clontech; 100–200 µg/ml) to the media and incubating for 72 h.

For constitutive expression, *UQCRH* was cloned into pLenti6.3/V5‐TOPO^®^ TA Cloning^®^ Kit (Invitrogen) for the expression of the gene in human control dermal fibroblast (Lonza #CC‐2509) and patient fibroblasts. Generation of transgenic fibroblast cell lines was performed following a previously described transfection protocol (Kremer & Prokisch, 2017). Normal human dermal fibroblasts (NHDF) and patient’s fibroblasts were cultured as normal transduced fibroblasts media was supplemented also with 5 μg/ml Blasticidin S HCl (Thermo Fisher Scientific).

### Immunohistochemistry

For immunohistochemistry on mouse organs, liver, heart and brain were collected from four *Uqcrh* ^−/−^ and four wild‐type mice (two females and two males per group) at 9 weeks of age at the time of necropsy. The tissues were fixed in formalin for 24 h and embedded in paraffin for histological and immunohistochemical analysis. Immunohistochemistry was performed on 2 µm‐tick sections in an automated immunostainer (Discovery^®^XT, Roche, Penzberg, Germany). Briefly, sections were incubated with primary antibodies against UQCRC2 (1:1,000; Abcam) and VDAC1/porin (1:2,000; Abcam) with a streptavidin–peroxidase detection reagent. As a negative control, the primary antibody was omitted. Digital images were captured with the NanoZoomer^®^ 2.0HT (Hamamatsu, Japan) digital slide scanner. A scoring system was used to quantify the expression levels of UQCRC2 and VDAC1/Porin: 0 = no staining; 1 = weak staining; 2 = moderate staining; 3 = strong staining. The score for each section was calculated as the mean of 4 high‐power fields (Vidali *et al*, 2017).

### Immunofluorescence of chamber slides

Immunohistochemical staining was performed as previously described (Feichtinger, Neureiter, *et al*, 2017). Briefly, control and patient fibroblasts (transfected and non‐transfected) were grown on coverslips for 24 h. Afterwards, media was removed, and cells were fixated for 24 h in 4% formalin. Samples were then incubated in antigen retrieval buffer for 40 min at 95°C. Slides were incubated for 1 h at room temperature with the primary antibodies mouse monoclonal anti‐complex III subunit core 2 (1:400; Abcam, UK) and rabbit polyclonal anti‐voltage‐dependent anion channel VDAC1/porin (1:400; Abcam, UK) diluted in Dako antibody diluent with background reducing components (Dako, Vienna, Austria). Slides were incubated with the secondary antibodies Alexa‐Fluor‐594‐conjugated donkey anti‐rabbit IgG or Alexa‐Fluor‐488‐conjugated donkey anti‐mouse (1:1,000, 1 h, room temperature, Invitrogen, Vienna, Austria) diluted in PBS‐T. Coverslips were incubated for 10 min in a DAPI solution in PBS‐T. Finally, coverslips were washed in ddH_2_O and mounted with s fluorescent mounting medium (Dako, Vienna, Austria). Microscopy was carried out with a Zeiss LSM 880 confocal microscope. Fluorescence intensity was analysed with the help of ImageJ (National Institutes of Health, Bethesda, Maryland, USA). The intensity was measured in 10‐14 cells for each microscopic field for a total of three macroscopic fields for each type of cell. Both immunohistochemical and immunofluorescence analyses were performed in blinded conditions.

### Electron microscopy

Anaesthetised animals were perfused with 1.5% glutaraldehyde and 1.5% formaldehyde in 0.15 M HEPES (pH 8.0), and dissected hearts were stored in the same fixative at 4°C for at least 24 h. For epoxy resin embedding, heart samples were post‐fixed in 1% osmium tetroxide, stained *en bloc* in half‐saturated watery uranyl acetate, dehydrated in ascending grades of ethanol and embedded in Agar 100 (Agar Scientific). Ultrathin sections were cut using an ultramicrotome (Reichert Ultracut E, Leica) and examined in a transmission electron microscope (Zeiss EM 902). Digital images were captured with a slow‐scan 2K CCD camera (TRS, Tröndle, Moorenweis, Germany).

### Statistics

The selected sample size per sex and genotype was sufficient to find a medium difference of one standard deviation with a power of 0.8 and an alpha of 0.05. If not stated otherwise, data that were generated by the German Mouse Clinic were analysed using R (version 3.4). For each parameter, we calculate measures like mean, SD, effect size and, respectively, median and IQR in our standardised automatic R‐scripts. In general, no data point is excluded from the analysis unless there is clear evidence of, for example, technical failure of the experimental machine. For many of the parameters, there exist pre‐established threshold values as indicators for invalid measurements, these data points are replaced by status codes. Tests for genotype effects were made by using *t*‐test, Wilcoxon rank‐sum test, linear models, or two‐way ANOVA and with Tukey HSD post hoc tests, or Fisher’s exact test depending on the assumed distribution of the parameter and the questions addressed to the data (Table 1). Wild‐type mice data have been analysed in the GMC for more than 15 years, and the distribution of every single parameter is known. Based on that, the best statistical test was selected. Data are shown as single data points and median, or categorical data. Males and females were pooled if genotype‐related effects were comparable for both sexes. A *P* < 0.05 has been used as a level of significance; a correction for multiple testing has not been performed. Figures were prepared using GraphPad Prism version 7.00 for Windows (GraphPad Software, La Jolla, California, USA).

For immunohistochemistry and enzymatic activity, statistical analyses were performed using Prism 6 and 9 (GraphPad Software, USA). Results are given as mean ± SEM. Student’s t‐test (unpaired samples) was applied to compare two groups, one‐way ANOVA (Kruskal‐Wallis test), to compare more than one group and correct for multiple comparisons.

## Author contributions

HP, VG‐D, KT, RWT, WGN, MHA conceived and initiated the study; HP, VG‐D, HF, KT, RWT, WGN coordinated the study; JEU, KT, JAA‐P, OVA, LB, PS‐B, JC‐W, Y‐LC, LG, RG, KPH SMH, TK‐R, PM‐K, BR, JR, NS, IT, NFC, SV, RGF, JM, IW planned and performed the experiments; JAA‐P, OVA, LB, PS‐B, JC‐W, Y‐LC, LG, RG, SMH, TK‐R, PM‐K, BR, JR, NS, IT, NFC, MAO, GM, SV, RGF, JAM, JEU, KT, WGN, IW analysed the data; UG performed electron microscopy imaging; SM coordinated mouse line generation; CB, EJ, JHW, CSa clinically assessed the affected individuals and obtained patient samples; KG, SL, CSt, KP were involved in project management; RG, VG‐D, JAA‐P, BR, SL, KT, RWT, WGN, MS, SV drafted and worked at the manuscript; MHA, HF, VG‐D, WW provided oversight and resources; all authors read and approved the manuscript.

## Conflict of interest

The authors declare that they have no conflict of interest.

## For more information

1. OMIM: https://www.omim.org/entry/613844?search=UQCRH&highlight=uqcrh#title
2. gnomAD: https://gnomad.broadinstitute.org
3. Authors’ websites: https://www.newcastle‐mitochondria.com; https://www.mouseclinic.de/
4. Rare diseases resource links: https://rarediseases.info.nih.gov/diseases/8295/mitochondrial‐complex‐iii‐deficiency; https://www.infrafrontier.eu/infrafrontier‐and‐rare‐diseases
5. International Mouse Phenotyping Consortium (IMPC): https://www.mousephenotype.org/blog/2021/02/27/rare‐disease‐day‐2021/
6. Human&Mouse Genetic Resource: http://marrvel.org/

## Supporting information



Appendix

Expanded View Figures PDF

Source Data for Figure 3

## Data Availability

The datasets produced in this study are available in the following database: Complexome profiling data: ProteomeXchange consortium via the PRIDE repository, dataset identifiers PXD022856 (patient fibroblasts) and PXD022855 (mouse heart tissue). **PXD022856**: Project Webpage: http://www.ebi.ac.uk/pride/archive/projects/PXD022856 FTP Download: ftp://ftp.pride.ebi.ac.uk/pride/data/archive/2021/09/PXD022856 **PXD022855**: Project Webpage: https://www.ebi.ac.uk/pride/archive/projects/PXD022855 FTP Download: ftp://ftp.pride.ebi.ac.uk/pride/data/archive/2021/09/PXD022855
