# Supplementary material for: Characterising a homozygous two‐exon deletion in UQCRH: comparing human and mouse phenotypes
Source: EMBO Mol Med. 2021 Nov 8;13(12):e14397. doi: 10.15252/emmm.202114397 (PMC8649870; doi:10.15252/emmm.202114397)
Supplement: Supplementary file 1 — Appendix [file EMMM-13-e14397-s003.docx]

Table of Contents

[Appendix Fig S1 – Citrate synthase activity and immunostaining of VDAC1/porin 1](#_Toc80715582)

[Appendix Fig S2 - Human megacomplex structure 2](#_Toc80715583)

[Appendix Table S1 - Comprehensive clinical history of patients 4](#_Toc80715584)

[Appendix Table S2 - Clinical chemistry parameters measured in plasma 5](#_Toc80715585)

[Appendix Table S3 – Patient versus mouse comparison 6](#_Toc80715586)

## Appendix Fig S1 – Citrate synthase activity and immunostaining of VDAC1/porin

**B**

**A**


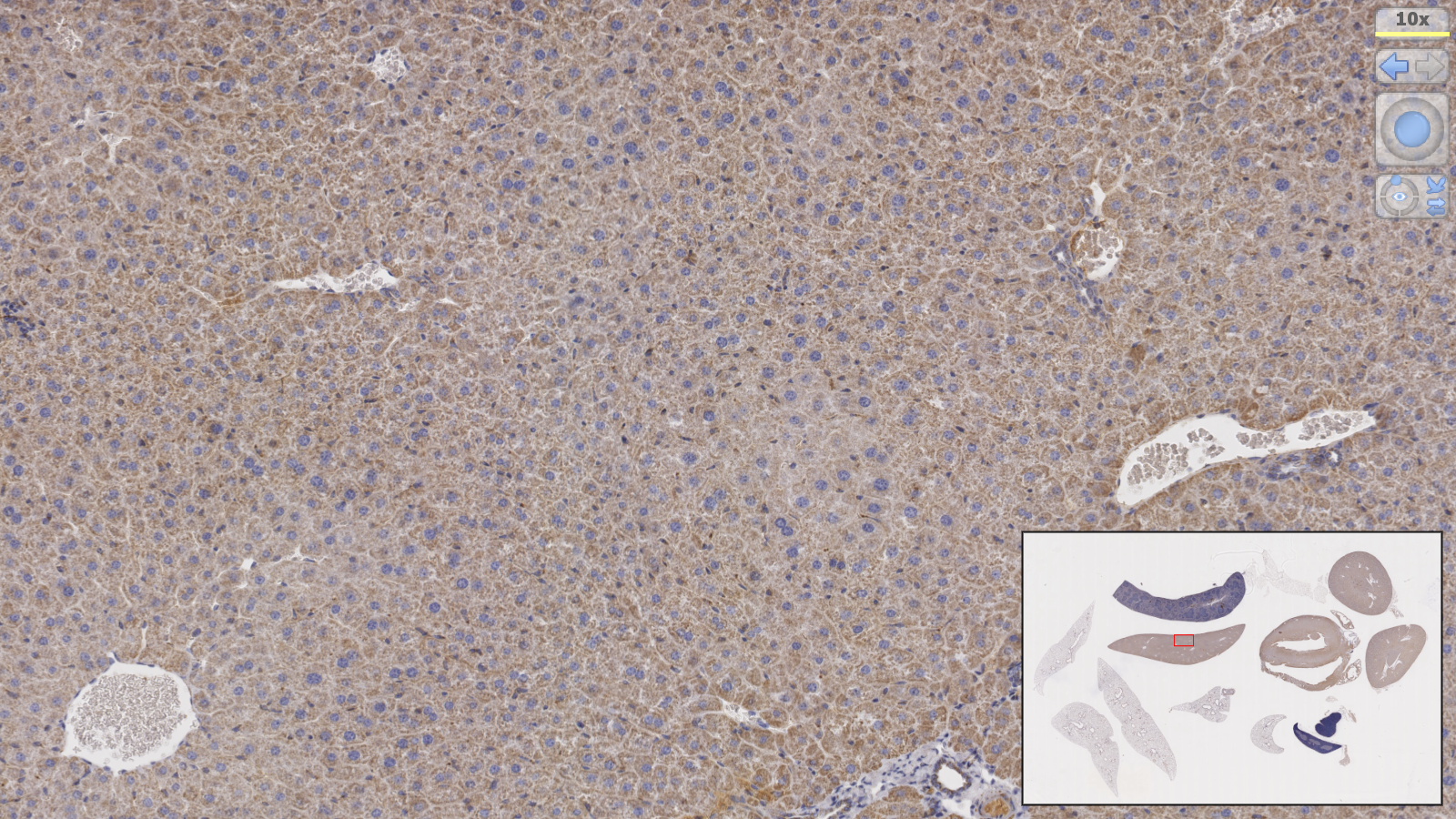


100 µm


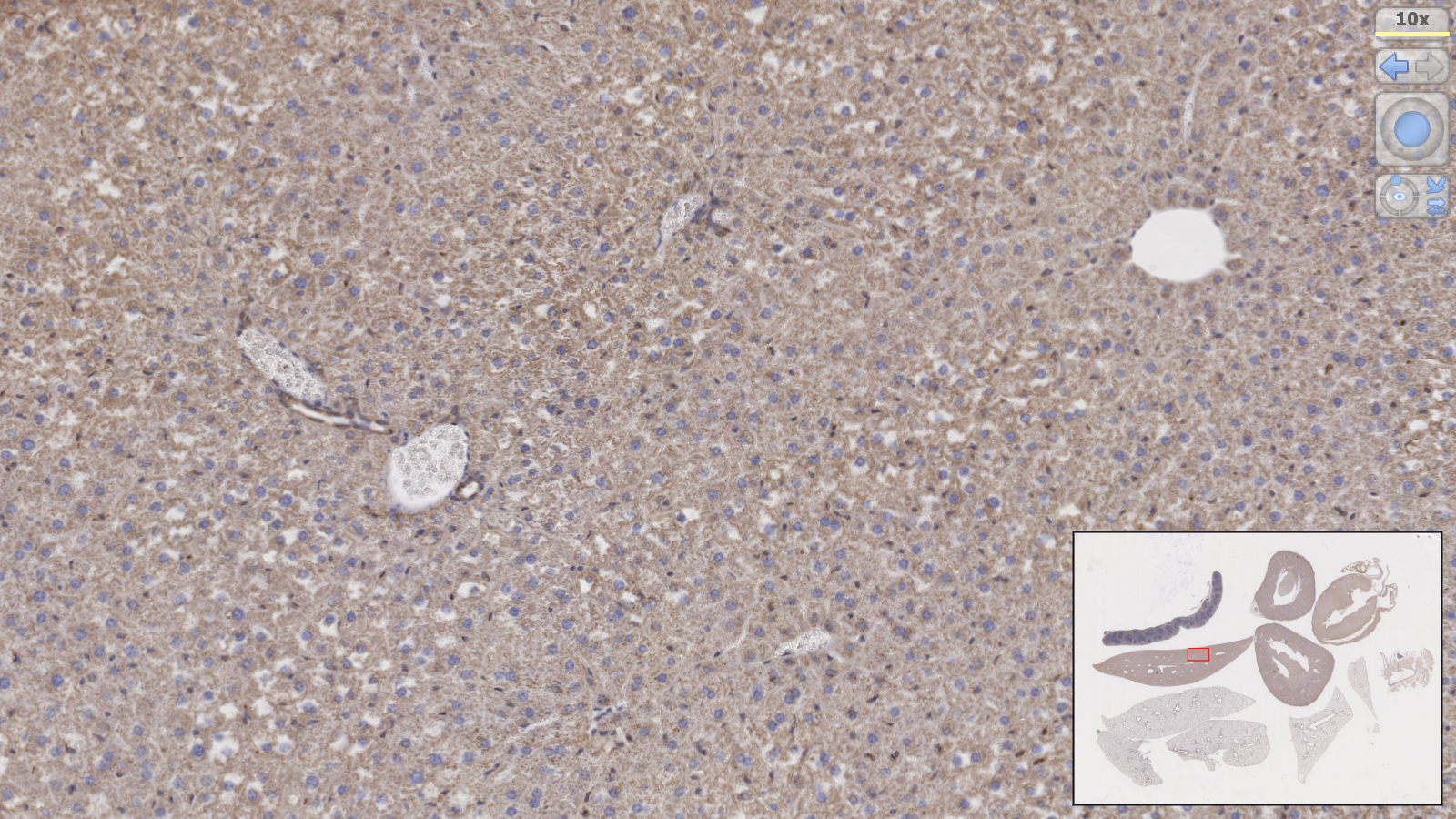

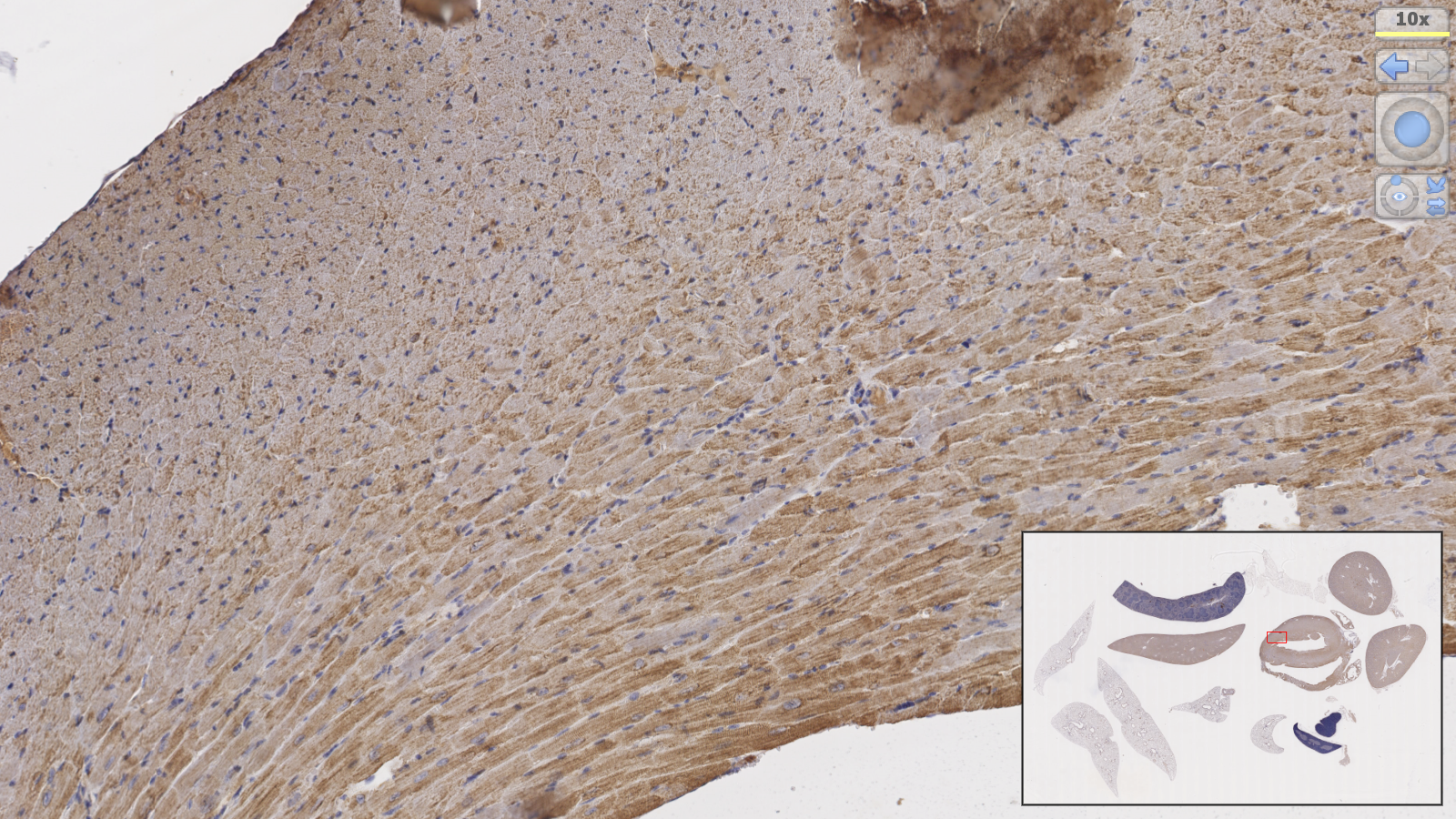

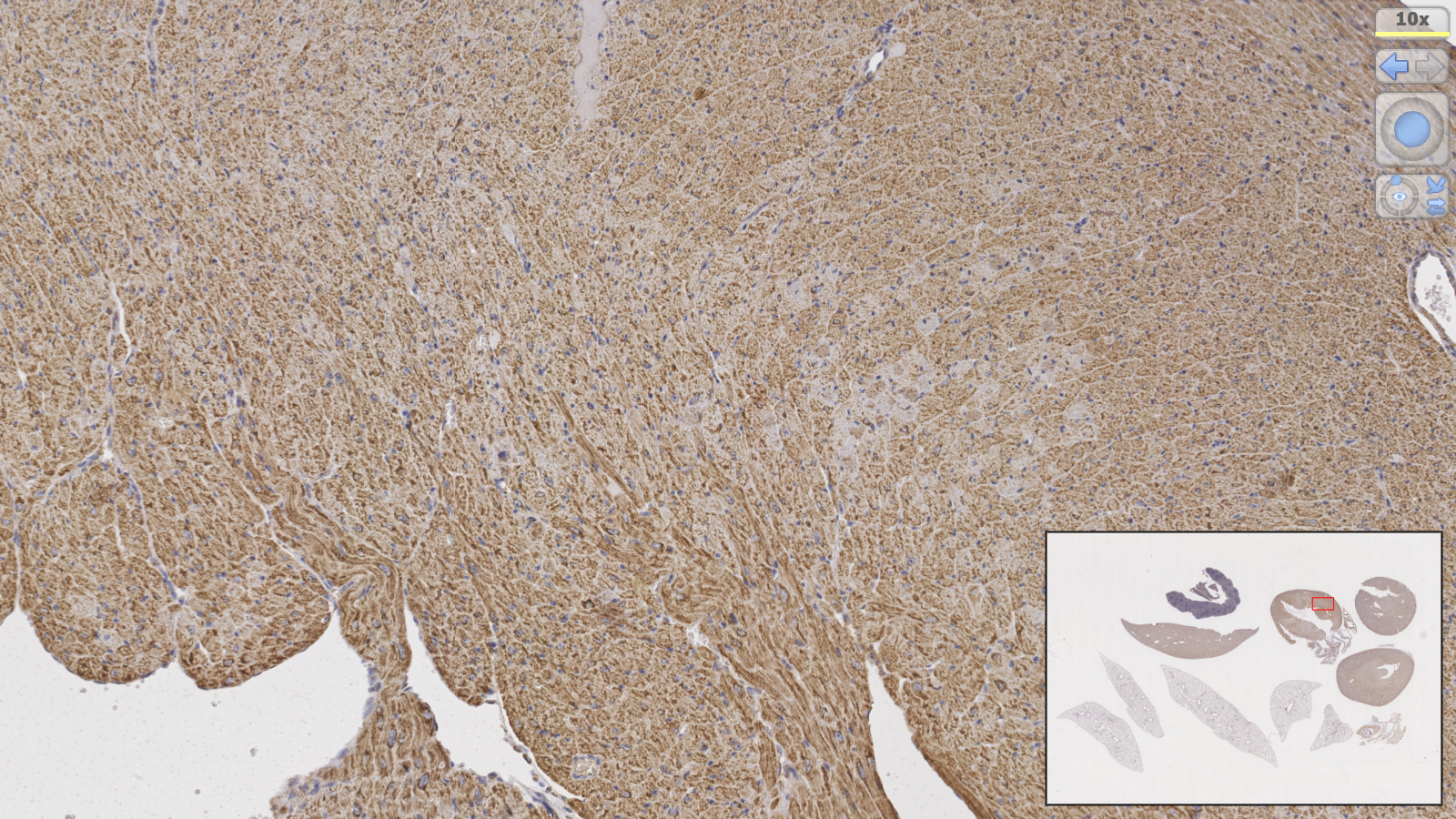

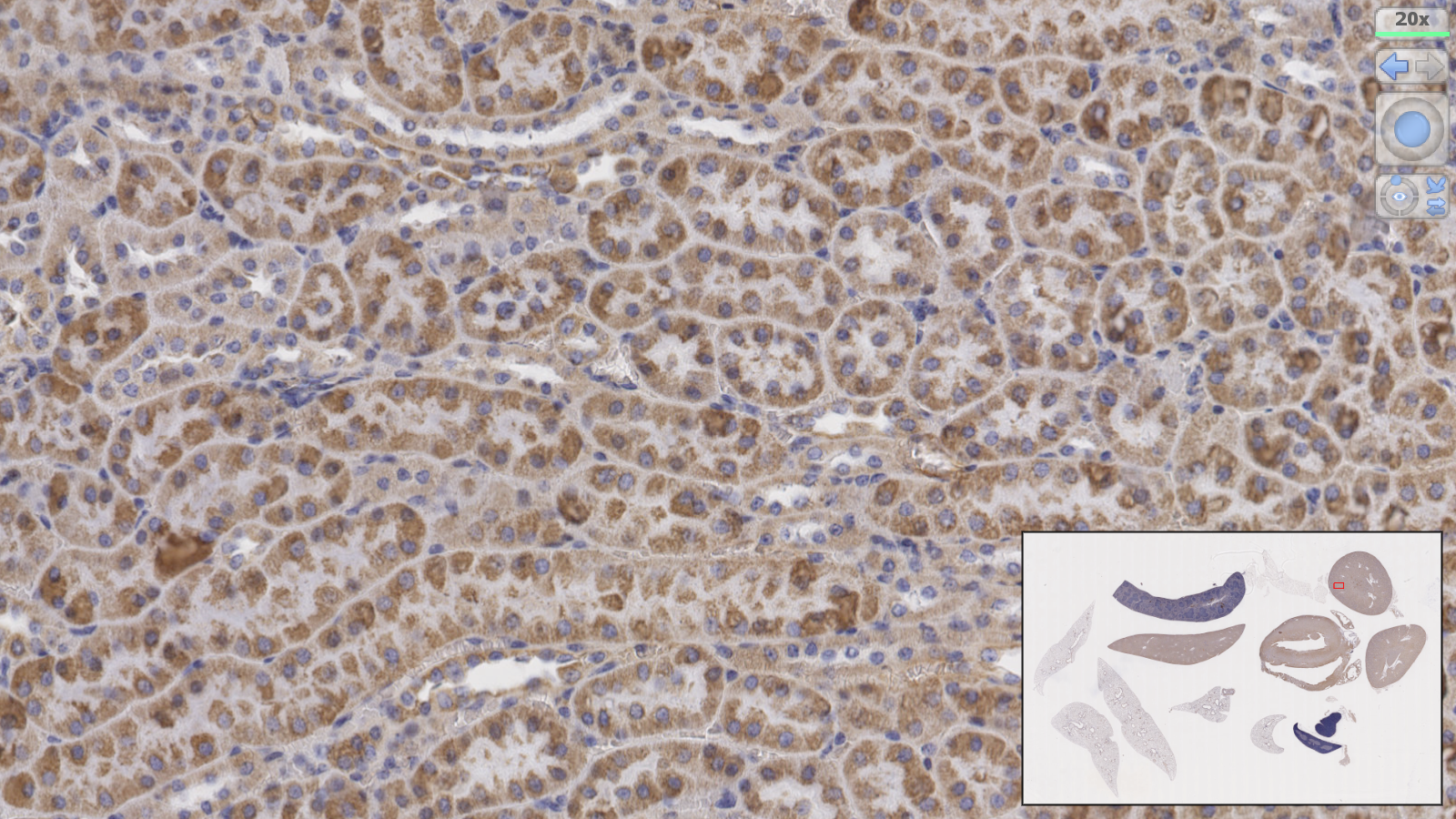

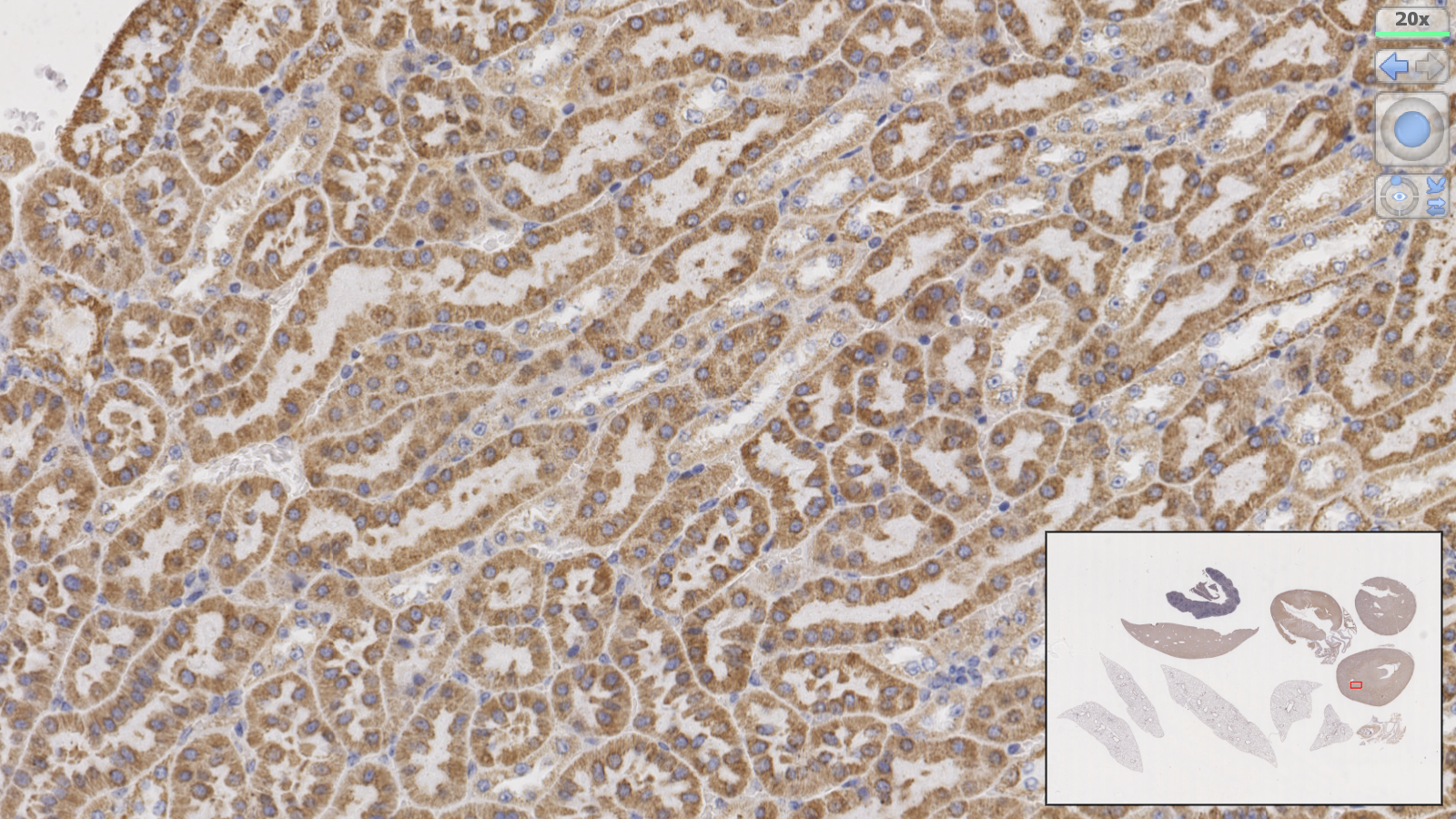


***Uqcrh*^-/-^**

**WT**

**Kidney**

**VDAC1/Porin**

**VDAC1/Porin**

**VDAC1/Porin**

**VDAC1/Porin**

**VDAC1/Porin**

**VDAC1/Porin**

**Heart**

**Liver**


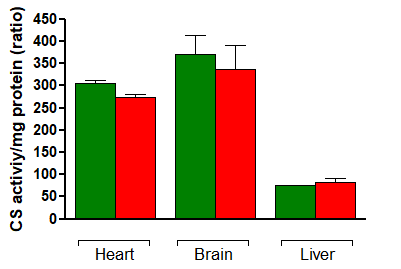


WT

*Uqcrh*^-/-^

**A** Citrate synthase (CS) activity in heart, brain and liver, of wild-type and *Uqcrh*^-/-^ mice. Data are normalised to the mg of protein. Values are given as mean ± SD, n = 4 WT, 5 *Uqcrh*^-/-^ (Heart), 3 WT, 3 *Uqcrh*^-/-^ (Brain), 1 WT and 2 *Uqcrh*^-/-^ (Liver). 2-way ANOVA (Multiple comparison).
**B** Immunohistochemical staining of VDAC1/porin was performed on liver, heart and kidney derived from wild-type and *Uqcrh*^-/-^ mice at 9 weeks of age.

## Appendix Fig S2 - Human megacomplex structure


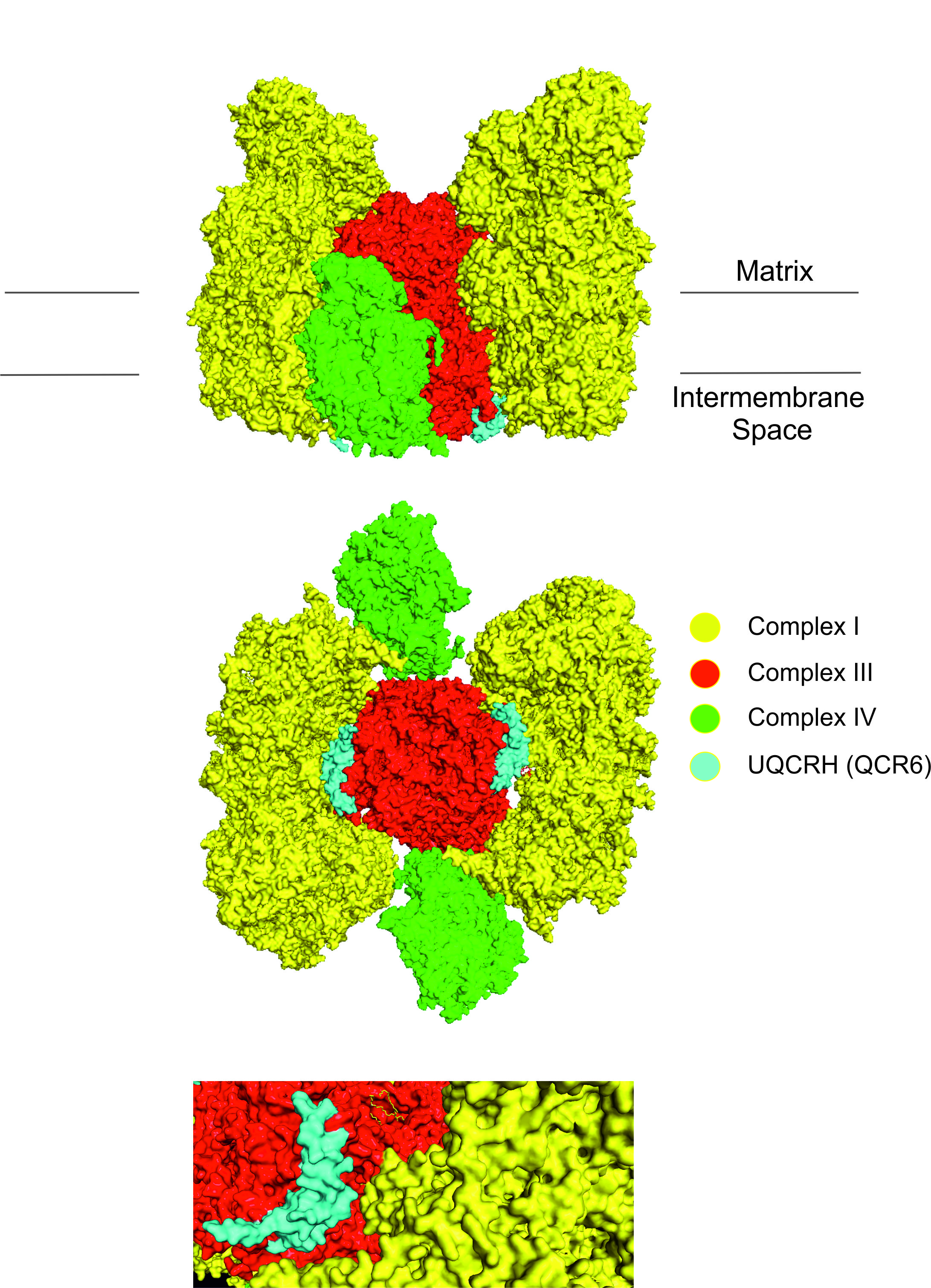


**A**

**B**

**C**

**A** Side view of human Megacomplex (CI_2_:CIII_2_:CIV_2_) structure.

**B** View from intermembrane site of human Megacomplex (I_2_III_2_IV_2_) structure.

**C** Magnification of UQCRH (QCR6) interaction with complex I within human Megacomplex (CI_2_:CIII_2_:CIV_2_) structure.

Complex I, yellow; Complex III, red; Complex IV, green; UQCRH (QCR6) in cyan. UQCRH (QCR6) has no direct contact to complex I and IV suggesting that an absence of UQCRH (QCR6) does not interfere with association to complex I. Interesting in this structure complex III has no contact with complex IV (B). Structure from (Guo et al., 2017) (PDB 5XTI).

## Appendix Table S1 - Comprehensive clinical history of patients

Table with a comprehensive clinical history of both patients and systematic analysis of phenotypes observed in patients

|  | **Individual II-1** | **Individual II-4** |
| --- | --- | --- |
| **Birth history** | Birth at 38 weeks of gestation, normal pregnancy and delivery | Birth at 39 weeks of gestation, normal pregnancy and delivery |
| **Initial presentation** | 2 years | 3 years 4 months |
| **Acute episode presentation** | Vomiting, diarrhoea, confusion | Vomiting, diarrhoea, confusion |
| **Biochemistry at acute presentation** | raised plasma lactate  raised ammonia  hypoglycemia | raised plasma lactate  raised ammonia  hypoglycemia |
| **Trigger for acute episode** | mild unspecified viral illness | mild unspecified viral illness |
| **Clinical examination findings** | Left microtia, profound sensorineural and conductive hearing loss (complete occlusion of the ear canal and hypoplastic inner ear structures) | Nil of note |
| **Growth** | age 11.5 years: weight 38.6 kg (<75th centile) height 133 cm (<9th centile) | age 8 years: weight 25.2 kg (<50th centile) height 120 cm (<9th centile) |
| **Development** | Mild speech delay noted at age 3yr (likely secondary to hearing impairment) otherwise normal | Normal |
| **Investigations (additional)** | CT head, MR brain, echocardiogram all normal | MR brain normal |
| **Last follow up** | 12 years | 8 years |

## Appendix Table S2 - Clinical chemistry parameters measured in plasma

Summary of clinical chemistry parameters measured in plasma of 8–12-week-old male and female *Uqcrh^-/-^* and wild-type controls under standard ad libitum fed conditions. Data are shown as median, quartiles and p-values of a Wilcoxon test.

|  | **Male** | | **Female** | | **Male** | **Female** | **Overall** |
| --- | --- | --- | --- | --- | --- | --- | --- |
|  | WT | *Uqcrh^-/-^* | WT | *Uqcrh^-/-^* |  |  |  |
|  | n=8 | n=7 | n=7 | n=9 |  |  |  |
|  | Median [25%, 75%] | Median [25%, 75%] | Median [25%, 75%] | Median [25%, 75%] | p-value | p-value | p-value |
| **Potassium [mmol/**L**]** | 4.1 [4, 4.1] | 5.3 [5.1, 5.8] | 5 [4.8, 5.3] | 6.5 [5.4, 6.9] | < 0.001 | 0.003 | < 0.001 |
| **Total protein [g/**L**]** | 48.2 [47.1, 50.6] | 37.9 [36.5, 43.5] | 47.5 [46.5, 48.8] | 38.7 [37.2, 41] | < 0.001 | < 0.001 | < 0.001 |
| **Albumin [g/**L**]** | 26.4 [25.9, 27.8] | 19.2 [17.8, 22.1] | 24.4 [23.6, 24.6] | 21.5 [18, 23.5] | < 0.001 | 0.006 | < 0.001 |
| **ALAT/GPT [U/**L**]** | 24 [20, 31] | 109 [100, 120] | 22 [20, 26] | 146 [93, 159] | < 0.001 | < 0.001 | < 0.001 |
| **ASAT/GOT [U**/L**]** | 66 [62, 80] | 143 [124, 258] | 66 [54, 82] | 279 [173, 333] | < 0.001 | < 0.001 | < 0.001 |
| **Glucose [mmol/**L**]** | 15.54 [14.98, 16.27] | 64.17 [61.95, 66.13] | 17.1 [14.57, 20.93] | 63.8 [60.63, 68.06] | < 0.001 | < 0.001 | < 0.001 |
| **ALP [U/**L**]** | 164 [152, 167] | 268 [232, 287] | 94 [92, 101] | 300 [256, 342] | < 0.001 | < 0.001 | < 0.001 |
| **Total iron binding capacity [µmol/**L**]** | 55.9 [54.3, 60.8] | 41.7^a^ [39.1, 42.7] | 59.1 [56.9, 61.4] | 36.8^a^ [35.7, 37.4] | 0.001 | 0.003 | < 0.001 |

^a^ Number not based on the full number of animals: 6 female and 5 male *Uqcrh^-/-^*.

## Appendix Table S3 – Patient versus mouse comparison

A summary of patient examination in comparison to the results of the *Uqcrh^-/-^* phenotyping to date week 6, allowing a patient/mouse aged-matched overview of the human and mouse model data. (NA = data not available).

|  | **Patient II:1** | **Patient II:4** | ***Uqcrh^-/-^* mouse** |
| --- | --- | --- | --- |
| **Birth history** | 38/40, normal pregnancy and delivery | 39/40, normal pregnancy and delivery | Normal pregnancy and delivery |
| **Family history** | Consanguineous, British Pakistani parents, two younger siblings, who are well | Consanguineous, British Pakistani parents (related to parents of II-1), only child | Consanguineous  13,66 % homozygous pups, viable, but slightly reduced number of *Uqcrh^-/-^* offspring |
| **Clinical examination findings (when well)** | Left microtia, profound sensorineural and conductive hearing loss (complete occlusion of the ear canal and hypoplastic inner ear structures) | NA | NA |
| **Growth** | Height and weight were on the 2^nd^ centiles at age 3 years | NA | Mutants are smaller in size:  Lower body weight in comparison to wild-type from 4 weeks onwards |
| **Development** | Mild speech delay noted age 3 years  No problems noted after hearing aid fitted | NA | NA |
| **Investigations (additional)** | CT head, MR brain and echocardiogram were all normal |  | Cardiovascular phenotypic alterations in ECG and ECHO at 6 weeks of age;  Pathological examination: Tubular alterations in the kidney. Lesions begin at the age of 6 weeks and progress with age (data not shown, ongoing study) |
| **Summary of metabolic episodes** | Age 2 years, presented with vomiting illness and metabolic acidosis. Lactate was raised, no hypoglycemia. Recovered with fluid resuscitation  Re-presented collapsed after a vomiting illness with metabolic acidosis;  Presented average of 3-4 times per year with vomiting.  On 8 occasions by presentation had metabolic acidosis (lowest pH 7.2), with a raised lactate (up to 18.2 mmol/L), also hypoglycemic at times (lowest glucose 1.0 mmol/L). Ammonia was raised (up to 301 μmol/L), but decreased rapidly. Liver function was normal.  Aged 8-12 years presented twice with vomiting, investigations were normal.  Well in between episodes. | Age of 3 years 4 months, presented with vomiting and diarrhea, high lactate and compensated metabolic acidosis.  Presented average of 8 times per year to hospital over next 3 years, vomiting, fever or abdominal pain. There was a high lactate on 4 occasions (maximum 10mmol/L).  One occasion, age of 4 years and 8 months, he presented with a pH of 7.046, lactate 10 mmol/L and ammonia of 306 μmol/L. The blood glucose was 0.5 mmol/L.  One episode of pancreatitis age 8 years, associated with hypoglycemia, hyperammonemia, and acidosis.  Well in between episodes. | 1–2-week-old mice were hypo- or normoglycemic; this changed with age to hyperglycemia.  Increased lactate levels |
