# Supplementary material for: Characterising a homozygous two‐exon deletion in UQCRH: comparing human and mouse phenotypes
Source: EMBO Mol Med. 2021 Nov 8;13(12):e14397. doi: 10.15252/emmm.202114397 (PMC8649870; doi:10.15252/emmm.202114397)
Supplement: Supplementary file 2 — Expanded View Figures PDF [file EMMM-13-e14397-s002.pdf]

Expanded View Figures

Figure EV1. Exome sequence analysis.

A Table detailing the homozygous regions identified in the two affected first cousin individuals.

B Read depth image from exome sequence data from the affected individual (II-1) demonstrating absences of sequence reads encompassing exons 2 and 3 of UQCRH (upper panel) and evidence of sequence reads for both exons in an unrelated control (lower panel).

C Log_2 ratio SNP microarray results show the absence of signal for a single probe between exons two and three of UQCRH (indicated by a red circle) in an affected individual (II-1) upper track and evidence of two copies of this probe in their unaffected sibling (II-2) in the lower track.

 © 2021 The Authors

A

Chromosome	Start	End	Size (Mb)
Chr1	37,513,756	47,573,413	10.1
Chr1	49,375,490	52,032,789	2.7

B

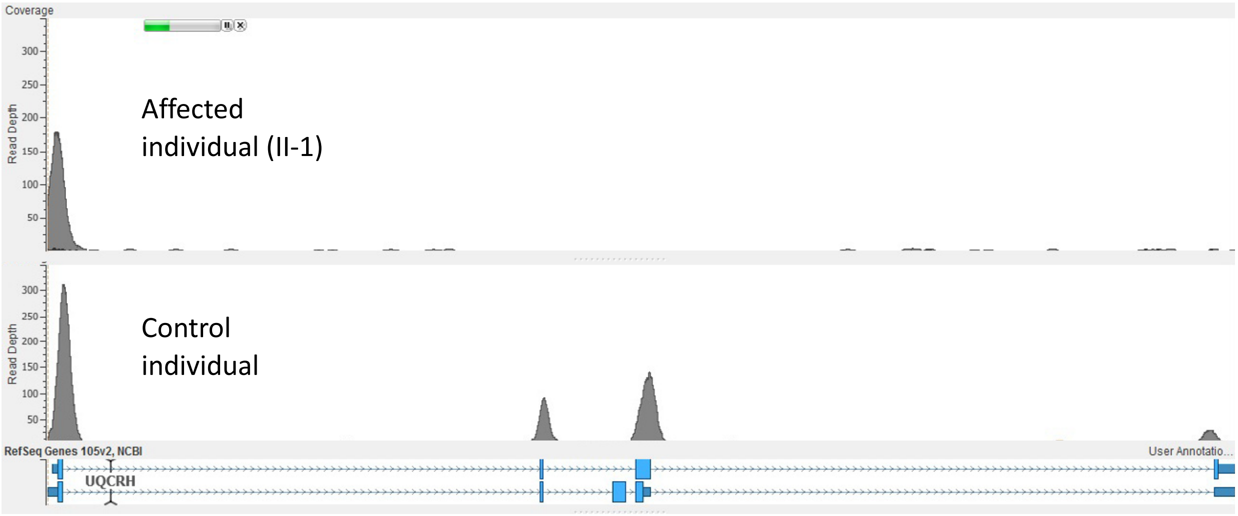

C

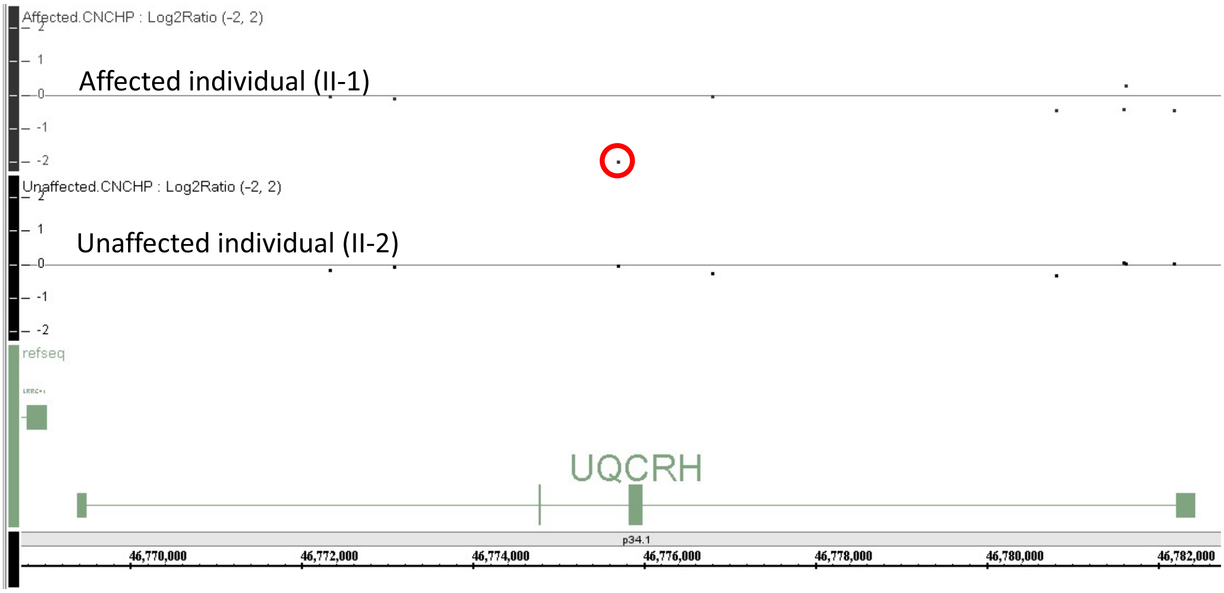

Figure EV1.

Figure EV2. Histology of the heart (haematoxylin and eosin staining and electron microscopy).

A Representative photomicrographs of heart tissue, stained with haematoxylin and eosin, from wild-type (WT, upper panel) and *Uqcrh*$^{-/-}$ (lower panel) mice at 9 weeks of age. Normal histopathological structure of the myocardium (red box, central panels) and aorta (black box, right panels) were observed in both groups of mice. Scale bars represent 1 mm in the left panels and 50 μm in the central and right panels.

B Electron microscopy images of 3 representative wild-type (WT, upper panel) and 3 *Uqcrh*$^{-/-}$ mice (lower panel) heart mitochondria. Mitochondria from Uqcrh$^{-/-}$ mouse heart tissue showed paracrystalline inclusions (arrows). Magnification 1:50,000.

 © 2021 The Authors

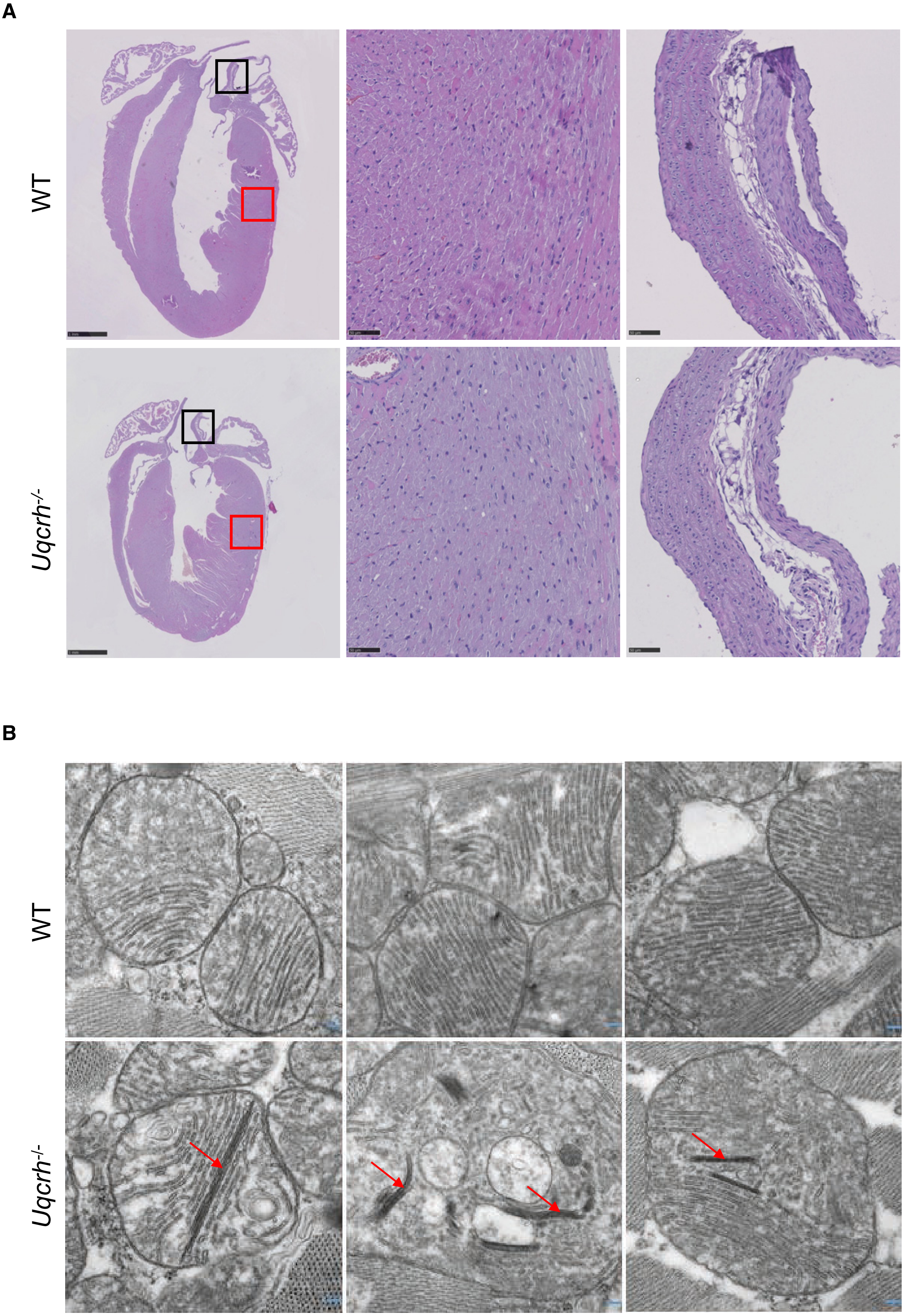

Figure EV2.

Figure EV3. BN-PAGE of multiple mouse tissues show increased staining for large supercomplex (S_{XL}) in *Uqcrh*$^{-/-}$ mice.

A Mitochondrial membranes from kidney samples of wild-type (WT) and *Uqcrh*$^{-/-}$ mice were solubilised with digitonin and separated on native gradient gels. Protein complexes were stained with Coomassie (left panel) with NADH:NTB reductase activity stain (centre panel) and with an antibody against Core II subunit of complex III (Uqcrc2, right panel).

B Mitochondrial membranes from brain samples of wild-type (WT) and *Uqcrh*$^{-/-}$ mice were solubilised with digitonin and separated on native gradient gels. Protein complexes were stained with Coomassie (left panel) with NADH:NTB reductase activity stain (right panel).

C Mitochondrial membranes from skeletal muscle samples of wild-type (WT) and *Uqcrh*$^{-/-}$ mice were solubilised with digitonin and separated on native gradient gels. Protein complexes were stained with Coomassie (left panel) with NADH:NTB reductase activity stain (right panel).

D Mitochondrial membranes from liver samples of wild-type (WT) and *Uqcrh*$^{-/-}$ mice were solubilised with digitonin and separated on native gradient gels. Protein complexes were stained with Coomassie (left panel) with NADH:NTB reductase activity stain (right panel).

Data information: Assignment of complexes: I, complex I; III_2, complex III dimer; IV, complex IV; S_0, supercomplex containing complex I and a dimer of complex III; S_1, supercomplex containing complex I, a dimer of complex III and 1 copy of complex IV, S_{XL}, Supercomplex or Megacomplex containing I, III and higher signals also additional copies of IV.

© 2021 The Authors

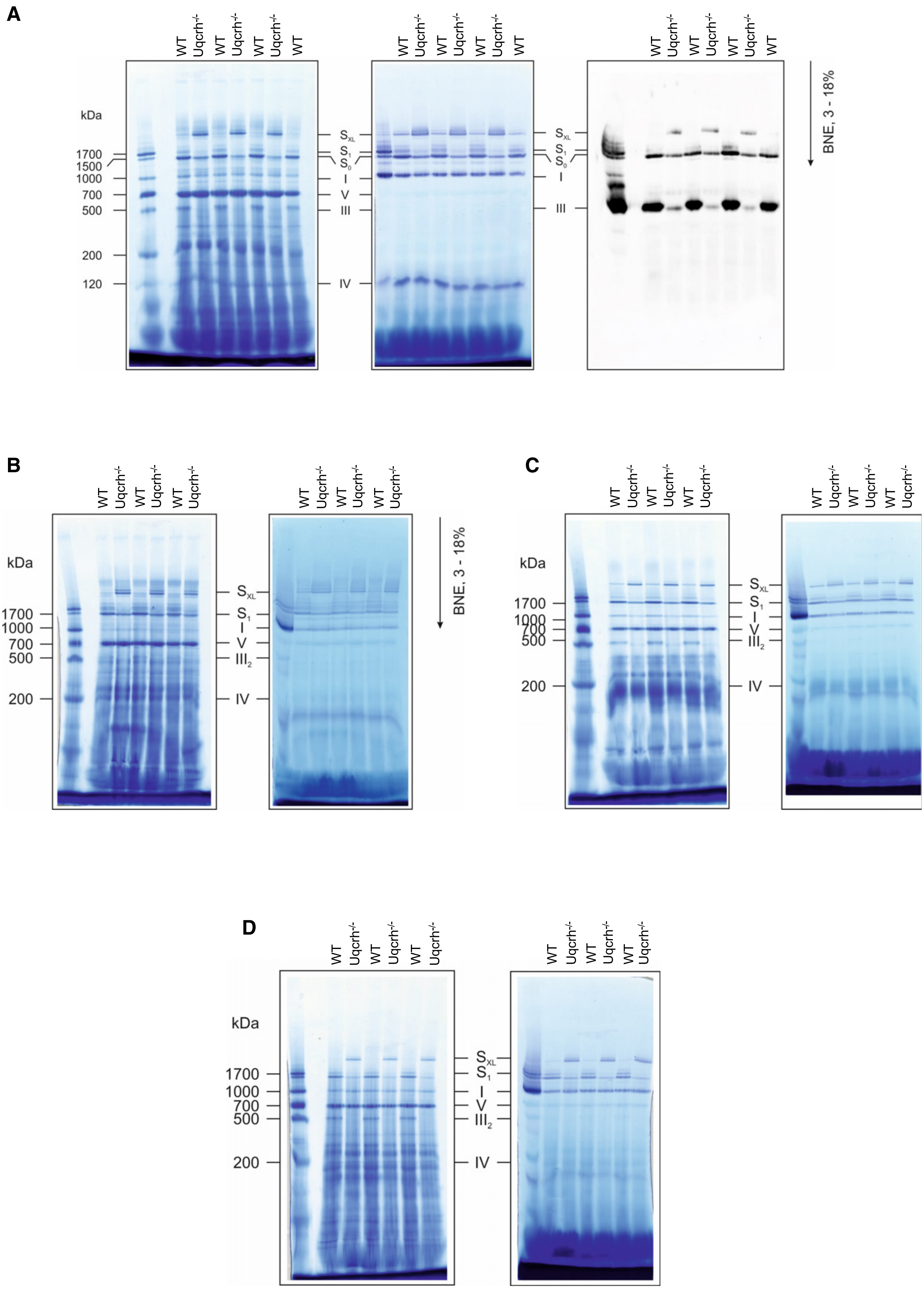
A
WT
Uqcrh-/-
kDa
1700
1500
1000
700
500
200
120
SXL
S1
S0
I
V
III
IV
BNE, 3 - 18%
B
C
III2
D

Figure EV3.

Figure EV4. Complexome analysis of patient fibroblasts and *Uqcrh*$^{-/-}$ mouse heart tissue.

A Mitochondrial membranes of control and patient fibroblasts were solubilised with digitonin and separated on native gradient gels. Each native lane was cut into even fractions from high to low molecular mass. Proteins in each slice were digested with trypsin and analysed by quantitative mass spectrometry.

B Mitochondrial membranes of heart from wild-type and *Uqcrh*$^{-/-}$ deficient mice were solubilised with digitonin and separated on native gradient gels. Each native lane was cut into even fractions from high to low molecular mass. Proteins in each slice were digested with trypsin and analysed by quantitative mass spectrometry.

Data information: All data were analysed together and complexome profiles were generated (see PRIDE repository PXD022856 and PXD022855). Assignment of complexes: I, complex I; III_2, complex III dimer; IV, complex IV; S_0, supercomplex containing complex I and a dimer of complex III; S_1, supercomplex containing complex I, a dimer of complex III and 1 copy of complex IV, S_{XL}, supercomplex or megacomplex containing I, III and higher signals also additional copies of IV.

© 2021 The Authors

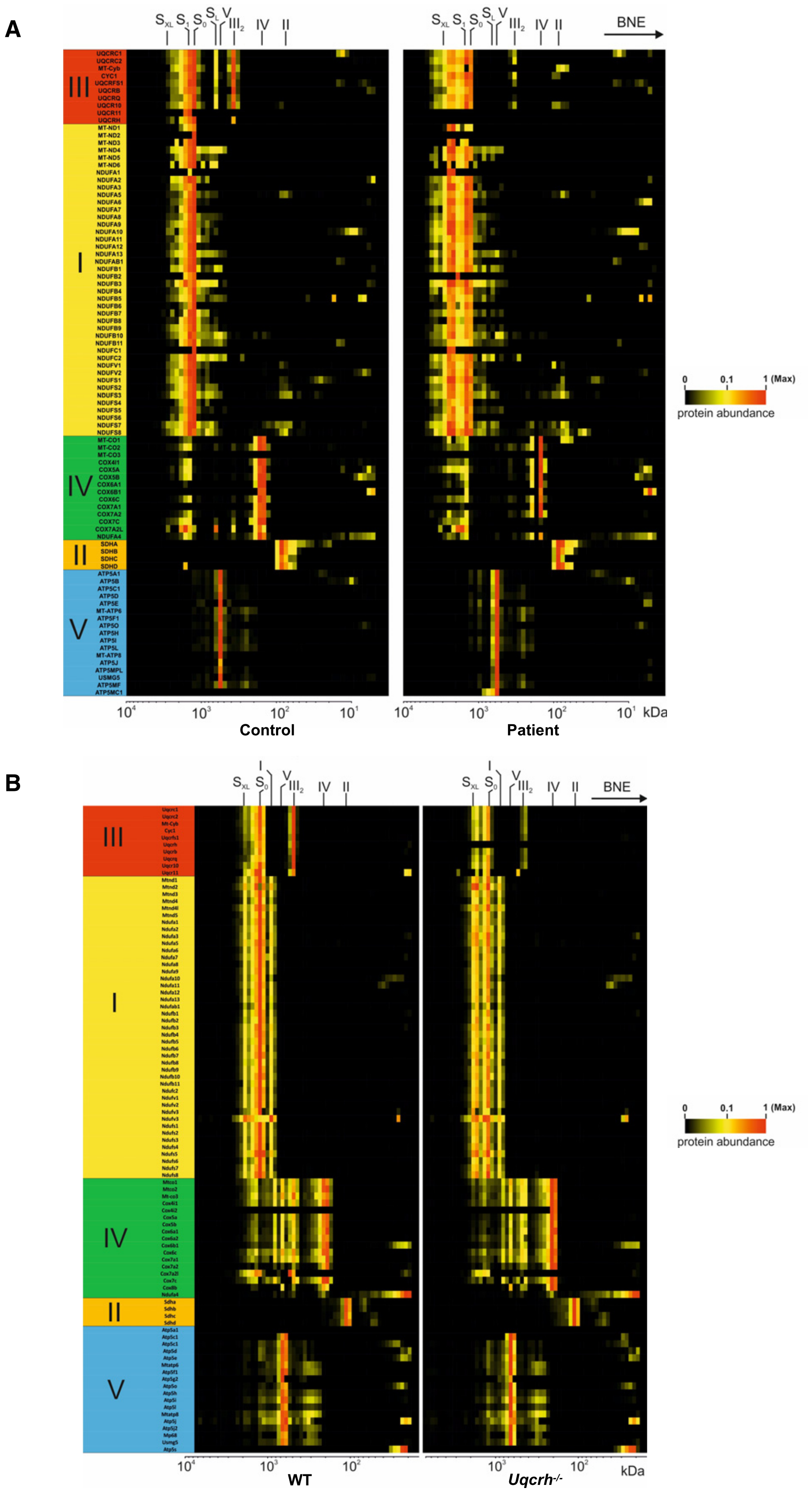
A
BNE
III
I
IV
II
V
kDa
Control
Patient
0
0.1
1 (Max)
protein abundance
B
WT
Uqcrh-/-

Figure EV4.

Figure EV5. Alteration of the relative distribution of individual respiratory chain complexes and supercomplexes.

Mitochondrial membranes from fibroblasts of control (WT) and patient fibroblasts (upper panels, A–G) and heart tissue from wild-type (WT) and Uqcrh$^{-/-}$ mice (lower panels, B–J) were analysed by complexome profiling and the appearance of individual complexes and superassemblies were quantified.

A, B The average of IBAQ values of all identified subunits from complexes III, I and IV (full data including all individual subunits shown in Fig EV4).
C, D Molecular mass region used to quantify the appearance of individual and supercomplexes.
E, H Sum of all IBAQ values and relative distribution of complex III as individual dimer or assembled into supercomplexes and large supercomplexes.
F, I Sum of all IBAQ values and relative distribution of complex I as individual or assembled into supercomplexes and large supercomplexes.
G, J Sum of all IBAQ values and relative distribution of complex IV as individual or assembled into supercomplexes.

Data information: Assignment of complexes: I, complex I; III2, complex III dimer; IV, complex IV; S0, supercomplex containing complex I and a dimer of complex III; S1, supercomplex containing complex I, a dimer of complex III and 1 copy of complex IV, SXL, Supercomplex or Megacomplex containing I, III and higher signals also additional copies of IV.

© 2021 The Authors

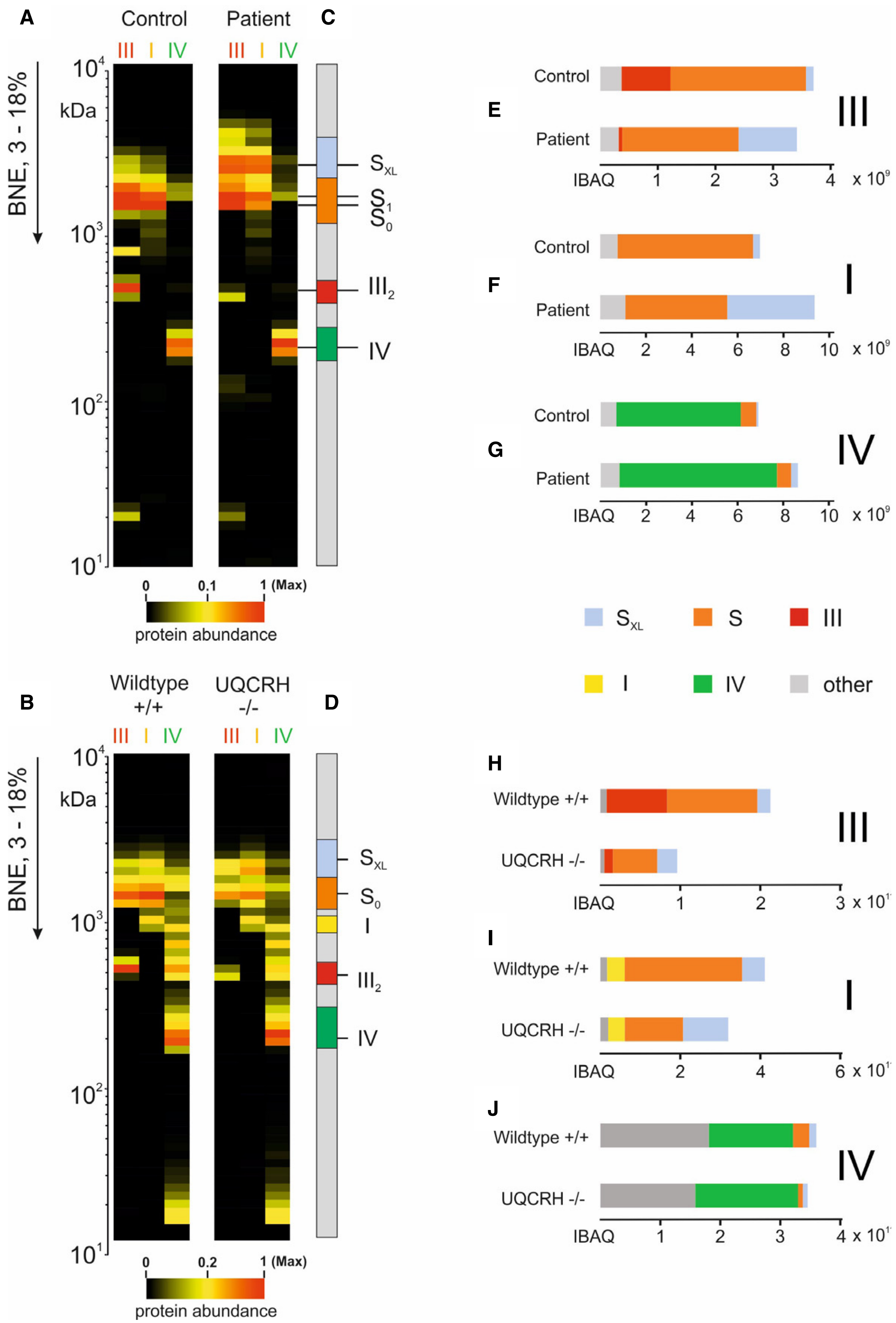
A
Control
Patient
C
III I IV
BNE, 3 - 18%
kDa
S_{XL}
S_1
S_0
III_2
IV
0 0.1 1 (Max)
protein abundance
B
Wildtype +/+
UQCRH -/-
D
S_0
I
0 0.2 1 (Max)
E
F
G
Control
Patient
IBAQ
x 10^9
S_{XL} S III I IV other
H
I
J
Wildtype +/+
UQCRH -/-

Figure EV5.
