# Supplementary material for: Characterising a homozygous two‐exon deletion in UQCRH: comparing human and mouse phenotypes
Source: EMBO Mol Med. 2021 Nov 8;13(12):e14397. doi: 10.15252/emmm.202114397 (PMC8649870; doi:10.15252/emmm.202114397)

Source Data Figure 3C

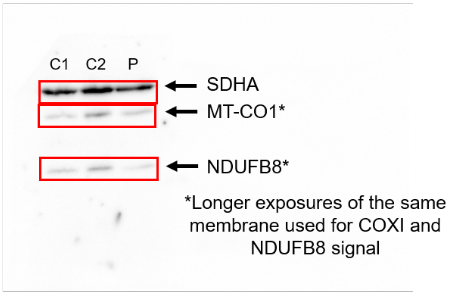

Colourimetric image to show MW marker (kDa)

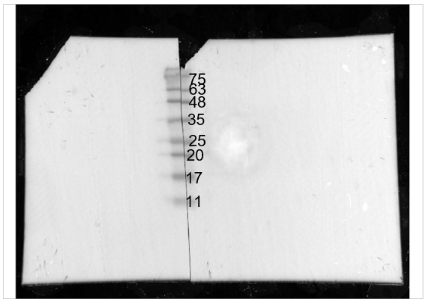

Merged image

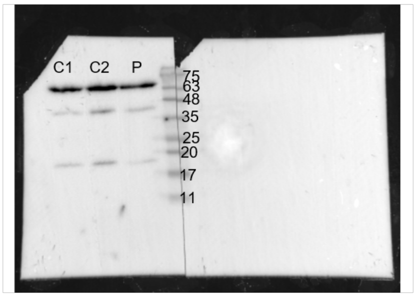

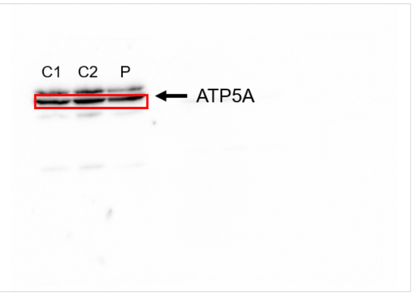

Colourimetric image to show MW marker (kDa)

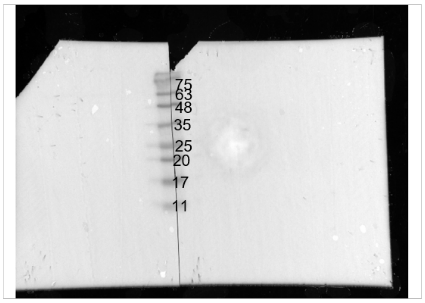

Merged image

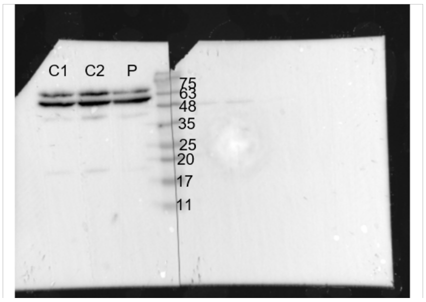

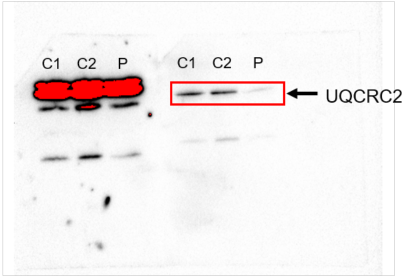

Colourimetric image to show MW marker (kDa)

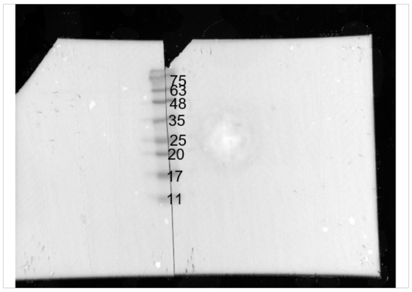

Merged image

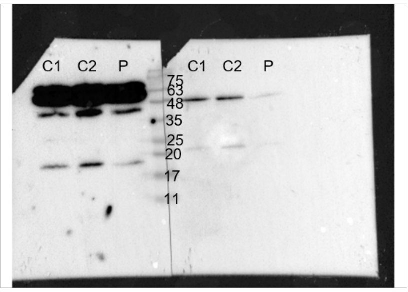

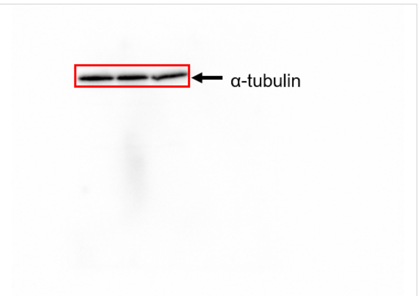

Colourimetric image to show MW marker (kDa)

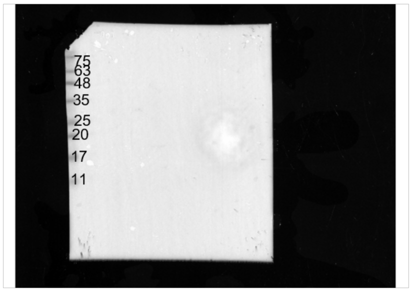

Merged image

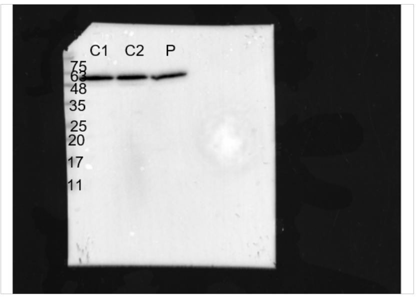

Source Data Figure 3D

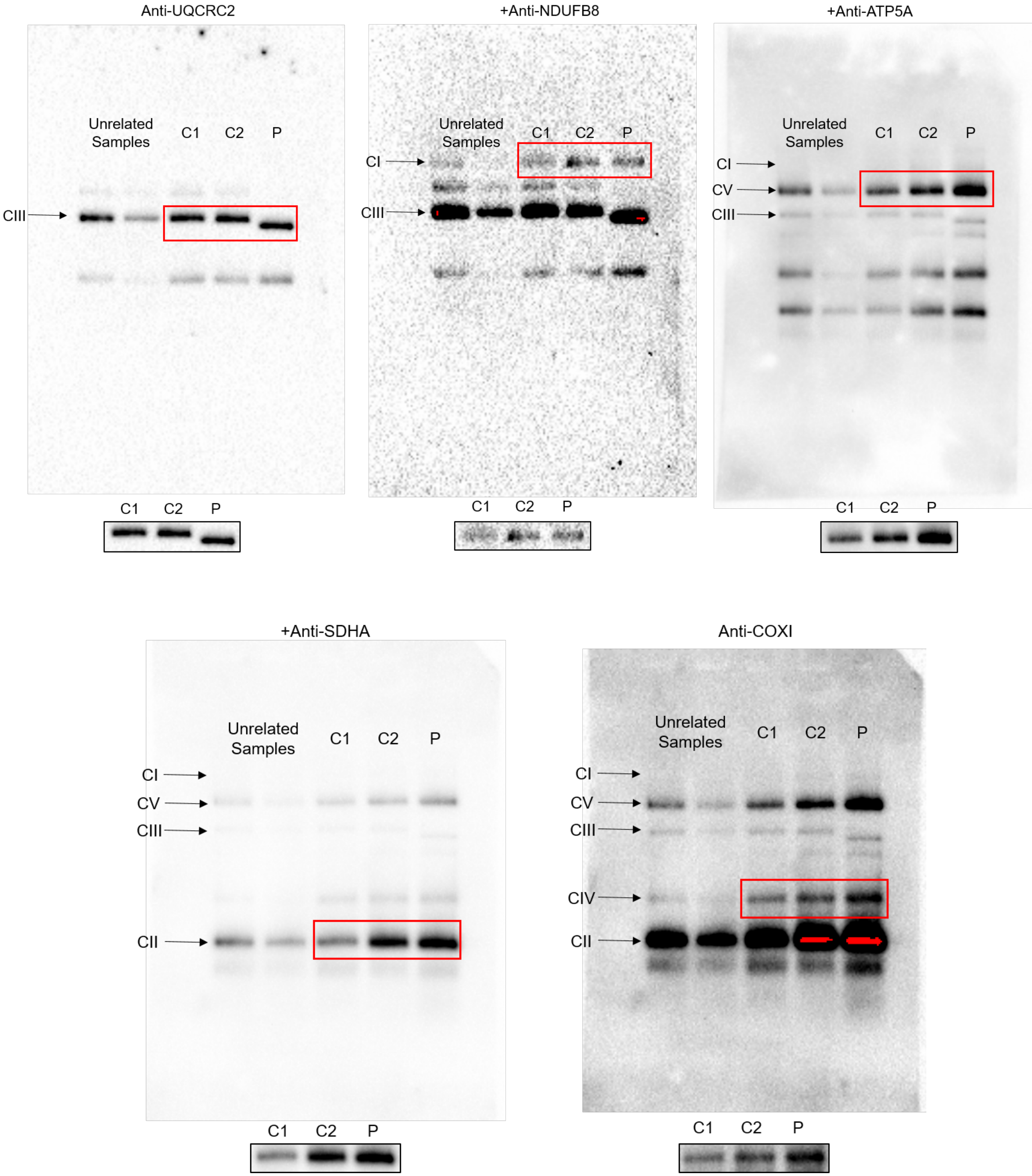

Source Data Figure 3E

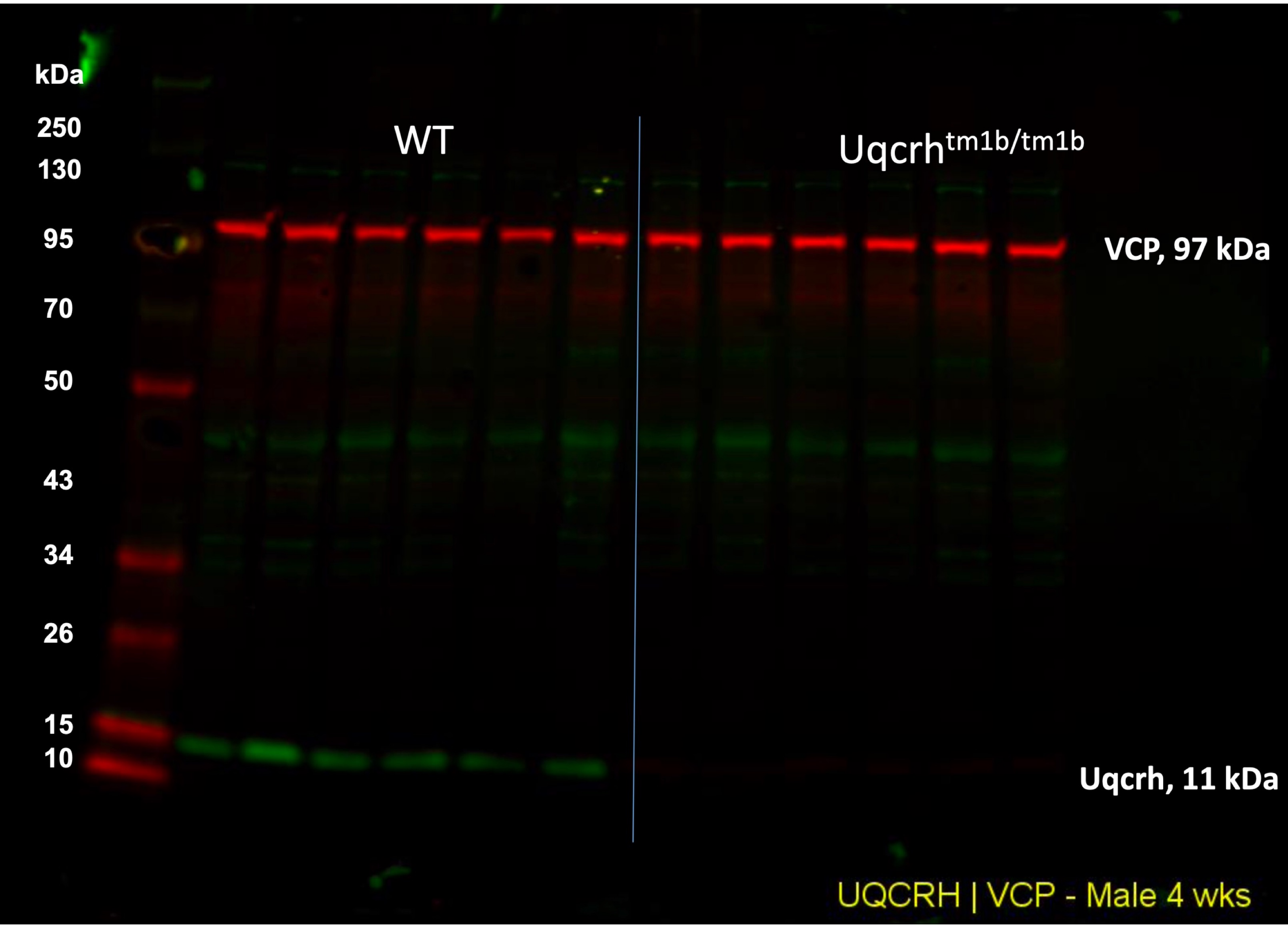

Source Data Figure 3F,G and Appendix Fig S1

Heart from Uqcrh$^{-/-}$
UQCRC2 staining

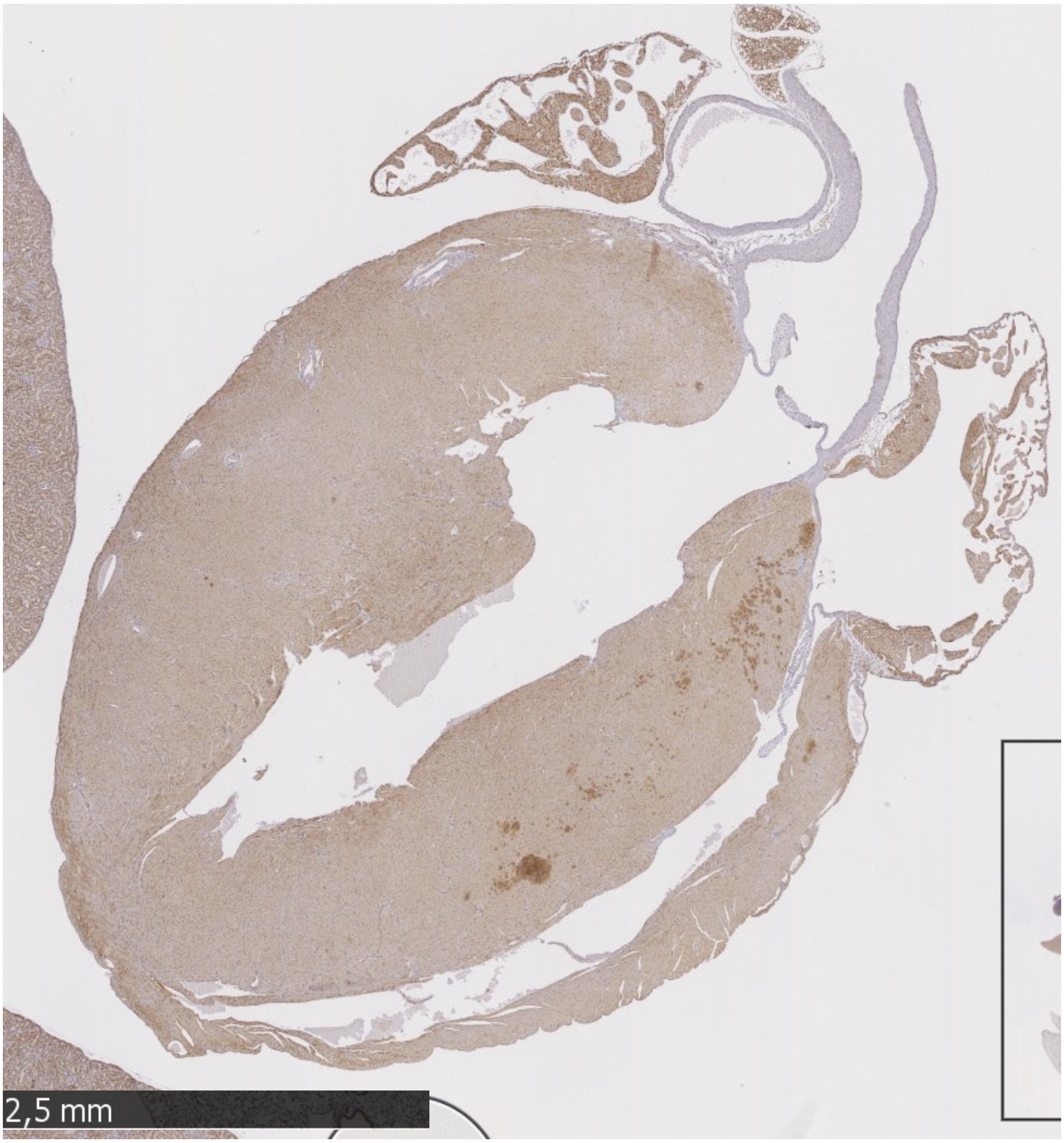

Heart from WT
UQCRC2 staining

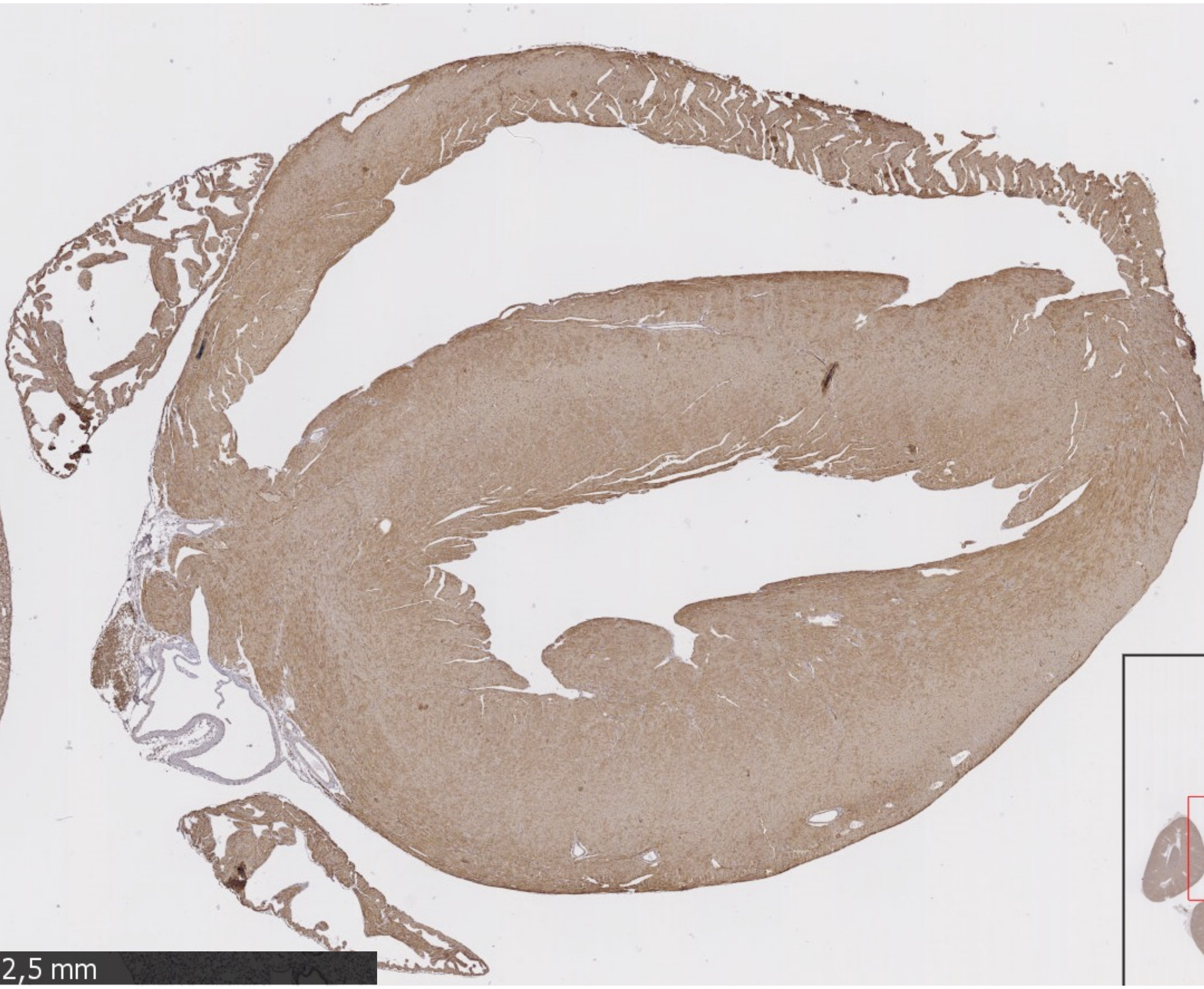

Liver from *Uqcrh*-/-
UQCRC2 staining

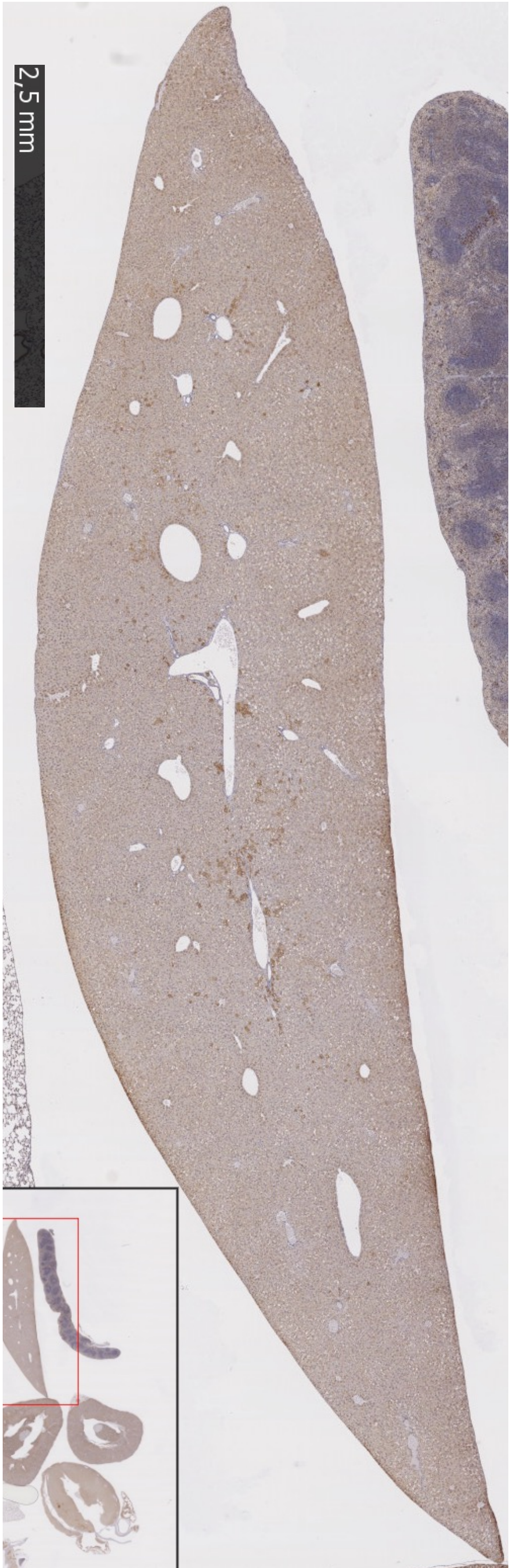

Liver from WT
UQCRC2 staining

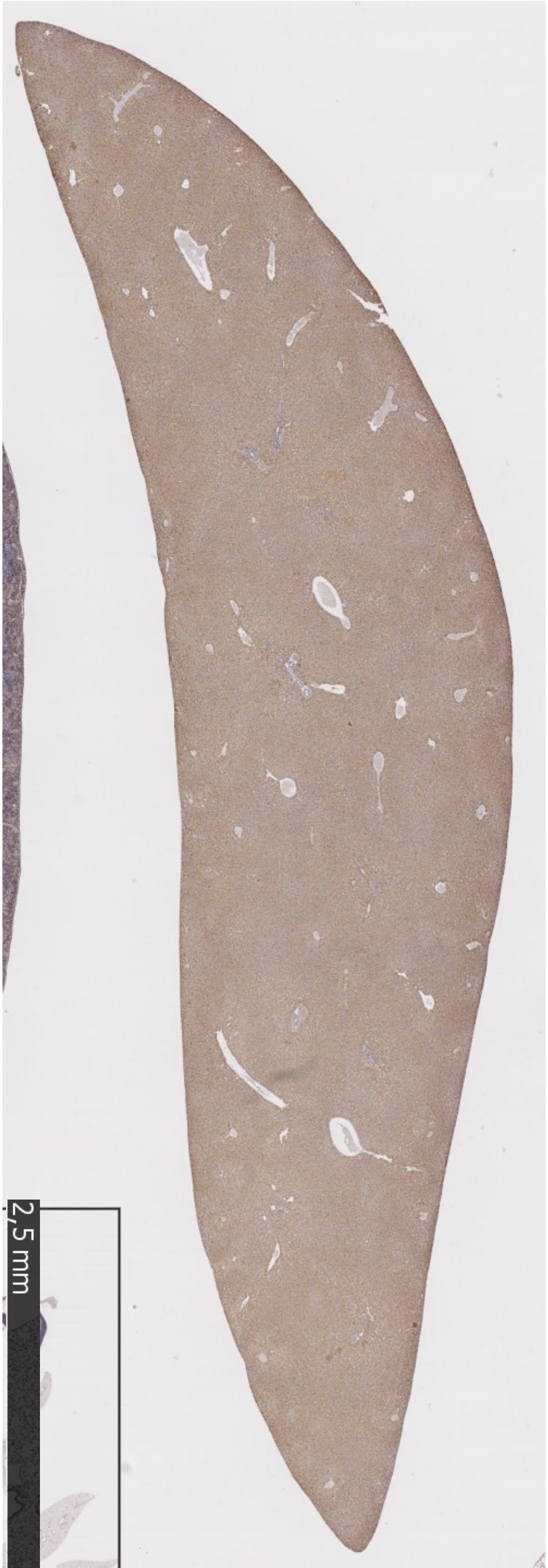

Kidney from *Uqcrh*$^{-/-}$
UQCRC2 staining

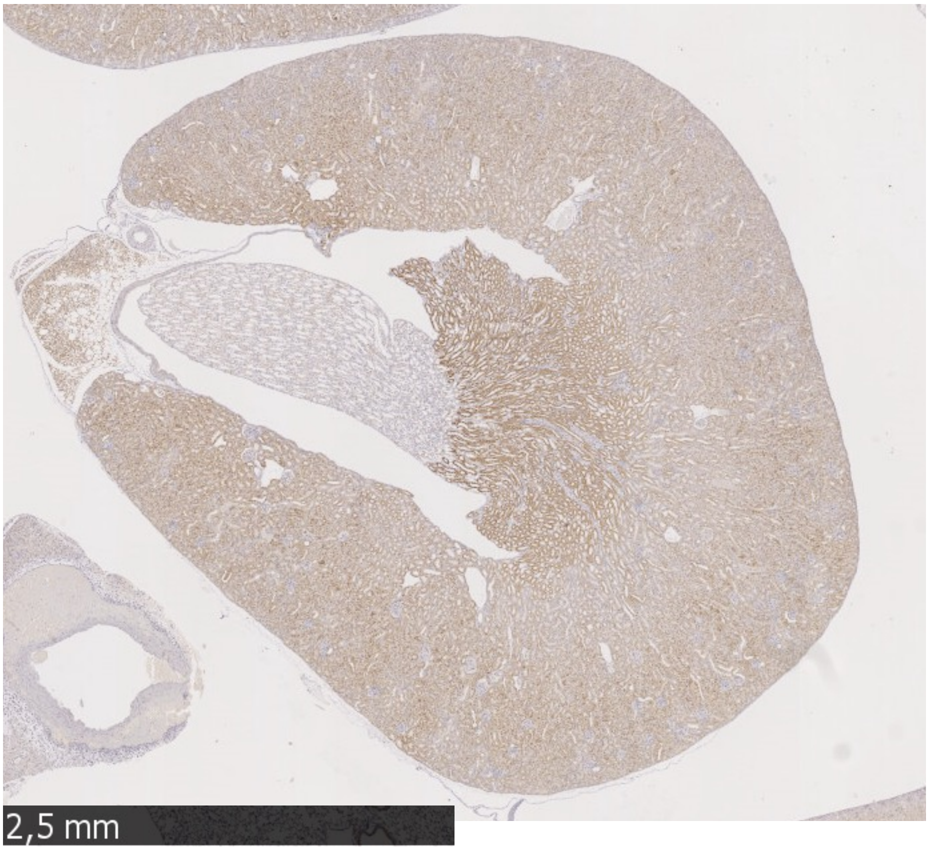

Kidney from WT
UQCRC2 staining

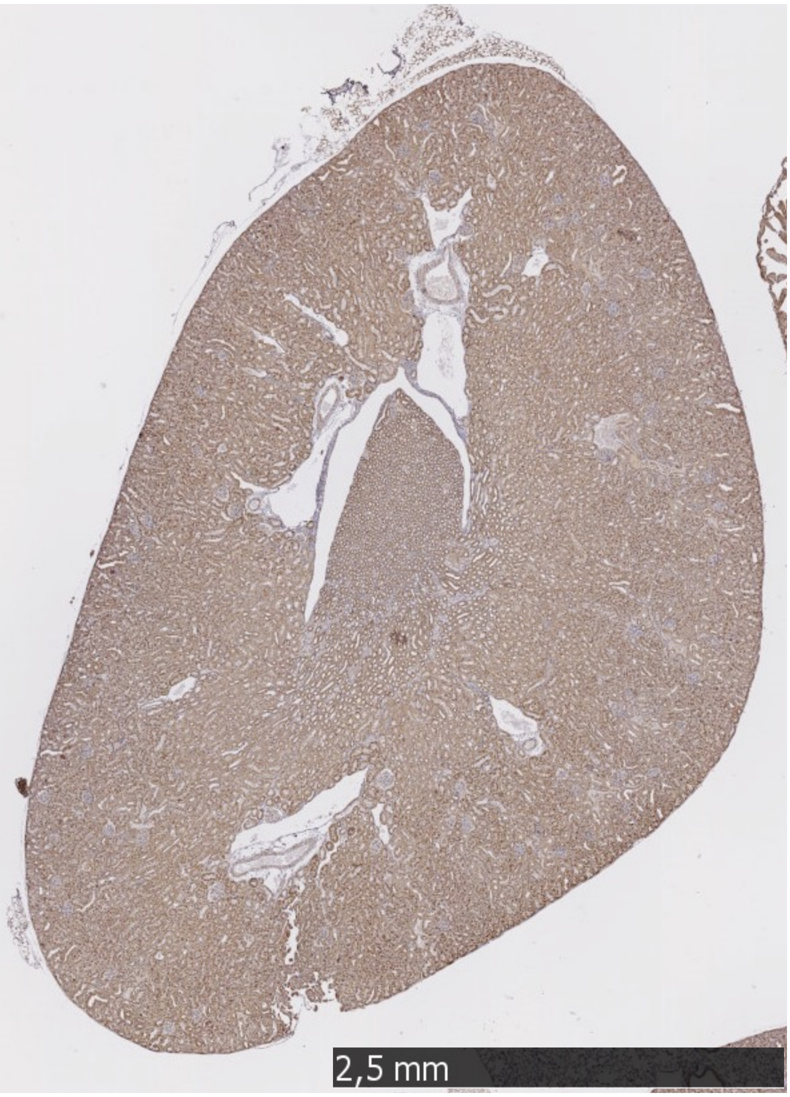

Heart from *Uqcrh*$^{-/-}$
VDAC1/Porin staining

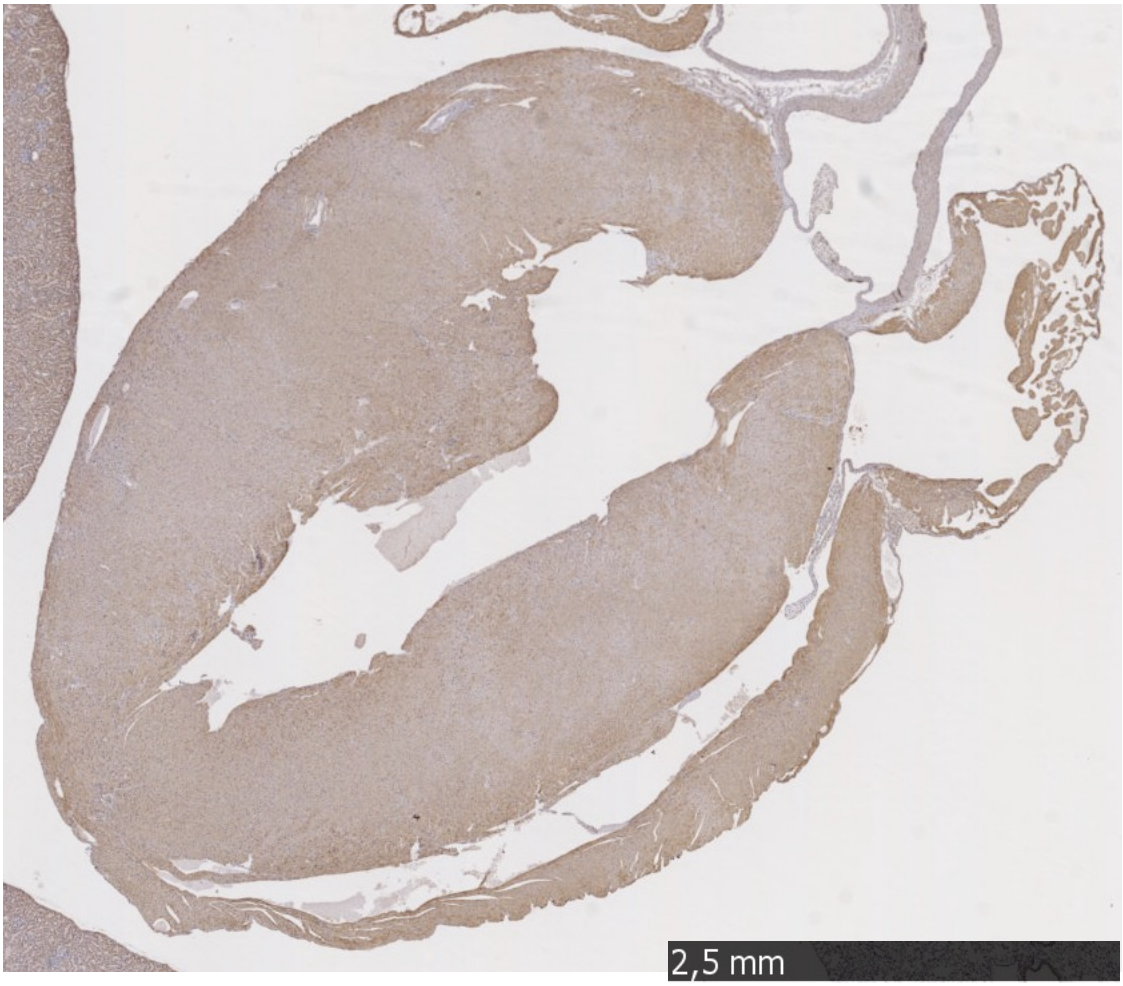

Heart from WT
VDAC1/Porin staining

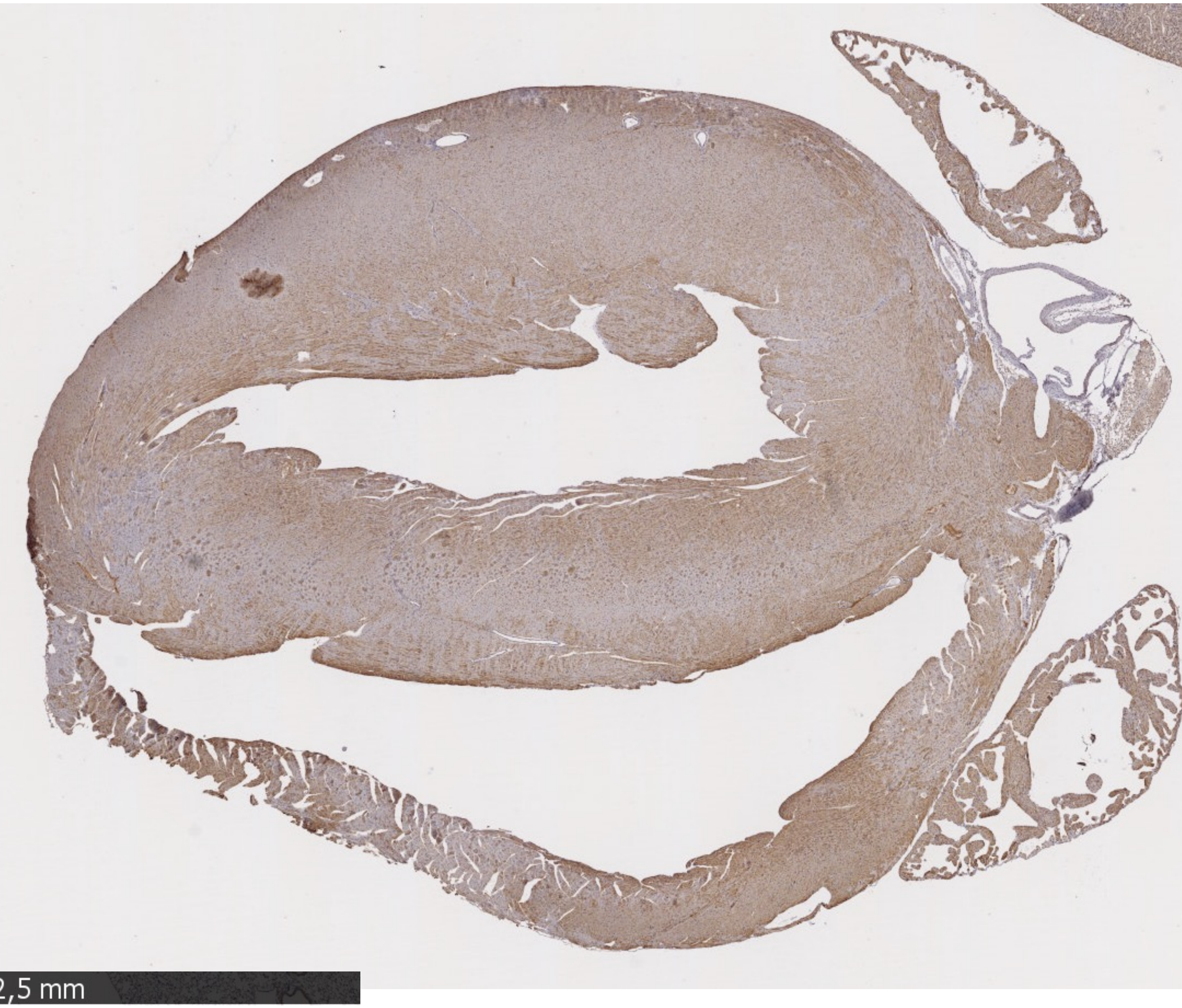

Liver from *Uqcrh*$^{-/-}$
VDAC1/Porin staining

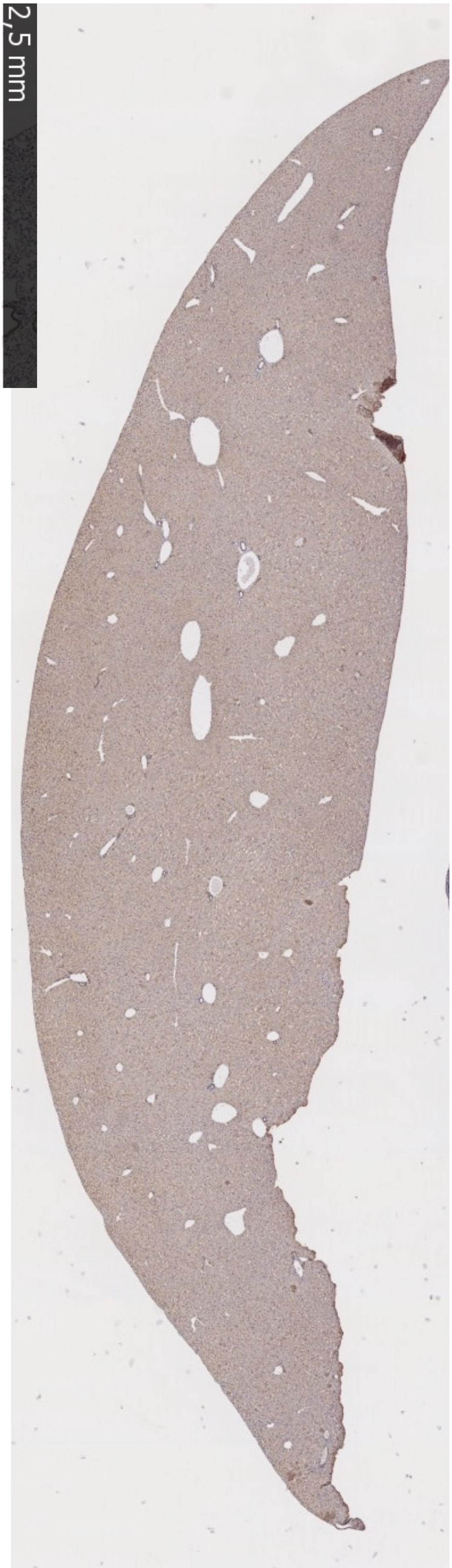

Liver from WT
UQCRC2 staining

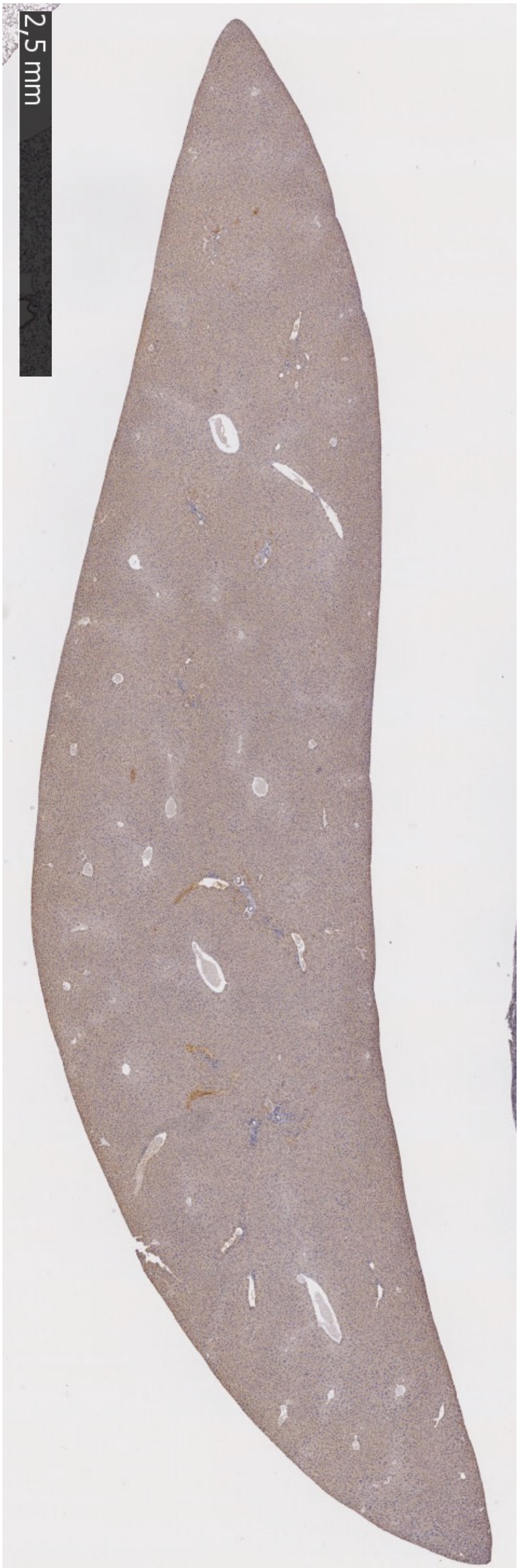

Kidney from *Uqcrh*$^{-/-}$
VDAC1/Porin staining

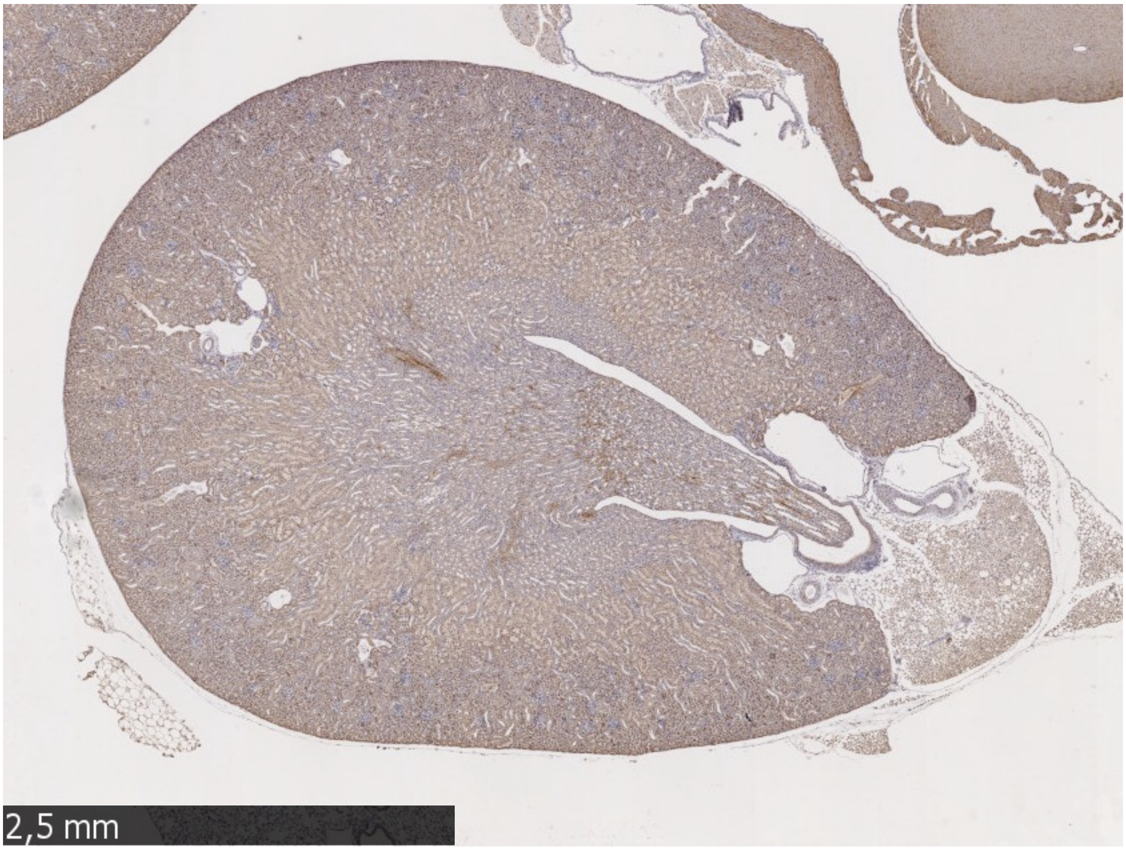

Kidney from WT
VDAC1/Porin staining

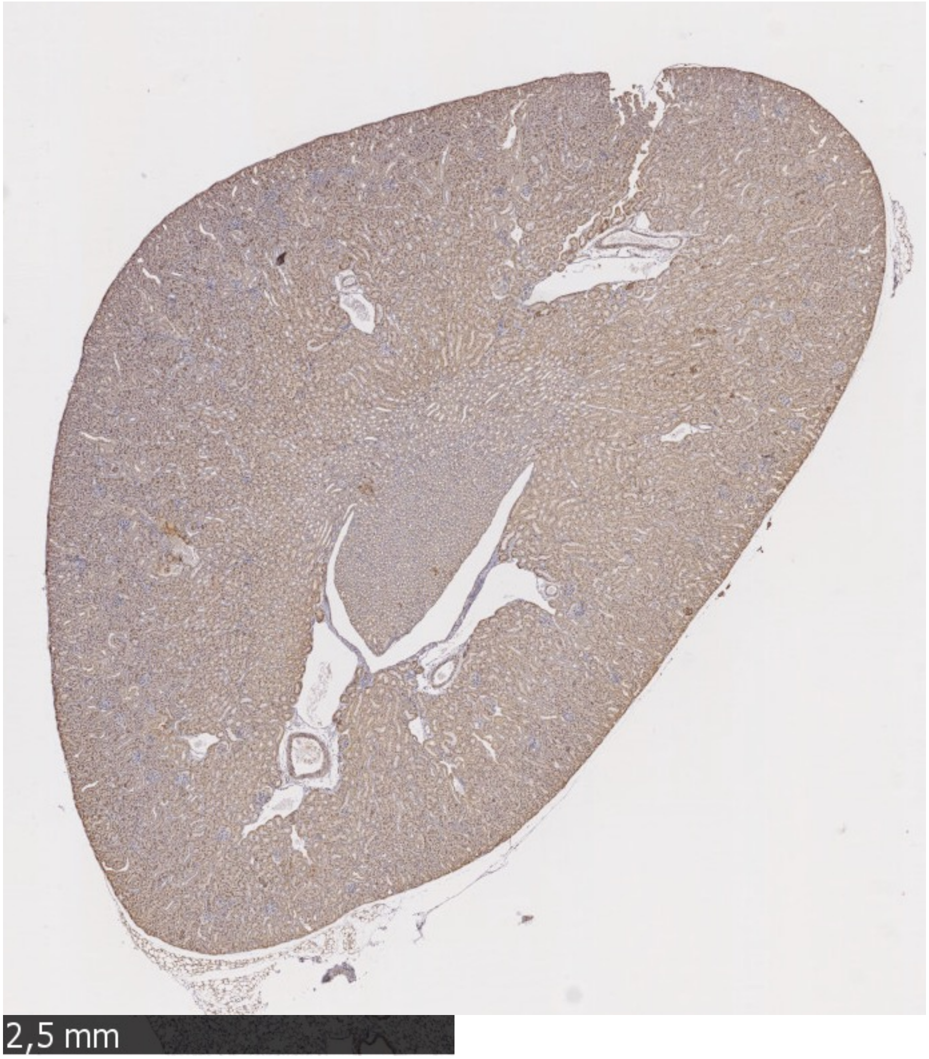

Supplement: Supplementary file 3 — Source Data for Figure 3 [file EMMM-13-e14397-s001.pdf]
