# Supplementary material for: Characterising a homozygous two‐exon deletion in UQCRH: comparing human and mouse phenotypes
Source: EMBO Mol Med. 2021 Nov 8;13(12):e14397. doi: 10.15252/emmm.202114397 (PMC8649870; doi:10.15252/emmm.202114397)
Supplement: Supplementary file 4 [file EMMM-13-e14397-s004.pdf]

EMBO Molecular Medicine

Characterising a homozygous two-exon deletion in UQCRH: comparing human and mouse phenotypes

Valerie Gailus-Durner, Silvia Vidali, Raffaele Gerlini, Kyle Thompson, Jill Urquhart, Jana Meisterknecht, Juan Aguilar-Pimentel, Oana Amarie, Lore Becker, Catherine Breen, Julia Calzada-Wack, Nirav Chhabra, Yi-Li Cho, Patricia da Silva-Buttkus, René Feichtinger, Kristine Gampe, Lilian Garrett, Kai Hoefig, Sabine Hölter, Elisabeth Jameson, Tanja Klein-Rodewald, Stefanie Leuchtenberger, Susan Marschall, Philipp Mayer-Kuckuk, Gregor Miller, Manuela Östereicher, Kristina Pfannes, Birgit Rathkolb, Jan Rozman, Charlotte Sanders, Nadine Spielmann, Claudia Stoeger, Marten Szibor, Irina Treise, John Walter, Wolfgang Wurst, Johannes Mayr, Helmut Fuchs, Ulrich Gärtner, Ilka Wittig, Robert Taylor, William Newman, Holger Prokisch, and Martin Hrabě de Angelis
DOI: 10.15252/emmm.202114397

Corresponding author: Valerie Gailus-Durner (gailus@helmholtz-muenchen.de)

Review Timeline:

Submission Date:	8th Apr 21
Editorial Decision:	11th May 21
Revision Received:	24th Aug 21
Editorial Decision:	13th Sep 21
Revision Received:	6th Oct 21
Accepted:	7th Oct 21

Editor: Zeljko Durdevic

Transaction Report:

(Note: With the exception of the correction of typographical or spelling errors that could be a source of ambiguity, letters and reports are not edited. Depending on transfer agreements, referee reports obtained elsewhere may or may not be included in this compilation. Referee reports are anonymous unless the Referee chooses to sign their reports.)

11th May 2021

Dear Dr. Gailus-Durner,

Thank you for the submission of your manuscript to EMBO Molecular Medicine. We have now received feedback from the three reviewers who agreed to evaluate your manuscript. As you will see from the reports below, the referees acknowledge the interest of the study but also raise important criticism that should be addressed in a major revision.

Further consideration of a revision that addresses reviewers' concerns in full will entail a second round of review. EMBO Molecular Medicine encourages a single round of revision only and therefore, acceptance or rejection of the manuscript will depend on the completeness of your responses included in the next, final version of the manuscript. For this reason, and to save you from any frustrations in the end, I would strongly advise against returning an incomplete revision.

We would welcome the submission of a revised version within three months for further consideration. However, we realize that the current situation is exceptional on the account of the COVID-19/SARS-CoV-2 pandemic. Please let us know if you require longer to complete the revision.

I look forward to receiving your revised manuscript.

Yours sincerely,

Zeljko Durdevic

Zeljko Durdevic
Editor
EMBO Molecular Medicine

***** Reviewer's comments *****

Referee #1 (Remarks for Author):

This study by Vidali and co-workers presents two patients (cousins) harbouring a novel homozygous two-exon deletion in the UQCRH gene. The UQCRH gene encodes a low MW subunit (Cytochrome b-c1 complex subunit 6) of the ubiquinol-cytochrome c reductase (complex III; CIII; or bc1 complex) of the electron transport chain (ETC). It appears that UQCRH "links" cytochrome c (CYCS) to cytochrome c1 (CYC1). Both patients presented with lactic acidosis, hyperammonaemia, hypoglycaemia and encephalopathy. A generally similar, though more severe, phenotype was observed in a gene deletion (KO) model (Uqcrh-/- mice) generated in this study. At the molecular level, patient and KO mouse tissues displayed a reduced CIII activity, a fully assembled CIII holoenzyme of reduced MW and increased levels of supercomplex CI2CIII2. Importantly, although only partially, complementation experiments rescued the phenotype in patient fibroblasts. Overall, this manuscript is well written and therefore easy to understand. The presented experimental data generally support the conclusions drawn and the complexome analysis is a valuable state-of-the-art addition. Generation and analysis of the Uqcrh-/- mouse model is also of substantial added value. However, besides the large body of solid data presented in this work, there are still some questions and concerns (see below), including the lack of an appropriate UQCRH-specific antibody for human samples. Therefore interpretation of some of the results is less straightforward.

MAJOR COMMENTS

1. In relation to Figure 2 and Table S1. Although food was available ad libitum was the food intake of Uqcrh-/- mice affected (also since moist and normal chow were used)? How does this relate to the "feeding pattern" of UQCRH patients?

2. Were gender-specific effects observed in Uqcrh-/- mice?

3. Given the increased sensitivity of mitochondrial patients and the Ndufs4-/- mouse model top anaesthetics. Did Uqcrh-/- mice display increased sensitive to isoflurane anaesthesia?

4. For Uqcrh-/- mice the humane endpoint was reached at the age of 12 weeks. How does this relate to the age and severity of the disease in UQCRH patients and/or other human patients with isolated CIII deficiency?

5. In relation to Figure 3A-B. Activities are presented normalized on CS. Although this is a standard approach in diagnostics, these values should also be presented after normalization on mg protein. Alternatively, evidence should be provided that the CS values are similar between samples.

6. Figure 3C-D. Please present less-cropped versions of the blots including MW markers. Alternatively, these could be part of the Supplementary Information. Moreover, although the Porin/VDAC1 antibody is mentioned in the M&M, this is not presented in Figure 3C-D. Please include these results.

7. Figure 3C. It is problematic that UQCRH cannot be detected by Western blotting. From the data it appears that this is due to the rabbit UQCRH polyclonal antibody (used in Figure 3E) not being compatible with human cells? Is there another human-compatible or monoclonal antibody available that can be used for Western blotting? Perhaps a Q-PCR analysis is useful? Or an immunofluorescence analysis of the human cells using rabbit UQCRH polyclonal antibody?

8. Figure 3D. Can the authors provide an estimate of (or speculate on) the MW drop for CIII in the patient cells? Given the fact that UQCRH has a predicted MW of ~11-kDa, would loss of this protein be detectable at all using BN-PAGE?

9. Figure 3F. Please include the VDAC1 data (or provide this information in the Supplement).

10. Is there any inhibitory effect of Tetracyline (Tet) treatment on mitochondrial function in experiments with the TetOne Inducible Expression System used for expression of WT UQCRH in the patient fibroblasts? Given the problems with detecting the UQCRH protein in this model, the authors could consider overexpressing a tagged variant of the UQCRH wild-type gene for complementation.

11. Regarding the immunohistochemistry in Figure 4, proper control experiments should be performed (i.e. how do the images look when only secondary antibodies are used for staining).

12. This study lacks an overall analysis of mitochondrial function (e.g. OCR, ECAR). Perhaps this can be performed in the UQCRH patient cells, and/or complemented cell system (see also the above remark on the potential Tet off-target effect).

13. Please describe in detail how images were quantified (e.g. Figure 3G, Figure 4I).

MINOR COMMENTS

1. For non-experts; what was the purpose of performing the H&E staining (page 12)?

2. What is "multi-organ decompensation" (page 13) and "metabolic decompensation" (page 16)?

3. Please state for all used antibodies if they are monoclonal or polyclonal.

Referee #2 (Remarks for Author):

Vidali et al. present a quite interesting study identifying and characterizing a novel homozygous deletion of exons 2 and 3 in the complex III structural subunit UQCRH in two human subjects a reproduce an homologs genetic alteration in mice. The parallel analysis of the human collected symptoms, human derive fibroblast, and mouse phenotyping led them to conclude:
- UQCRH is not required for assembly nor activity of CIII both in human and mice.
- Biochemical phenotype is similar between human cells and mice: partial reduction of CIII activity, reduction of the apparent size in BNGE of the dimmer CIII, increase of high molecular weight supercomplex composed by CI and CIII (named by the authors SXL
- Both human and mice manifest characteristic features of mitochondrial diseases by the disease is far more sever in the mouse (even fatal).
The major conclusions are sufficiently supported and of interest. This reviewer finds that the experimental effort done, particularly the complexome analysis, may allow a better consolidation and support of results and increase the significance of the conclusion. Besides, there are several omissions in the review of literature that may be relevant for the proper interpretation

of the results:
1) Page 7 second paragraph of introduction it is stated: "Indeed, deficiency of CIII (Protasoni et al, 2020) or CIV (Diaz et al, 2006) usually leads to impaired activity of CI." However, the first description showing the deficiency of CIII impact on CI was published in in 2004 (PMID: 15053874) and confirmed some months later by an independent group (PMID: 15053874). The quoted reference (Protasoni et al, 2020) is therefore incorrect.
2) The authors discuss their findings with respect to the other CIII mouse models in the literature, but they omit those affecting Rieske (PMID: 28504706; PMID: 18401429; PMID: 22106410).
3) Regarding SXL supercomplex: This supercomplex is not novel, it was described several times (PMID: 19026783; PMID: 27052170) were the presence of only CI and CIII was demonstrated. This reviewer agrees with the authors when suggesting that their electrophoretic mobility may be compatible with having two CI and a dimmer of CIII. However, nor the previous description in other papers nor the data provide in the current paper demonstrate that this is in fact a supercomplex with a dimmer of CIII attached by each protomer to a monomer CI. Very plausible alternative explanations are equally feasible, like a SC: I+III2 interaction with a yet unknown component in the mitochondrial inner membrane. This need to be clarify in the manuscript to prevent misunderstanding.
4) The fact that the mice present a much more severe phenotype than that describe in the human patients is of great interest and could demand more discussion on the nature of this differences i.e. it is expected that the same genetic defect in a different mouse strain would show a milder phenotype?
5) The complexome analysis may allow to answer a number of question of relevance as an example:
a. Is the relative distribution of CIII between free dimmer and associated with CI, CIV or both together altered?
b. Is the amount of CI in free vs superassembled form altered?
c. Is the proportion of CIV between different forms: monomer, dimmer, with CIII, or with CIII and CI modified?
d. Are the other isoforms of CIV affecting several subunits altered in expression?

Referee #3 (Remarks for Author):

Authors describe a single family with two affected individuals with two-exon deletion in UQCRH, which lead to a new and very rare CIII subunit abnormality. As they could not find any additional patients/families with UQCRH abnormality, they created and investigate a homozyogous Uqcrh two-exon deletion mice and compared them to the family with the two-exon deletion in UQCRH. Generally, their works are scientifically sound to this reviewer. This reviewer agrees with the difficulty to collect more patients/human samples in CIII defects. And human and mouse data mostly support the similar/same pathogenic consequences but with some difference, supporting that biallelic UQCRH/Uqcrh defects cause apparent morbid status in humans and mice. This reviewer suggests several points which may be able to improve this manuscript.

1. It would be better to describe clinical information of this family with two affected individuals more comprehensively including age information (e.g., about the initial onset, testing, and current ages), developmental milestones, and a systematic summary of the phenotypes (perhaps a new table, too).
2. Authors should describe the complete comparison of human and mouse UQCRH/Uqcrh regarding the number of amino acids and their identity as two-exon deletion may cause the final different effects if their structures are not completely similar.
3. Is it possible to perform RT-PCR to see the effect of two-exon deletion in cDNA?
4. Quantification of Western blot is better.
5. Page 6: "In vertebrates CIII comprises 11 subunits assembled with the support of at least four additional factors" and "Three genes ...... while the exact function of the other seven ...." They are inconsistent. Please clarify these.
6. Page 7: "Pathogenic variants in eleven have been reported in association with CIII deficiency. Among those, seven ....three........and two........" These are inconsistent. Please clarify these.
7. Page 9 line 7: "He then presented three or four times per year" should read "He then presented similar episodes three or four times per year".
8. Page 10: A 2.2-kb homozygous deletion is small in size. How did they identify this tiny deletion?
9. To show the human deletion by c.55-528_243+47, information of such genomic position should be added to Fig. 1B.
10. It is indeed apparent to see the difference of glucose level in patients and homozygous mice (slightly decreased in patients VS increased in homozygous mice). Does this reflect the episodic abnormal burden in patients (rather milder) versus the continuous abnormal burden in homozygous mice (severer)? Could you add a little comment on this?
11. In Table S1, the only comparison of WT-male and female-homozygous has been done. No other combination?
12. In Figure EV5, it is indeed difficult to see the fact that there is direct contact between UQCRH and complex I or complex IV. Where is UQRCH in Figure EV5.

EMM-2021-14397, Vidali *et al*.
Response Reviewers' Comments

Reviewer #1 (Remarks for Authors):
This study by Vidali and co-workers presents two patients (cousins) harbouring a novel homozygous two-exon deletion in the UQCRH gene. The UQCRH gene encodes a low MW subunit (Cytochrome b-c1 complex subunit 6) of the ubiquinol-cytochrome c reductase (complex III; CIII; or bc1 complex) of the electron transport chain (ETC). It appears that UQCRH "links" cytochrome c (CYCS) to cytochrome c1 (CYC1). Both patients presented with lactic acidosis, hyperammonaemia, hypoglycaemia and encephalopathy. A generally similar, though more severe, phenotype was observed in a gene deletion (KO) model (Uqcrh-/- mice) generated in this study. At the molecular level, patient and KO mouse tissues displayed a reduced CIII activity, a fully assembled CIII holoenzyme of reduced MW and increased levels of supercomplex CI2CIII2. Importantly, although only partially, complementation experiments rescued the phenotype in patient fibroblasts. Overall, this manuscript is well written and therefore easy to understand. The presented experimental data generally support the conclusions drawn and the complexome analysis is a valuable state-of-the-art addition. Generation and analysis of the Uqcrh-/- mouse model is also of substantial added value. However, besides the large body of solid data presented in this work, there are still some questions and concerns (see below), including the lack of an appropriate UQCRH-specific antibody for human samples. Therefore interpretation of some of the results is less straightforward.

Authors' Response: We would like to thank the reviewer for the comments regarding the writing of the manuscript and value of the complexome analysis and mouse work. We will endeavour to address each of the concerns listed below in turn.

Major Comments
1. "In relation to Figure 2 and Table S1. Although food was available ad libitum was the food intake of Uqcrh-/- mice affected (also since moist and normal chow were used)? How does this relate to the "feeding pattern" of UQCRH patients? "

Authors' Response: This is an excellent point. To avoid and exclude the possibility that the phenotype of the *Uqcrh*$^{-/-}$ mice (weakness and signs of fatigue) was a direct consequence of undernutrition we included readily accessible, moist food in the cage. The moist food was provided fresh twice a day in addition to the normal dry chow diet. Based on behavioural observation and daily health checks we did not observe any issues in food intake in the *Uqcrh*$^{-/-}$ mice, However, it was not possible to measure the food intake precisely (e.g. using indirect calorimetry) since our system only allows measurement with dry food. We have now specified this more clearly in the Methods section as described below:

Page 27 added text: "*Uqcrh*$^{-/-}$ homozygous mice and their wild-type controls were fed *ad libitum* with moist food provided fresh twice a day in addition to the normal chow food. Food intake was monitored during daily health checks and no issues were observed in food intake in the *Uqcrh*$^{-/-}$ mice."

Regarding the *UQCRH* patients, to the best of our knowledge, they do not follow any specific dietary regime and the parents have not reported any food preference or particular eating habits.

2. "Were gender-specific effects observed in Uqcrh-/- mice?"

Authors' Response: We did check for gender specific differences across the entire dataset during our analyses, but no differences were observed. Therefore, we present all data together since the phenotypes reported in the mouse concern both sexes.

We have now specified in the text as below:
Page 12: “Despite the body weight difference of male and female *Uqcrh*$^{-/-}$ mice, phenotypic data were compiled for both sexes and were independent from sex.”

3. *Given the increased sensitivity of mitochondrial patients and the Ndufs4-/- mouse model top anaesthetics. Did Uqcrh-/- mice display increased sensitivity to isoflurane anaesthesia?*

Authors’ Response: We anesthetised the mice only shortly before the killing, however we did not recognise a clear difference in *Uqcrh*$^{-/-}$ mice compared to controls in response to isoflurane.

Page 13: “No difference in response to isoflurane in Uqcrh-/- mice compared to controls was observed.”

4. *For Uqcrh-/- mice the humane endpoint was reached at the age of 12 weeks. How does this relate to the age and severity of the disease in UQCRH patients and/or other human patients with isolated CIII deficiency?*

Authors’ Response: In terms of average lifespan, 12 weeks of age in mice corresponds to approximately 20 years of age in humans. The last contact with patient II-1 and patient II-4 was at the age of 12 and 8 years respectively. Since then neither patient has visited the hospital and, according to reports from parents, they are considered healthy.
In relation to other patients with genetically-confirmed, isolated CIII deficiency, patients present with a wide range of clinical presentations with variable age of onset depending on the specific genetic defect, so it is more difficult to make clear comparisons.

5. *In relation to Figure 3A-B. Activities are presented normalized on CS. Although this is a standard approach in diagnostics, these values should also be presented after normalization on mg protein. Alternatively, evidence should be provided that the CS values are similar between samples.*

Authors’ Response: As the reviewer states, normalising to citrate synthase activity is the standard practice and provides a more accurate reflection of mitochondrial content, particularly for human samples in an accredited diagnostic laboratory setting. Indeed, this is how our patient data are presented and we are unable to provide quantified protein concentrations for the human samples. We can confirm that CS values were similar between samples and have all been checked to be within an established normal-range. However, we do have the data available for the mouse samples and we have now provided these data in the Appendix Fig S1A to show that there was no disparity between the CS activity and total protein content. These data are now referred to in the figure legends for Figure 3B.

6. *Figure 3C-D. Please present less-cropped versions of the blots including MW markers. Alternatively, these could be part of the Supplementary Information. Moreover, although the Porin/VDAC1 antibody is mentioned in the M&M, this is not presented in Figure 3C-D. Please include these results.*

Authors’ Response: We have now included the source data for the blots showing the uncropped original files that the figure was based on (Source data Figure 3 C-D). Porin/VDAC1 was not used on these blots, the total protein loading control in this case was alpha-tubulin.

7. *Figure 3C. It is problematic that UQCRH cannot be detected by Western blotting. From the data it appears that this is due to the rabbit UQCRH polyclonal antibody (used in Figure 3E) not being compatible with human cells?*

Authors' Response: We agree with the reviewer that the lack of a reliable UQCRH antibody in the human cells was problematic. We will address several of the reviewer's subsequent comments regarding this here rather than separately to try to make our approach more clear:

First, we do not know why the antibodies do not work well in human cells. We tried several commercially-available antibodies and several different transfer techniques for the western blots, but we were never able to obtain a strong or consistent signal in any human samples, including the controls.

Second, we did also try to make a FLAG-tagged version for the rescue experiments using the doxycycline inducible system, however we did not get any signal from an anti-FLAG antibody upon induction and we did not see any increase in UQCRC2 levels upon induction (unlike the untagged UQCRH, which did increase UQCRC2 levels). We therefore concluded that either the induction/transduction had not worked or that the FLAG tag was impairing function.

Third, to address this we extracted RNA from control cells, patient cells and the lentiviral transduced cells with either wt UQCRH or the FLAG tagged version (with uninduced and induced samples from each). We now provide these data below as part of our response:

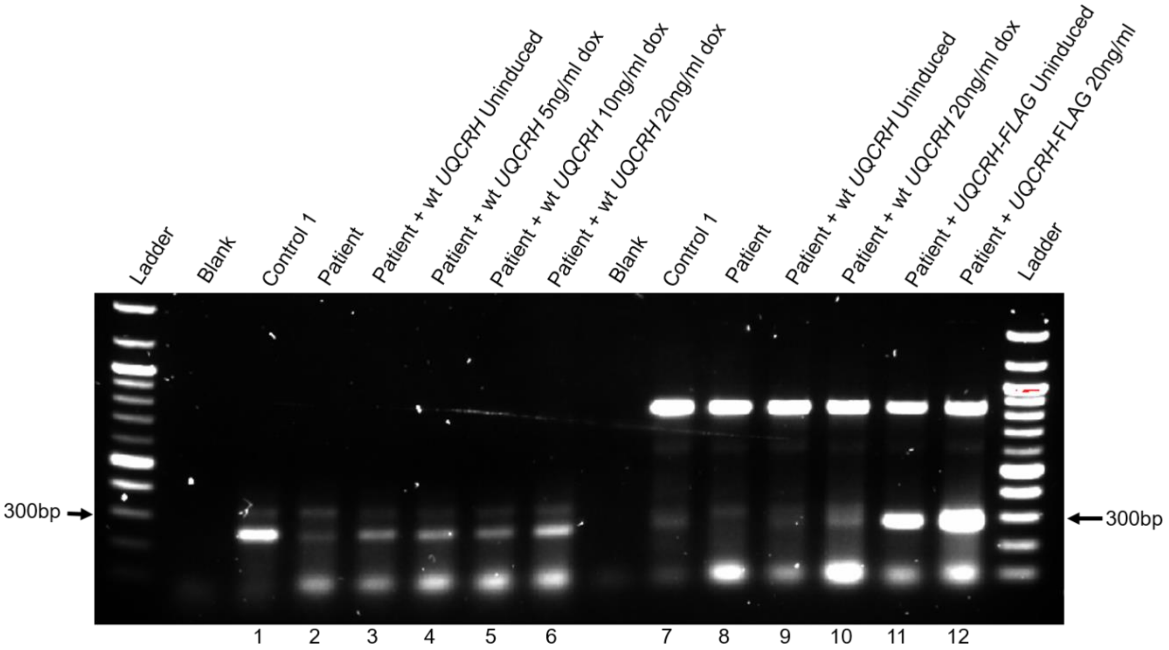

This was done using primers for wt UQCRH (lanes 1-6) and also using primers specific for the FLAG tagged version (lanes 7-12). We saw that there was an increase in wt *UQCRH* in the transduced cells regardless of induction status, suggesting that there was some leaky expression of the construct. However, it did appear that there was an increase above the 'leaky' levels when inducing the cells with 20ng/ml dox (lane 6). This did not seem to reach the levels from the control cells, which was in accordance with the western blot data for UQCRC2 levels. However when we compared to the FLAG tagged version using the FLAG specific reverse primer, we got very little signal from the control, the untransduced patient cells or the wt UQCRH transduced cells, but seemingly more 'leaky' expression and a further increased signal in the 20ng/ml dox induced cells transduced with FLAG-tagged UQCRH. It is not possible to say that the FLAG-tagged version was expressed more as different primers were used between the left (lane 1-6) and right (lane 7-12) half of the gel, but it does show that the transduction was successful. Since we could see some degree of mRNA expression from the FLAG-tagged UQCRH, but not detect the protein or any

increase in protein levels of UQCRC2, we concluded that it was likely that the FLAG tag interfered with function, so we did not pursue this further.

It is important to be clear that the data presented above were preliminary and the results were used to guide our further experiments, the RT-PCR was a single experiment which is why we did not include it in the main manuscript. We decided it seemed likely that the FLAG interfered with function and therefore we sought to improve and further validate the rescue only using wt UQCRH. The fact that we used a completely different viral vector system and a different technique (immunofluorescence vs western blot), but again demonstrated an increase in UQCRC2 (as a surrogate marker for CIII, in the absence of a good UQCRH antibody in human cells) in patient fibroblasts transduced with wt UQCRH strongly supports our assertions that these variants are pathogenic and that the lack of UQCRH is causing the CIII impairment in these cells.

Furthermore, although we do not have the antibody working in human cells to show absence of UQCRH protein, we do very clearly demonstrate an absence of UQCRH protein in the complexome data by mass spectrometry.

To come back to the original question, we certainly agree that it was problematic that the UQCRH antibodies did not work well in human cells, but we believe that all of the data we have provided are sufficient to consistently show that the patient cells lack UQCRH and that the introduction of a wt copy of UQCRH is able to ameliorate the biochemical (CIII) deficiency.

8. Is there another human-compatible or monoclonal antibody available that can be used for Western blotting?

Authors' Response: As above, we have tested several available antibodies and were unable to get any strong or consistent signal in human samples either by western blotting or immunofluorescence.

9. Perhaps a Q-PCR analysis is useful? Or an immunofluorescence analysis of the human cells using rabbit UQCRH polyclonal antibody?

Authors' Response: As for the western blots we did not have success with using UQCRH antibodies for immunofluorescence. We have not used Q-PCR, but have shown in preliminary data presented above that there was much less expression of *UQCRH* mRNA in the patient sample via RT-PCR. We stress that this was a single preliminary experiment and as such we cannot make any strong conclusions, however the observation of an apparent decrease in mRNA is consistent with the absence of UQCRH protein in the mass spectrometry data from our complexome experiments.

10. Figure 3D. Can the authors provide an estimate of (or speculate on) the MW drop for CIII in the patient cells? Given the fact that UQCRH has a predicted MW of ~11-kDa, would loss of this protein be detectable at all using BN-PAGE?

Authors' Response: Complex III is a stable dimer and has a calculated molecular weight of ~483 kDA (in both humans and mice). Our complexome data shows that we identified all subunits of complex III other than UQCRH (Fig. EV4AB). Only the smallest subunit UQCR11 escaped detection in human samples (control and patient, Fig. EV4A), but UQCR11 was detectable in mouse complex III at the level of supercomplexes in both wildtype and knockout samples (Fig. EV4B). Therefore, it seems the assembled complex III in cells/tissue with the 2 exon *UQCRH* deletion only lacks UQCRH itself. A complex III without UQCRH (mature mass of 9.2 kDa without mitochondrial targeting sequence or 18.4 kDa in the dimer) has a calculated mass of 465 kDa. With the resolution of blue native gels

a slide shift of the complex III band was visible from human mitochondria solubilized with dodecylmaltoside (Fig. 3D), from human samples solubilized digitonin (Fig. 5D, assigned as III, w/o QCR6) and in mouse samples (Fig. 5G, 5I assigned as III w/o QCR6). The estimated migration difference in our BN-gels would be approximately 0.6 %. The shift of the CIII band observed (e.g., Fig. 5G) was slightly more pronounced than expected (approx. 1.1 %) possibly indicating a structural change of the complex. This is even more visible in the complexomes where we detected complex III in mutants and patients one slice later (~1.7 %). We have now added the text below to the Discussion.
Page 22: "Furthermore, UQCRH/Uqcrh was the only CIII subunit that was not detected in assembled CIII in patient or Uqcrh-/- mice samples, but was detected in the respective controls (Fig EV4A-B). This suggests the ~1.1% shift in migration of assembled CIII was solely due to the absence of UQCRH/Uqcrh, despite the MW of UQCRH (18.4 kDa as mature form in dimer) only equating to ~0.6% of CIII (~483 kDa as dimer), perhaps due to a structural change in the complex."

11. *Figure 3F. Please include the VDAC1 data (or provide this information in the Supplement).*

Authors' Response: We have now provided representative images for VDAC1/Porin as Appendix Fig S1B and have referred to this in the legend of Fig 3F.

12. Is there any inhibitory effect of Tetracyline (Tet) treatment on mitochondrial function in experiments with the TetOne Inducible Expression System used for expression of WT UQCRH in the patient fibroblasts?

Author's Response: Though the system is named TetOne, it is actually doxycycline that is used for induction. We are aware that tetracycline and its derivatives can have a negative effect on mitochondrial translation, however, this has been shown at the 5-10µg/ml concentration range (Moullan et al 2015; PMID 25772356). We are only using up to 20ng/ml. We have done many experiments using this system with 200ng/ml doxycycline induction and have never seen any signs of impaired mitochondrial function in patients or controls, so there should be no effect in the data presented here.

13. Given the problems with detecting the UQCRH protein in this model, the authors could consider overexpressing a tagged variant of the UQCRH wild-type gene for complementation.

Authors' Response: We thank the reviewer for the suggestion, please refer to the answer to point 7 for extra detail, but we did try to use a C-terminal FLAG tagged version of UQCRH and our initial experiments suggested that this was not functional.

14. Regarding the immunohistochemistry in Figure 4, proper control experiments should be performed (i.e. how do the images look when only secondary antibodies are used for staining).

Authors' Response: We performed proper control experiments for immunohistochemistry, where we applied only secondary antibodies and we could not detect any background signal. However, unfortunately, we do not have such negative control images of the immunofluorescence saved to show.

15. *This study lacks an overall analysis of mitochondrial function (e.g. OCR, ECAR).*
OCR – lactate production in patients,
Perhaps this can be performed in the UQCRH patient cells, and/or complemented cell system (see also the above remark on the potential Tet off-target effect).

Authors' Response: Some initial experiments on the patient fibroblasts were performed using Seahorse technology, however these were preliminary and were not consistent. We have further studied the patient fibroblasts and mouse tissues using a variety of techniques that are much more informative and able to demonstrate a specific complex III phenotype that was common to both the human patients and the mouse model (e.g. complex activity data, BN-PAGE, complexome analysis). Thus, we feel our specific demonstration of CIII defects are sufficient to draw our conclusions without the measurement of downstream outputs of OCR or ECAR.

16. *Please describe in detail how images were quantified (e.g. Figure 3G, Figure 4I).*

Authors' Response: This information has now been added on page 33 of the M&M section:
"A scoring system was used to quantify the expression levels of UQCRC2 and VDAC1/Porin: 0= no staining; 1= weak staining; 2= moderate staining; 3= strong staining. The score for each section was calculated as the mean of 4 high-power fields (Vidali *et al*, 2017)."

MINOR COMMENTS

1. *For non-experts; what was the purpose of performing the H&E staining (page 12)?*

Authors' Response: Since functional analysis of the mouse heart revealed impaired conduction through the atrioventricular node and reduced stroke volume, we investigated further the heart morphology. The H&E did not reveal major differences. We have added H&E analysis of the heart of a representative *Uqcrh*$^{-/-}$ and WT mouse in the figure EV2 panel A where we highlight myocardium and aorta. Main text has also been adapted accordingly:

Pages 12-13: "Uqcrh-/- mice presented with the prolongation of the PR intervals and a decrease of left ventricular stroke volume, observed as early as 6 weeks of age (Fig 2G) indicating impaired conduction through the atrioventricular node. A microscopic investigation of the heart tissue from Uqcrh-/- mice by H&E staining, however, did not reveal any alterations compared to wild-type mice (Fig EV2A), but electron microscopy revealed mitochondrial paracrystalline inclusions in 5 of the 6 Uqcrh-/- hearts studied at 8 weeks of age (Fig EV2B)."

2. *What is "multi-organ decompensation" (page 13) and "metabolic decompensation" (page 16)?*

Authors' Response: For "multi-organ decompensation" we mean the failure of more than one organ to compensate for the functional overload resulting from the mitochondrial impairment (Piccinni P, et al., 2009). More precisely we observed liver, brain, kidney, heart and skeletal muscle impairment among others.

For "metabolic decompensation" we mean a derangement of normal metabolism reflected by disorders of biosynthesis and breakdown of complex molecules, such as carbohydrate, protein and lipids. Reflected by impaired glucose homeostasis, lactic acidosis and hyperammonaemia (Haijes HA, et al., 2020). The text has been adapted accordingly to make the comprehension easier (metabolic decompensation introduced in page 8; multi-organ decompensation page 13).

References:
Piccinni, P., et al. (2009). CHAPTER 56 - Multiple Organ Dysfunction Syndrome. Critical Care Nephrology (Second Edition). C. Ronco, R. Bellomo and J. A. Kellum. Philadelphia, W.B. Saunders: 309-312.

Haijes HA, Jans JJM, van der Ham M, van Hasselt PM, Verhoeven-Duif NM. Understanding acute metabolic decompensation in propionic and methylmalonic acidemias: a deep metabolic phenotyping approach. Orphanet J Rare Dis. 2020 Mar 6;15(1):68. doi: 10.1186/s13023-020-1347-3. PMID: 32143654; PMCID: PMC7060614.

3. *Please state for all used antibodies if they are monoclonal or polyclonal.*

Authors' Response: We have now included further information on the antibodies used in the Materials and Methods section (Page 30, paragraph: *SDS-PAGE, DDM solubilised BN-PAGE and Immunoblotting*). Additional details are provided in the accompanying Author Checklist file.

Referee #2 (Remarks for Author):

Vidali et al. present a quite interesting study identifying and characterizing a novel homozygous deletion of exons 2 and 3 in the complex III structural subunit UQCRH in two human subjects a reproduce an homologs genetic alteration in mice. The parallel analysis of the human collected symptoms, human derive fibroblast, and mouse phenotyping led them to conclude:

- UQCRH is not required for assembly nor activity of CIII both in human and mice.
- Biochemical phenotype is similar between human cells and mice: partial reduction of CIII activity, reduction of the apparent size in BNGE of the dimmer CIII, increase of high molecular weight supercomplex composed by CI and CIII (named by the authors SXL
- Both human and mice manifest characteristic features of mitochondrial diseases by the disease is far more sever in the mouse (even fatal).

The major conclusions are sufficiently supported and of interest. This reviewer finds that the experimental effort done, particularly the complexome analysis, may allow a better consolidation and support of results and increase the significance of the conclusion. Besides, there are several omissions in the review of literature that may be relevant for the proper interpretation of the results:

Authors' Response: We thank the reviewer for their comments and particularly the helpful suggestions on improvements we can make to our review of the literature, which we provide details of below. We are pleased the reviewer finds our conclusions to be sufficiently supported and interesting and we will attempt to address specific points below.

COMMENTS

1) Page 7 second paragraph of introduction it is stated: "Indeed, deficiency of CIII (Protasoni et al, 2020) or CIV (Diaz et al, 2006) usually leads to impaired activity of CI." However, the first description showing the deficiency of CIII impact on CI was published in in 2004 (PMID: 15053874) and confirmed some months later by an independent group (PMID: 15053874). The quoted reference (Protasoni et al, 2020) is therefore incorrect.

Authors' Response: We acknowledge that we did not use the earliest reference for showing a CIII defect impacting CI too. We were making a general point that this is often the case and the paper we cited is a recent publication that discusses several of these instances. Nevertheless, we agree that we should have included more of the earlier references individually and have now done so (page 7).

2) The authors discuss their findings with respect to the other CIII mouse models in the literature, but they omit those affecting Rieske (PMID: 28504706; PMID: 18401429; PMID: 22106410).

Authors' Response: We thank the reviewer for pointing this out. Our discussion mainly focused on full-body knockouts mice, that is why we omitted Rieske HSCs specific KO mouse model (PMID: 28504706) or in vitro induced KO (PMID: 22106410) thus representing conditions different from ours. It will be indeed interesting to check whether the absence of Uqcrh also influence haematopoiesis homeostasis or stem cells specific programs, however this is beyond the scope of this manuscript.

3) Regarding SXL supercomplex: This supercomplex is not novel, it was described several times (PMID: 19026783; PMID: 27052170) were the presence of only CI and CIII was demonstrated.

Authors' Response: We agree that the supercomplex that we see is not novel and we do not state that it is within the manuscript. Since more than twenty years, when Schägger and Pfeiffer published, that complex I, III and IV form stable stoichiometric associations in the inner mitochondrial membrane, we talk about supercomplexes or respirasomes (PMID: 10775262). Since that time, much research has been done to study the functional role of supercomplexes. The work of the research group of Jose Antonio Enriquez cited by reviewer 2 also discussed the physiological role of these associations (PMID: 19026783; PMID: 27052170). Having a closer look at the papers (PMID: 19026783; PMID: 27052170) they mainly discussed the supercomplexes (I_1-III_2-$IV_{0\text{-}4}$) with molecular masses from 1.5 MDa until around 2 MDa. However, there were bands of supercomplexes containing CI and III above 2 MDa, larger supercomplexes than the respirasome. Other reports characterized larger supercomplexes in potato mitochondria in BNE and cryo-EM with the stoichiometry (I_2-III_2, I_2-III_2-IV_2) (PMID: 19059196), and architecture of human megacomplex (I_2-III_2-IV_2) in cryo-EM (PMID: 28844695). Additional reports using blue native electrophoresis showed already higher ordered structures of the respirasomes were suggested that they could be organized in respiratory stings or patches (PMID: 20687061, PMID: 20687061). Therefore, there are additional reports that also identified complex IV in that megacomplex.

This reviewer agrees with the authors when suggesting that their electrophoretic mobility may be compatible with having two CI and a dimmer of CIII. However, nor the previous description in other papers nor the data provide in the current paper demonstrate that this is in fact a supercomplex with a dimmer of CIII attached by each protomer to a monomer CI. Very plausible alternative explanations are equally feasible, like a SC: I+III2 interaction with a yet unknown component in the mitochondrial inner membrane. This need to be clarify in the manuscript to prevent misunderstanding.

Authors' Response: We thank the reviewer for their comments and agree that with the size demonstrated on the electrophoresis it is compatible with having two CI and a dimer of CIII, but we do acknowledge that this is not conclusively demonstrated. We analysed respiratory chain complexes with BNE, Coomassie stain, in gel activity stains, western blots and complexome profiling. In our *Uqcrh*$^{-/-}$ mice and UQCRH patients we detected supercomplexes in a mass range between 1.5-2 MDa with an additional peak at 2.5 -3 MDa (human). 2.5 MDa corresponds very well to two assemblies of complex I (2x1MDa) and a dimer of complex III (1x 500 kDa) and the observed ~2.9 MDa size fits with an additional one or two copies of complex IV. The profile in Fig. 5E shows complex I (yellow) and III (red) as appearance at 2.5 MDa and also together with complex IV (green line) at 2.9 MDa. In the mouse complexome we detect similar patterns although extrapolation of native mass during calibration shows that this native gel was a bit more compressed in this high mass area. We inspected our complexome profiles again and could not detect any evidence of any other large complex, with an apparent mass of approximately 1 MDa, co-migrating

together with a supercomplex. Thus, we feel it is far less likely that there is an interaction with an as yet unknown component of a similar size to CI and more likely that it is indeed a second assembly of CI. As a human megacomplex with the stoichiometry (I_2III_2 and $I_2III_2IV_2$) has already been characterised in BNE and cryo-EM (PMID: 28844695) we concluded that we potentially isolated these complexes and called them extra-large supercomplexes (S_{XL}). However, other stoichiometries are not completely excluded. We have now modified the text as detailed below:

Page 23: “From the apparent mass of ~2.5 MDa, the composition of the subunits in our complexome profiling data (Fig EV4A) suggest that the most abundant giant supercomplex contains two CI connected via one dimer of CIII (Fig 5C-E, H-J), which is in accordance with a recently reported structure of human respiratory megacomplexes (Guo et al, 2017). We cannot rule out possible alternate stoichiometries in place of the second CI, although we could not identify any other mitochondrial complex of ~1 MDa, nor any homo- or hetero-multimer that would fit. Additionally, a larger complex of ~2.9 MDa also included CI and CIII as well as identification of CIV, which likely represents the megacomplex of two CI, one CIII dimer and either one or two CIV assemblies, as also reported in (Guo et al., 2017) (Fig 5E, J).”

4) The fact that the mice present a much more severe phenotype than that describe in the human patients is of great interest and could demand more discussion on the nature of this differences i.e. it is expected that the same genetic defect in a different mouse strain would show a milder phenotype?

Authors’ Response: This is an interesting question, and one that we cannot answer with any degree of certainty. It is certainly possible that carrying out the same experiments introducing the two-exon deletion of *Uqcrh* in mice with different genetic backgrounds could change the severity of the phenotype. There is actually some evidence for this in knockins of the *Bcs1l* gene. In a C57BL/6JCrlBomTac background Bcs1lc.A232G (Bcs1lp.S78G) knock-in mice bearing the GRACILE syndrome-analogous mutation there is a recapitulation many of the clinical manifestations and they also have a short survival of 35 days (Leéven et al, 2011 PMID: 21274865; Kotarsky et al, 2012 PMID: 22829922; Rajendran et al, 2016 PMID: 27809283; Purhonen et al, 2017 PMID: 28424480). However, in the slightly different C57BL/6JCrl substrain, the homozygotes develop the same early manifestations but do not succumb to the early metabolic crisis. This extends their survival to over 150 days (Purhonen et al, 2017 PMID: 28424480) and brings additional later-onset phenotypes, such as cerebral astrogliosis (Tegelberg et al, 2017 PMID: 28427446) and cardiomyopathy (Rajendran et al 2018 PMID 30530468). Furthermore, there can be huge variability in knockouts even in the same genetic background, for example knockouts of the mitochondrial protein SURF1 led high proportions of embryonic lethality, yet some of the mice survived beyond 12 months (Agostino et al. 2003, PMID: 12566387). Also a different SURF1 knockout did not display high levels of embryonic lethality and in fact had increased lifespan (Dell’Agnello et al 2007 PMID: 17210671) though this difference was attributed to a different cassette used for the knockout rather than different genetic backgrounds. In addition, for other diseases it is well known that different genetic backgrounds can have an impact on the phenotype, e.g. for amyotrophic lateral sclerosis (ALS) where backgrounds with different disease onsets are known, as well with different lifespan and affected organs (https://www.jax.org/news-and-insights/jax-blog/2014/november/same-mutation-different-phenotype#).

Generating different strains of mice and comparing the phenotypes would be interesting, but this was beyond the scope of the current manuscript. We feel that the key points are that the mouse model we present was viable as a whole-body knockout and furthermore displays markedly similar biochemical phenotypes to the human cases. Indeed, we have discussed the distinct possibility that the pseudogene present in humans may contribute to the less severe clinical phenotype compared to the mice that lack this, although we were

not able to provide direct supporting evidence for this theory. We have added the following text to the discussion:

Page 21: "Interestingly, there is some evidence that the genetic background of the Bcs1l mice affects the severity of the phenotype, with those from a C57BL/6JCrlBomTac having a short survival of 35 days (Kotarsky et al., 2012; Levéen et al., 2011; Purhonen et al, 2017; Rajendran et al, 2016), whereas in the slightly different C57BL/6JCrl background the homozygotes develop the same early manifestations but do not succumb to the early metabolic crisis, extending their survival to over 150 days (Purhonen et al., 2017; Rajendran et al., 2019; Tegelberg et al., 2017). It is possible that the severity of disease may be altered in different genetic backgrounds, which may be an area for future study in Uqcrh-/- mice."

5) The complexome analysis may allow to answer a number of question of relevance as an example:

a. Is the relative distribution of CIII between free dimmer and associated with CI, CIV or both together altered?

b. Is the amount of CI in free vs superassembled form altered?

c. Is the proportion of CIV between different forms: monomer, dimmer, with CIII, or with CIII and CI modified?

Authors' Response: We thank the reviewer for the further interest in the complexome data. We have now generated a new supplementary figure (new Fig. EV5) to answer the questions regarding relative distribution and added the following text to the Results section:

Pages 16-17: "We confirmed the appearance of assembled CIII with a loss of UQCRH in both the human patients and Uqcrh-/- mice although they were of lower abundance compared to the respective controls (Fig 5C, H; Fig EV4, Fig EV5). The amount of free complex IV was elevated in both the human patients (Fig EV5G) and Uqcrh-/- mice (Fig EV5J) possibly due to a compensatory effect of increased expression or due to there being less complex IV assembled into supercomplexes. Additionally, the relative abundance of supercomplexes in the mass range between 1.5 MDa and 2 MDa was lower compared to control mitochondria, suggesting that complex III lacking UQCRH assembles with the other respiratory complexes but to a lesser degree (Fig 5D,E; Fig EV5). The distribution of free complex I was unchanged between controls and Uqcrh-/- mice in mitochondrial samples (Fig EV5I)."

d. Are the other isoforms of CIV affecting several subunits altered in expression?

Authors' Response: We did not observe any other alterations to the composition of CIV other than the increased expression of Cox4i2.

Referee #3 (Remarks for Author):

Authors describe a single family with two affected individuals with two-exon deletion in UQCRH, which lead to a new and very rare CIII subunit abnormality. As they could not find any additional patients/families with UQCRH abnormality, they created and investigate a homozyogous Uqcrh two-exon deletion mice and compared them to the family with the two-exon deletion in UQCRH. Generally, their works are scientifically sound to this reviewer. This reviewer agrees with the difficulty to collect more patients/human samples in CIII

defects. And human and mouse data mostly support the similar/same pathogenic consequences but with some difference, supporting that biallelic UQCRH/Uqcrh defects cause apparent morbid status in humans and mice. This reviewer suggests several points which may be able to improve this manuscript.

Authors' Response: We thank the reviewer for their comments on our manuscript and are glad that they find our work scientifically sound. We will comment upon each of the suggested improvements point by point below:

1. It would be better to describe clinical information of this family with two affected individuals more comprehensively including age information (e.g., about the initial onset, testing, and current ages), developmental milestones, and a systematic summary of the phenotypes (perhaps a new table, too).

Authors' Response: We have now included a table in the appendix (also below) with all of the information we have available (Appendix Table S1). The patients do not show any other phenotypes except for those reported here and, as stated in the manuscript, the patients are not currently being seen in clinic and are considered to be healthy.

	**Individual II-1**	**Individual II-4**
Birth history	Birth at 38 weeks of gestation, normal pregnancy and delivery	Birth at 39 weeks of gestation, normal pregnancy and delivery
Initial presentation	2 years	3 years 4 months
Acute episode presentation	Vomiting, diarrhoea, confusion	Vomiting, diarrhoea, confusion
Biochemistry at acute presentation	raised plasma lactate<br>raised ammonia<br>hypoglycemia	raised plasma lactate<br>raised ammonia<br>hypoglycemia
Trigger for acute episode	mild unspecified viral illness	mild unspecified viral illness
Clinical examination findings	Left microtia, profound sensorineural and conductive hearing loss (complete occlusion of the ear canal and hypoplastic inner ear structures)	Nil of note
Growth	age 11.5 years: weight 38.6 kg (<75th centile) height 133 cm (<9th centile)	age 8 years: weight 25.2 kg (<50th centile) height 120 cm (<9th centile)
Development	Mild speech delay noted at age 3yr (likely secondary to hearing impairment) otherwise normal	Normal

Investigations (additional)	CT head, MR brain, echocardiogram all normal	MR brain normal
Last follow up	12 years	8 years

2. Authors should describe the complete comparison of human and mouse UQCRH/Uqcrh regarding the number of amino acids and their identity as two-exon deletion may cause the final different effects if their structures are not completely similar.

Authors' Response: The information on the amino acids that are deleted and the genetic identity between the human and mouse proteins (including the amino acid sequence that may be expressed from the human pseudogene *UQCRHL*) are presented in Figure 1D. We have now altered the text to make this clearer. Page 11: "A comparison of the deletion between the human and mouse protein is shown in Fig 1D."

3. Is it possible to perform RT-PCR to see the effect of two-exon deletion in cDNA?

Authors' Response: Within the answer to point 7 from Reviewer 1 we show some of our preliminary data where we used RT-PCR to assess the expression of wt-UQCRH that we used in our lentiviral system to try to rescue the phenotype. In this case we saw decreased signal in the patient sample, but did not see a smaller species equivalent to UQCRH missing 2 exons, perhaps suggesting that this mRNA is unstable and degraded.

4. Quantification of Western blot is better.

Authors' Response: We respectfully disagree with the reviewer that quantifying the blots is better. Western blotting is only a semi-quantitative technique and so absolute quantification is not possible. In the cases of the western blots that we present, the differences we comment on are very clear from the representative image and we do not feel would be enhanced by quantifying. We do show quantification for the immunofluorescence which is more accurate to demonstrate the increase of UQCRC2 in the patient fibroblasts transduced with wild-type *UQCRH*. We also have the mass spectrometry data from the complexome analysis available to access in the PRIDE database, which provides far better quantified data for each of the proteins. The western blots presented here allow an easily visible guide to the phenotype and we have now included the source data for these blots.

5. Page 6: "In vertebrates CIII comprises 11 subunits assembled with the support of at least four additional factors" and "Three genes ...... while the exact function of the other seven ...." They are inconsistent. Please clarify these.

Authors' Response: We thank the reviewer for spotting this, we have now changed the text on page 6 to: "the other eight (UQCRC1, UQCRC2, UQCRH, UQCRB, UQCRQ, Cytochrome b-c1 complex subunit 9, UQCR10 and UQCR11) remains to be fully elucidated (Fernández-Vizarra & Zeviani, 2015)".

6. Page 7: "Pathogenic variants in eleven have been reported in association with CIII deficiency. Among those, seven ....three........and two........" These are inconsistent. Please clarify these.

Authors' Response: Again, we thank the reviewer for spotting this error and apologise for the oversight, this has now been rectified and changed on page 7 to: "Among those, five encode subunits of CIII (Barel et al, 2008; Feichtinger et al., 2017a; Gaignard et al, 2013; Gasparre et al, 2008; Haut et al, 2003; Miyake et al, 2013), three encode CIII assembly

factors (Feichtinger et al., 2017a; Koch et al., 2015; Tucker et al., 2013; Wanschers et al., 2014) and three encode factors involved in the loading of iron sulphur clusters (2Fe-2S) to the Rieske protein (Gusic et al, 2020; Invernizzi et al, 2013; Kremer et al, 2016; Moran et al, 2010)".

7. Page 9: "He then presented three or four times per year" should read "He then presented similar episodes three or four times per year".

Authors' Response: We indeed mean that the patient presented similar episodes three-four times per year. The text has been changed accordingly. Page 9: "He then presented similar episodes three or four times per year".

8. Page 10: A 2.2-kb homozygous deletion is small in size. How did they identify this tiny deletion?

Authors' Response: Whole exome sequencing was performed on these samples and in both patients there were no reads for the part of the gene that was deleted. The text in "Molecular genetic investigations paragraph" on page 10 reads: "However, WES coverage data (Fig EV1B) indicated that both affected individuals carried a 2.2 kb homozygous deletion of exons 2 and 3 of UQCRH chr1:g.46,774,245-46,776,461 (**Fig 1B**). This was supported by review of the SNP array data, which revealed an absence of signal from the single probe within this region (**Fig EV1C**)".

9. To show the human deletion by c.55-528_243+47, information of such genomic position should be added to Fig. 1B.

Authors' Response: Thank you, this has now been added to the figure.

10. It is indeed apparent to see the difference of glucose level in patients and homozygous mice (slightly decreased in patients VS increased in homozygous mice). Does this reflect the episodic abnormal burden in patients (rather milder) versus the continuous abnormal burden in homozygous mice (severer)? Could you add a little comment on this?

Authors' Response: This is an interesting question, one of the most striking differences observed between mouse and human is indeed episodes of hypoglycaemia observed in patients versus the constant hyperglycaemia of the mouse. It is important to highlight that *Uqcrh*$^{-/-}$ mice are initially hypoglycaemic at an early post-weaning stage before becoming diabetics. Compared to patients, who are treated and recover after the recurrent episodes of hypoglycaemia, *Uqcrh*$^{-/-}$ mice are not treated and therefore normal glucose levels are not restored. Hypoglycaemia, therefore, appears to be the primary symptom of oxidative phosphorylation deficiency, in line with a reduction in ATP or NAD and FAD cofactors usually observed in respiratory chain defects, interfering with glucose homeostasis in several ways. We observed that the hypoglycaemic burden on young mice develops to hyperglycaemia in the long run. Whether this is an adaptation mechanism in the mouse is yet unknown.
We now discuss this point on page 21: "The contrast between episodes of hypoglycaemia observed in patients versus the hypoglycemia developing gradually to constant hyperglycaemia in the Uqcrh-/- mice was among the most striking difference observed. While the patients were treated and recovered from their recurrent episodes of hypoglycaemia, Uqcrh-/- mice were not treated, therefore normal glucose levels were not restored. Thus, hypoglycaemia appears to be the primary response to the OXPHOS deficiency. This is consistent with impaired ATP or cofactor (NAD and FAD) levels, which are usually observed in respiratory chain defects, impacting upon glucose homeostasis in several ways. We observed that when not treated, the hypoglycaemic burden on the mice developed into persistent hyperglycaemia. Whether this is an adaptation mechanism of the mouse is as yet unknown."

11. In Table S1, the only comparison of WT-male and female-homozygous has been done. No other combination?

Authors' Response: We thank the reviewer for noticing this point, due to a shift some parts of the text were lost. The Appendix table S1 has been fixed and updated as Appendix Table S2.

Appendix Table S2

Summary of clinical chemistry parameters measured in plasma of 8–12-week-old male and female *Uqcrh*$^{-/-}$ and wild-type controls under standard ad libitum fed conditions. Data are shown as median, quartiles and p-values of a Wilcoxon test.

	**Male**		**Female**		**Male**	**Female**	**Overall**
	WT	*Uqcrh*$^{-/-}$	WT	*Uqcrh*$^{-/-}$			
	n=8	n=7	n=7	n=9			
	Median [25%, 75%]	Median [25%, 75%]	Median [25%, 75%]	Median [25%, 75%]	p-value	p-value	p-value
**Potassium [mmol/L]**	4.1 [4, 4.1]	5.3 [5.1, 5.8]	5 [4.8, 5.3]	6.5 [5.4, 6.9]	< 0.001	0.003	< 0.001
**Total protein [g/L]**	48.2 [47.1, 50.6]	37.9 [36.5, 43.5]	47.5 [46.5, 48.8]	38.7 [37.2, 41]	< 0.001	< 0.001	< 0.001
**Albumin [g/L]**	26.4 [25.9, 27.8]	19.2 [17.8, 22.1]	24.4 [23.6, 24.6]	21.5 [18, 23.5]	< 0.001	0.006	< 0.001
**ALAT/GPT [U/L]**	24 [20, 31]	109 [100, 120]	22 [20, 26]	146 [93, 159]	< 0.001	< 0.001	< 0.001
**ASAT/GOT [U/L]**	66 [62, 80]	143 [124, 258]	66 [54, 82]	279 [173, 333]	< 0.001	< 0.001	< 0.001
**Glucose [mmol/L]**	15.54 [14.98, 16.27]	64.17 [61.95, 66.13]	17.1 [14.57, 20.93]	63.8 [60.63, 68.06]	< 0.001	< 0.001	< 0.001
**ALP [U/L]**	164 [152, 167]	268 [232, 287]	94 [92, 101]	300 [256, 342]	< 0.001	< 0.001	< 0.001
**Total iron binding capacity [µmol/L]**	55.9 [54.3, 60.8]	41.7[a] [39.1, 42.7]	59.1 [56.9, 61.4]	36.8[a] [35.7, 37.4]	0.001	0.003	< 0.001

[a] Number not based on the full number of animals: 6 female and 5 male *Uqcrh*$^{-/-}$.

12. In Figure EV5, it is indeed difficult to see the fact that there is direct contact between UQCRH and complex I or complex IV. Where is UQRCH in Figure EV5.

Authors' Response: UQCRH was labelled in the figure under its other protein name of QCR6. We have now added the labelling of UQCRH to make this clearer. Though this is now Appendix Fig S2 as we have added a new figure that is now Fig EV5.

13th Sep 2021

Dear Dr. Gailus-Durner,

Thank you for the submission of your revised manuscript to EMBO Molecular Medicine. I am pleased to inform you that we will be able to accept your manuscript pending the following final amendments:

1) Please address all the points raised by the referee #3.
2) In the main manuscript file, please do the following:
- Add up to 5 keywords.
- In M&M, include a statement that informed consent from patients was obtained and that the experiments conformed to the principles set out in the WMA Declaration of Helsinki and the Department of Health and Human Services Belmont Report.
- In M&M, a statistical paragraph that should reflect all information that you have filled in the Authors Checklist, especially regarding randomization, blinding, replication.
- Please be aware that all deposited data have to be freely accessible before publication of the manuscript.

Please check "Author Guidelines" for more information.
https://www.embopress.org/page/journal/17574684/authorguide#availabilityofpublishedmaterial
3) Table 1: Remove "data not shown".
4) Synopsis: Every published paper now includes a 'Synopsis' to further enhance discoverability. Synopses are displayed on the journal webpage and are freely accessible to all readers. They include separate synopsis image and synopsis text.
- Synopsis image: Please provide a striking image or visual abstract as a high-resolution jpeg file 550 px-wide x (250-400)-px high to illustrate your article.
- Synopsis text: Please provide a short standfirst (maximum of 300 characters, including space) as well as 2-5 one sentence bullet points that summarise the paper as a .doc file. Please write the bullet points to summarise the key NEW findings. They should be designed to be complementary to the abstract - i.e. not repeat the same text. We encourage inclusion of key acronyms and quantitative information (maximum of 30 words / bullet point). Please use the passive voice.
- Please check your synopsis text and image before submission with your revised manuscript. Please be aware that in the proof stage minor corrections only are allowed (e.g., typos).
5) For more information: There is space at the end of each article to list relevant web links for further consultation by our readers. Could you identify some relevant ones and provide such information as well? Some examples are patient associations, relevant databases, OMIM/proteins/genes links, author's websites, etc...
6) As part of the EMBO Publications transparent editorial process initiative (see our Editorial at http://embomolmed.embopress.org/content/2/9/329), EMBO Molecular Medicine will publish online a Review Process File (RPF) to accompany accepted manuscripts. This file will be published in conjunction with your paper and will include the anonymous referee reports, your point-by-point response and all pertinent correspondence relating to the manuscript. Let us know whether you agree with the publication of the RPF and as here, if you want to remove or not any figures from it prior to publication. Please note that the Authors checklist will be published at the end of the RPF.
7) Please provide a point-by-point letter INCLUDING my comments as well as the reviewer's reports and your detailed responses (as Word file).

I look forward to reading a new revised version of your manuscript as soon as possible.

Yours sincerely,

Zeljko Durdevic

***** Reviewer's comments *****

Referee #2 (Remarks for Author):

The authors have answered all my concerns properly

Referee #3 (Remarks for Author):

Authors responded well to the most of this reviewer's concerns in the revised manuscript with reasonable data and discussion. This reviewer still requests several corrections, but all minor.

1. Page 10 lines 201 -202: Homozygous variants in two genes could be as a potential interest to readers. For clarity, the authors should provide how to filter them out as a culprit and detailed information of variants (e.g. gnomad registration and their frequency and in-silico prediction of pathogenicity).
2. Page 10, line 204: Information of reference genome used should be provided to genomic position such as GRCh37 or GRCh38.
3. Figure 1A: Pedigree information should be revised with more familial members and generation number such as I, II, etc.
4. Figure 1B: Information of genomic position should be provided together with c.55-528_243+47. Nucleotide information above the electropherogram should be improved with information of genomic position.
5. Page 10, line 208: "a shortened product" may be appropriate rather than "a truncated product".
6. Page 11, line 221: "in humans that is absent in mice" should read "in humans, that is absent in mice".
7. Page 13, "the PR intervals" should read "the PR intervals in electrocardiogram".
8. Page 14, line 294: "a truncated version" should read "a shortened version".

******* Reviewer's comments *******

Referee #2 (Remarks for Author):

The authors have answered all my concerns properly
Authors' response: We thank the reviewer for taking the time to review our revised manuscript and are pleased to have addressed all of their concerns.

Referee #3 (Remarks for Author):

Authors responded well to the most of this reviewer's concerns in the revised manuscript with reasonable data and discussion. This reviewer still requests several corrections, but all minor.
Authors' response: We are glad to have addressed most of the reviewers' concerns and will endeavour to correct the remaining minor points in turn below.

1. Page 10 lines 201 -202: Homozygous variants in two genes could be as a potential interest to readers. For clarity, the authors should provide how to filter them out as a culprit and detailed information of variants (e.g. gnomad registration and their frequency and in-silico prediction of pathogenicity).
Authors' response: We did indeed look at the prevalence of the variants in gnomAD with the *HIVEP3* c.7043C>T; p.Pro2348Leu variant being present in 130 out of 181612 alleles including 1 homozygote and the *TCTEX1D4* c.140C>T; p.Pro47Leu variant present in 1679 alleles out of 128458 including 19 homozygotes. The presence of these variants in control populations, as well as the variant predicted as benign, using *in silico* tools did not suggest pathogenicity. Also, there was no supporting evidence for either of these variants to be expected to cause a CIII deficiency. Therefore, we pursued the subsequently identified

UQCRH deletion and then provided evidence of pathogenicity with the biochemical studies in patient fibroblasts (including the amelioration of CIII deficiency with the lentiviral rescue experiments) and in the mouse model that showed very similar biochemical phenotypes.

We have added the following text to the manuscript to reflect this:
Line 201_205: "Neither of these variants were likely pathogenic due to presence in control populations (in gnomAD the *HIVEP3* variant was present in 130 out of 181612 alleles including 1 homozygote and the *TCTEX1D4* variant present in 1679 alleles out of 128458 including 19 homozygotes) and were predicted benign using *in silico* tools."

2. Page 10, line 204: Information of reference genome used should be provided to genomic position such as GRCh37 or GRCh38.
Authors' response: We used GRCh7/hg19 assembly to define the original genomic position of the deletion based on the array and exome data. This information has been now added to the text.

3. Figure 1A: Pedigree information should be revised with more familial members and generation number such as I, II, etc.
Authors' response: We have now added improved generation numbers to the pedigree in Figure 1, however, we do not have information as to additional family members.

4. Figure 1B: Information of genomic position should be provided together with c.55-528_243+47. Nucleotide information above the electropherogram should be improved with information of genomic position.
Authors' response: We defined the specific breakpoints of the deletion by PCR across the break and Sanger sequencing and numbered according to NM_006004.4 (c.55-528_243+47) and the genomic co-ordinates hg38 g.46308573_46310363del. This information has been added to Figure 1 panel B.

5. Page 10, line 208: "a shortened product" may be appropriate rather than "a truncated product".
Authors' response: This has now been changed as suggested.

6. Page 11, line 221: "in humans that is absent in mice" should read "in humans, that is absent in mice".
Authors' response: This has now been changed to "in humans, which is absent in mice".

7. Page 13, "the PR intervals" should read "the PR intervals in electrocardiogram".
This has been changed
Authors' response: This has now been changed as suggested.

8. Page 14, line 294: "a truncated version" should read "a shortened version".
This has been changed
Authors' response: This has now been changed as suggested.

2nd Revision - Editorial Decision 7th Oct 2021

We are pleased to inform you that your manuscript is accepted for publication and is now being sent to our publisher to be included in the next available issue of EMBO Molecular Medicine.

EMBO PRESS

YOU MUST COMPLETE ALL CELLS WITH A PINK BACKGROUND ➔

PLEASE NOTE THAT THIS CHECKLIST WILL BE PUBLISHED ALONGSIDE YOUR

USEFUL LINKS FOR COMPLETING THIS FORM

Corresponding Author Name: Valerie Gailus-Durner

Journal Submitted to: EMBO Molecular Medicine
Manuscript Number: EMM-2021-14397

http://www.antibodypedia.com	Antibodypedia
http://1degreebio.org	1DegreeBio
http://www.equator-network.org/reporting-guidelines/improving-bioscience-res	ARRIVE Guidelines
http://grants.nih.gov/grants/olaw/olaw.htm	NIH Guidelines in animal use
http://www.mrc.ac.uk/Ourresearch/Ethicsresearchguidance/Useofanimals/inde	MRC Guidelines on animal use
http://ClinicalTrials.gov	Clinical Trial registration
http://www.consort-statement.org	CONSORT Flow Diagram
http://www.consort-statement.org/checklists/view/32-consort/66-title	CONSORT Check List
http://www.equator-network.org/reporting-guidelines/reporting-recommendatio	REMARK Reporting Guidelines (marker prognostic studies)
http://datadryad.org	Dryad
http://figshare.com	Figshare
http://www.ncbi.nlm.nih.gov/gap	dbGAP
http://www.ebi.ac.uk/ega	EGA
http://biomodels.net/	Biomodels Database
http://biomodels.net/miriam/	MIRIAM Guidelines
http://jjj.biochem.sun.ac.za	JWS Online
http://oba.od.nih.gov/biosecurity/biosecurity_documents.html	Biosecurity Documents from NIH
http://www.selectagents.gov/	List of Select Agents

Reporting Checklist For Life Sciences Articles (Rev. June 2017)

This checklist is used to ensure good reporting standards and to improve the reproducibility of published results. These guidelines are consistent with the Principles and Guidelines for Reporting Preclinical Research issued by the NIH in 2014. Please follow the journal's authorship guidelines in preparing your manuscript.

A- Figures
1. Data
The data shown in figures should satisfy the following conditions:

- the data were obtained and processed according to the field's best practice and are presented to reflect the results of the experiments in an accurate and unbiased manner.
- figure panels include only data points, measurements or observations that can be compared to each other in a scientifically meaningful way.
- graphs include clearly labeled error bars for independent experiments and sample sizes. Unless justified, error bars should not be shown for technical replicates.
- if n< 5, the individual data points from each experiment should be plotted and any statistical test employed should be justified
- Source Data should be included to report the data underlying graphs. Please follow the guidelines set out in the author ship guidelines on Data Presentation.

2. Captions
Each figure caption should contain the following information, for each panel where they are relevant:

- a specification of the experimental system investigated (eg cell line, species name).
- the assay(s) and method(s) used to carry out the reported observations and measurements
- an explicit mention of the biological and chemical entity(ies) that are being measured.
- an explicit mention of the biological and chemical entity(ies) that are altered/varied/perturbed in a controlled manner.
- the exact sample size (n) for each experimental group/condition, given as a number, not a range;
- a description of the sample collection allowing the reader to understand whether the samples represent technical or biological replicates (including how many animals, litters, cultures, etc.).
- a statement of how many times the experiment shown was independently replicated in the laboratory.
- definitions of statistical methods and measures:
  - common tests, such as t-test (please specify whether paired vs. unpaired), simple χ2 tests, Wilcoxon and Mann-
  - are tests one-sided or two-sided?
  - are there adjustments for multiple comparisons?
  - exact statistical test results, e.g., P values = x but not P values < x;
  - definition of 'center values' as median or average;
  - definition of error bars as s.d. or s.e.m.

Any descriptions too long for the figure legend should be included in the methods section and/or with the source data.

In the pink boxes below, please ensure that the answers to the following questions are reported in the manuscript itself. Every question should be answered. If the question is not relevant to your research, please write NA (non applicable).
We encourage you to include a specific subsection in the methods section for statistics, reagents, animal models

B- Statistics and general methods

Please fill out these boxes ➔ (Do not worry if you cannot see all your text once you press re

1.a. How was the sample size chosen to ensure adequate power to detect a pre-specified effect size?	No statistical method was applied to choose the sample size. Sample size was chosen based on experience for the various experiments performed.
1.b. For animal studies, include a statement about sample size estimate even if no statistical methods were used.	Sample size estimation is based on long term experience in high-throughput phenotyping. The selected sample size of 15 mice per sex and genotype is sufficient to find a medium difference of one standard deviation with a power of 0.8 and alpha of 0.05. For some parameters (e.g categorical, yes/no) even less animals are sufficient (Pages 35/36).
2. Describe inclusion/exclusion criteria if samples or animals were excluded from the analysis. Were the criteria pre-established?	In general, no data point is excluded from the analysis unless there is a clear evidence of technical problem, i.e.., technical failure of the experimental machine. For many of the parameters (i.e., blood parameters) we have pre-established threshold values as indicators for invalid measurements. If the data point reaches the criteria we replace the data point by status codes(i.e., above upper limit) (Page 35).
3. Were any steps taken to minimize the effects of subjective bias when allocating animals/samples to treatment (e.g. randomization procedure)? If yes, please describe.	Groups are assigned according to the genotype and sex of the animals (Page 27). Immunohistochemistry was performed and photos were taken in blinded conditions (Pages 34/35).
For animal studies, include a statement about randomization even if no randomization was used.	Control and mutant mice were alternatively measured in order to remove biases from the measurement and analysis processes (Page 27).
4.a. Were any steps taken to minimize the effects of subjective bias during group allocation or/and when assessing results (e.g. blinding of the investigator)? If yes please describe.	In most tests, experimenter were not blinded, as the experimenter has no influence on the results of these tests since directly recorded by the machine. In the tests, where the experimter might have an influence on the measurements, the experiment is conducted in blinded conditions, and we have SOP's prescribing how this will be ensured. In addition, we record plenty of metadata for each data point and monitor the influence of these metadata over time (Page 27).Image acquisition and analysis of immunohistochemestry of mice samples, were performed in blinded conditions (Page 34/35).
4.b. For animal studies, include a statement about blinding even if no blinding was done	Please see above
5. For every figure, are statistical tests justified as appropriate?	Since we have phenotyping data of control animals for more than 15 years, we know the distribution of every single parameter. Based on this knowledge, we selected the best statistical test. We have a customized analysis for each parameter including also complex models (eg. LME's, Bodyweight as additional covariate) (Pages 35/36).
Do the data meet the assumptions of the tests (e.g., normal distribution)? Describe any methods used to assess it.	Please see above. Distributon of the parameters are regularly checked. We implemented different quality control plots like QQ plots, histograms, KS test, effect sizes, etc., in our automated R-scripts used to analyse the data.(Pages 35/36).
Is there an estimate of variation within each group of data?	For each parameter, we calculate measures like mean, SD, effect size and respectively median and IQR in our standardized automatic R-scripts (Pages 35/36). For immunohistochemestry and activity of mice samples mean and SEM was calculated (Page 36).
Is the variance similar between the groups that are being statistically compared?	Please see above. We check variances for all groups of the parameters of interest and select the best statistical test according to the results.

C- Reagents

6. To show that antibodies were profiled for use in the system under study (assay and species), provide a citation, catalog number and/or clone number, supplementary information or reference to an antibody validation profile. e.g., Antibodypedia (see link list at top right), 1DegreeBio (see link list at top right).	Mouse monoclonal [20E9DH10C12] to NDUFB8 [Abcam ab110242]; Suitable for: WB, IHC-Fr; Reacts with: Mouse, Rat, Cow, Human. Mouse monoclonal [2E3GC12FB2AE2] to SDHA [Abcam ab14715]; Suitable for: IHC-Fr, Flow Cyt, WB, ICC, IHC-P; Reacts with: Mouse, Rat, Cow, Human. Mouse monoclonal [13G12AF12BB11] to UQCRC2 [Abcam ab14745]; Suitable for: Flow Cyt, IHC-P, WB; Reacts with: Human. Mouse monoclonal [1D6E1A8] to MT-CO1 [Abcam ab14705]; Suitable for: IHC-P, WB, Flow Cyt; Reacts with: Mouse, Rat, Cow, Human. Mouse monoclonal [15H4C4] to ATP5A - Mitochondrial Marker ATP5A [Abcam ab14748]; Suitable for: WB, IHC-P, ICC/IF, Flow Cyt; Reacts with: Mouse, Rat, Cow, Human, Drosophila melanogaster. Mouse monoclonal [20B12AF2] to VDAC1/Porin [Abcam ab14734]; Suitable for: WB, ICC/IF, Flow Cyt; Reacts with: Mouse, Rat, Cow, Human. Mouse monoclonal [DM1A] to alpha Tubulin - Loading Control [Abcam ab7291]; Suitable for: Flow Cyt, ICC/IF, IHC-P, WB; Reacts with: Mouse, Rat, Human. Rabbit monoclonal [EPR9039(B)] to UQCRH [Abcam ab134949] Suitable for: WB, IHC-P; Reacts with: Human. Mouse monoclonal [5] to VCP [Abcam ab11433] Suitable for: IHC-P, IHC-Fr, Flow Cyt, ELISA, ICC, IP, ICC/IF, WB; Reacts with: Mouse, Rat, Sheep, Cow, Human. Working dilutions of antibodies used for immunoblotting are indicated in pages 31/32, working dilutions of antibodies used for immunohistochemistry are indicated in pages 33/34.
7. Identify the source of cell lines and report if they were recently authenticated (e.g., by STR profiling) and tested for mycoplasma contamination.	The patients cell lines were derived from one of the two patients.NHDF were purchased from Lonza (#CC-2509).Cells were regularly tested for mycoplasma, all cells used in the experiments were tested mycoplasma negative (Pages 25, 33).

* for all hyperlinks, please see the table at the top right of the document

D- Animal Models

8. Report species, strain, gender, age of animals and genetic modification status where applicable. Please detail housing and husbandry conditions and the source of animals.	Mouse Model: Uqcrh knock-out (-/-) mice (C57BL/6NCrl-Uqcrhtm1b(EUCOMM)Wtsi/leg); 2 Exon deletion, details s. p24; Fig1; males and females were analyzed; mice were maintained in IVC cages with water and standard mouse chow according to the directive 2010/63/EU, German laws and GMC housing conditions (www.mouseclinic.de); Data comprise mouse age 1-10 weeks (Page 27).
9. For experiments involving live vertebrates, include a statement of compliance with ethical regulations and identify the committee(s) approving the experiments.	All tests were approved by the responsible authority of the district government of Upper Bavaria (Page 27).
10. We recommend consulting the ARRIVE guidelines (see link list at top right) (PLoS Biol. 8(6), e1000412, 2010) to ensure that other relevant aspects of animal studies are adequately reported. See author guidelines, under 'Reporting Guidelines'. See also: NIH (see link list at top right) and MRC (see link list at top right) recommendations. Please confirm compliance.	Our work complies where applicable to ARRIVE guidelines.

E- Human Subjects

11. Identify the committee(s) approving the study protocol.	The parents of the recruited children provided informed written consent for their participation in a study approved by South Manchester Ethics committee (11/H1003/3, IRAS 64321) and the University of Manchester to determine the genetic cause of the condition affecting their children (Page 25).
12. Include a statement confirming that informed consent was obtained from all subjects and that the experiments conformed to the principles set out in the WMA Declaration of Helsinki and the Department of Health and Human Services Belmont Report.	Yes - this is contained within the Materials and Methods, Ethics statement section (Page 25).
13. For publication of patient photos, include a statement confirming that consent to publish was obtained.	N/A
14. Report any restrictions on the availability (and/or on the use) of human data or samples.	Only fibroblasts were available for functional studies. Fibroblasts were obtained from only one of the patients. No samples available from the other (Page 27).
15. Report the clinical trial registration number (at ClinicalTrials.gov or equivalent), where applicable.	N/A
16. For phase II and III randomized controlled trials, please refer to the CONSORT flow diagram (see link list at top right) and submit the CONSORT checklist (see link list at top right) with your submission. See author guidelines, under 'Reporting Guidelines'. Please confirm you have submitted this list.	N/A
17. For tumor marker prognostic studies, we recommend that you follow the REMARK reporting guidelines (see link list at top right). See author guidelines, under 'Reporting Guidelines'. Please confirm you have followed these guidelines.	N/A

F- Data Accessibility

18: Provide a "Data Availability" section at the end of the Materials & Methods, listing the accession codes for data generated in this study and deposited in a public database (e.g. RNA-Seq data: Gene Expression Omnibus GSE39462, Proteomics data: PRIDE PXD000208 etc.) Please refer to our author guidelines for 'Data Deposition'.<br><br>Data deposition in a public repository is mandatory for:<br>a. Protein, DNA and RNA sequences<br>b. Macromolecular structures<br>c. Crystallographic data for small molecules<br>d. Functional genomics data<br>e. Proteomics and molecular interactions	Sample preparation, mass spectrometry, data processing and raw data have been deposited to the ProteomeXchange Consortium http://proteomecentral.proteomexchange.org) via the PRIDE partner repository (Vizcaíno et al, 2013) with the dataset identifiers PXD022856 and PXD02285 (Pages 36/37)
19. Deposition is strongly recommended for any datasets that are central and integral to the study; please consider the journal's data policy. If no structured public repository exists for a given data type, we encourage the provision of datasets in the manuscript as a Supplementary Document (see author guidelines under 'Expanded View' or in unstructured repositories such as Dryad (see link list at top right) or Figshare (see link list at top right).	The datasets produced in this study are available in the following database:<br>Complexome profiling data: ProteomeXchange consortium via the PRIDE repository, dataset identifiers PXD022856 and PXD022855.<br>http://www.ebi.ac.uk/pride/archive/projects/PXD022856<br>http://www.ebi.ac.uk/pride/archive/projects/PXD022855
20. Access to human clinical and genomic datasets should be provided with as few restrictions as possible while respecting ethical obligations to the patients and relevant medical and legal issues. If practically possible and compatible with the individual consent agreement used in the study, such data should be deposited in one of the major public access-controlled repositories such as dbGAP (see link list at top right) or EGA (see link list at top right).	This is not compatible with the individual consent agreement used in this study. However, the variants identified in the patient have been submitted to ClinVar (https://www.ncbi.nlm.nih.gov/clinvar/)
21. Computational models that are central and integral to a study should be shared without restrictions and provided in a machine-readable form. The relevant accession numbers or links should be provided. When possible, standardized format (SBML, CellML) should be used instead of scripts (e.g. MATLAB). Authors are strongly encouraged to follow the MIRIAM guidelines (see link list at top right) and deposit their model in a public database such as Biomodels (see link list at top right) or JWS Online (see link list at top right). If computer source code is provided with the paper, it should be deposited in a public repository or included in supplementary information.	N/A

G- Dual use research of concern

22. Could your study fall under dual use research restrictions? Please check biosecurity documents (see link list at top right) and list of select agents and toxins (APHIS/CDC) (see link list at top right). According to our biosecurity guidelines, provide a statement only if it could.	N/A
